# Animal disease traceability: evaluation of simulated foot-and-mouth disease outbreak metrics with implementation of improved contact tracing of cattle

**DOI:** 10.3389/fvets.2026.1804982

**Published:** 2026-05-05

**Authors:** MaRyka Renae Smith, Michael W. Sanderson

**Affiliations:** Center for Outcomes Research and Epidemiology, Department of Diagnostic Medicine and Pathobiology, College of Veterinary Medicine, Kansas State University, Manhattan, KS, United States

**Keywords:** cattle, epidemiology, foot-and-mouth disease, livestock, modelling, traceability

## Abstract

**Introduction:**

In the event of a Foot-and-Mouth Disease (FMD) introduction in the United States (US) animal health officials will need to rapidly trace infected animals and their contacts. Electronic identification (EID) and digital tracing of cattle could improve the speed and accuracy of tracing efforts. This study evaluated the impacts of improving cattle traceability during an FMD outbreak in the US.

**Methods:**

InterSpread Plus (ISP) was used to simulate FMD outbreaks with a US national livestock population file and animal movement parameters. Scenarios were defined by starting location (California, Nebraska, Texas, New Mexico, New York, and Tennessee), farm type (dairy, feedlot, stocker, cow calf), detection day (8, 14, or 21), and tracing level. The tracing levels represented the current speed and accuracy in the US, a partial EID tracing or an ideal EID tracing implementation. A secondary objective investigated decreasing the geographical size of control areas and surveillance zones under the ideal tracing level. We assessed outcome metrics (infected premises (IPs), outbreak duration, and number of farms under surveillance or in control areas) using a calculation of difference between scenarios and bootstrap confidence interval estimation.

**Results and discussion:**

Improved tracing showed the largest potential for decreasing outbreak size when the outbreaks were detected on day 21. The distribution of IPs decreased consistently at the median and beyond with some scenarios showing decreases as low as the 10th percentile. Similar trends were seen in the duration of outbreaks and number of farms in surveillance zones. When the size of control areas and surveillance zones decreased, the number of IPs tended to increase while the number of farms under surveillance decreased in some scenarios and increased in others. Our results support the value of improved cattle traceability that is rapid and accurate for decreasing the risk of large FMD outbreaks in the US. An improved traceability system could also decrease demands for human labor to conduct traces and surveillance during the outbreak, and improve business continuity of producers during and outbreak. More research into the economic trade-offs and EID feasibility are needed to determine the best implementation of EID tracing systems

## 1 Introduction

Tracing the introduction of a disease back to its original herd or animal is not a novel concept. Reports of disease tracing can be found as early as 1275, wherein the source of “rot” in Europe was traced back to a “Spanish ewe” (1). Later, in 1711, the introduction of Rinderpest to a farm in Venice was linked to a stray ox (1). These were not isolated incidents, and many other accounts of the interest in tracing the origin of disease can be found in historical literature (1). A key difference between the historical tracing of animals and modern tracing is the method used to identify animals. Historical traces relied on human recollection of ownership, markings, descriptions, hooves or hide branding, or physical collars or tags (1, 2). In the modern era, these techniques may still be used, but significant advancements have been made in the ability to individually identify animals and trace their origins, namely, electronic identification (EID) and computer record keeping (2, 3). Ultimately, for a disease traceability system to be effective, it needs to perform at the same timescale as animal movement and disease transmission.

In the United States (US), traceability of disease in livestock, especially cattle, has been of interest since the 1900s, with a focus on brucellosis (*Brucella abortus*) and bovine tuberculosis (bovine TB) (4). In 1917, the US Congress initiated the State-Federal Bovine Tuberculosis Eradication Program (5). This program included testing all animals in a herd and slaughtering all reactors. The formal efforts to eradicate brucellosis in cattle began in 1934 with systematic sampling and herd depopulation (6). In the modern day, control of both diseases relies on routine surveillance and tracing the source of infected animals.

The surveillance and traceability efforts for brucellosis and bovine TB have been effective at disease control. Brucellosis has been eliminated from domestic livestock herds in the United States (6). The only herds that remain positive for brucellosis are wildlife in Yellowstone National Park, with occasional infections in herds in the Greater Yellowstone Area (GYA) (6). Bovine TB control efforts have heavily relied on tracing animals identified at slaughter to their herd of origin (7). A recent study investigated the success of these traces and found as high as 83% success in finding the origin farm, with 70% of traces from adult cows resulting in the identification of more infected animals (7). These two example diseases illustrate the impact of tracing in disease control; however, both diseases spread at a very slow rate. Bovine TB-infected animals can take months to years to incubate the bacteria before developing clinical signs (8). Similarly, the incubation period for brucellosis could range from weeks to months (9). The systems can adequately complete traces for controlling the spread of these diseases but are likely insufficient for a rapidly progressing disease such as foot-and-mouth disease (FMD). The failure to quickly trace at-risk contacts during an FMD outbreak is likely to result in continued transmission through animal contacts and larger outbreaks.

Over the years, the eradication programs have evolved to reflect changes in technology and disease-tracing policy. In 2004, the National Animal Identification System (NAIS) was the first iteration of a widespread approach to national animal traceability (10). In 2010, the NAIS program was replaced by the current Animal Disease Traceability (ADT) program (10). The ADT program requires that covered animals moving interstate have an official form of identification and are documented back to the state of origin (10). The ADT program increased traceability for interstate movements for covered classes of cattle, but class exceptions and within-state movements remain under the authority of state and tribal governments (10). In 2013, the final rule, 9 CFR part 86, clarified which animals must be properly identified on Interstate Certificates of Veterinary Inspection (ICVI) and established multiple exempt classes of cattle and bison. These exemptions increase producer compliance but allow many animal movements to be undocumented. It remains that intra-state movements of exempt animals may occur with no traceability beyond optional producer records. In November 2024, an amendment to the final rule required official identification of covered animals to be a permanent tag that is both electronically and visually readable (11). These improvements help fight disease, but in the US, there is room to improve the speed and accuracy of these efforts (12). In many instances of attempted animal tracing, the records are incomplete, which can result in delays or failure to complete a trace (12).

One of the most significant diseases of concern for traceability is FMD. FMD is a highly contagious and readily transmissible disease of cloven-hooved animals. Upon initial FMD infection, animals can become infectious to others within 4 days of exposure (13). FMD virus can be shed in all bodily fluids of infectious animals (13). The potential for transmission from subclinical animals before clinical signs appear, and the transport of latent animals, adds to the need for rapid and accurate tracing of the movements of infected and at-risk animals in the face of an FMD outbreak (14).

In an FMD outbreak in the US, government officials will be tasked with finding all diseased and at-risk animals and identifying where they have been in the previous 28 days. Because of the extensive movement of cattle in the US, these traces could include numerous farms spread widely across many states and counties (15). The use of EID can help capture the identity of an animal and store that information quickly in a digital database. This data capture can occur at the speed of commerce, with some EIDs being readable up to 30 feet away, allowing animals to be scanned as they pass through chutes and gates in markets or on farms (16).

Previous research has shown that rapid tracing can have positive impacts on controlling outbreaks of FMD, but it has been limited to specific regions of the United States (17, 18). A national model in 2023 also found that tracing was beneficial for outbreak control (19). However, that study was limited by reduced animal movements through markets that were not representative of the realities of livestock movement and production systems of the US (20).

Tracing efforts are not the only method animal health officials will use to control an FMD outbreak. Areas of quarantine and movement control, known as control areas and surveillance zones, will be created around infected premises (IPs) to control the spread of FMD (21). Currently, the United States Department of Agriculture (USDA) FMD Response Plan, also known as “The Red Book,” calls for a 10 km radius circle to be drawn around the IP for the control area and a second circle from 10 km to 20 km to be designated the surveillance zone (21). Improving trace accuracy and speed could aid in the rapid creation of these areas and zones and prevent the continued spread of disease beyond the impacted area. Further, improved tracing could reduce the business continuity impacts of an incursion of FMD by decreasing the number of premises that are not infected but face barriers to business continuity, such as limitations in their ability to procure supplies or get their products to market.

The purpose of this project was to simulate FMD outbreaks in the US and assess the potential impacts of more rapid and accurate traceability of cattle using representative US national production system parameters. The primary objective was to compare the number of IPs, outbreak duration, and the number of farms impacted by surveillance efforts across improvements in the tracing of direct contacts of cattle. The secondary objective was to investigate the possibility of reducing the size of control areas and surveillance zones from the current recommendation when an improved traceability system is used.

## 2 Materials and methods

### 2.1 Modeling program

InterSpread Plus (ISP) (Version 6.01.44), a spatially explicit stochastic modeling software, was used to simulate the outbreaks of FMD (22). The program allows for multiple parameters describing animal movement, control efforts, and the transmission of disease. Disease transmission is determined by many parameters, including direct and indirect contacts, airborne transmission, and local spread. To begin a simulated outbreak, users load an “epidemic history” file that includes the farm identification number for the index case(s) and a defined day and farm identification number for the first detection. The outputs from the ISP are plain-text files that can be exported for analysis by statistical software.

### 2.2 Basic parameters and population

The baseline parameter set was provided by the United States Department of Agriculture Center for Epidemiology and Animal Health (USDA-CEAH). The baseline parameter set describes the basics of the US production system and FMD-specific parameters. The parameters for livestock production system behavior were developed with National Animal Health Monitoring System (NAHMS) data and expert opinion when the data were incomplete. The FMD-specific parameters were developed from the current literature on FMD in field and laboratory settings. Expert opinion was also used to fill gaps in knowledge of the FMD virus. A sample of key parameters of interest is in Supplementary material 1.

The population file was generated by USDA-CEAH with information from the National Agricultural Statistics Service (NASS) Census of Agriculture. Census data from 2020 were used for all dairy premises populations, and data from 2012 were used for all other premises. Farms were placed geographically using the Farm Location and Agricultural Production Simulator (FLAPS) to anonymize the data (23). The population file includes beef and dairy cattle, bison, sheep, goats, and swine, with subdivisions by size and specific farm types such as cow-calf, feedlot, and stocker operations. There is also a population of markets and processors with publicly available locations that serve as points of contact for the mixing and dispersal of infected animals. A total of 1,176,200 premises were included in the population. An overview of the population file is in Supplementary material 2.

Livestock herd movements in ISP are completed in three steps using stochastic processes to generate movements and probability distributions of movement distances and destination farms. In the first step, movement from the infected origin farm is generated according to a Poisson process of the expected daily movement rate. If a movement is generated, a shipping distance and a farm type destination are selected, each from a probability distribution. If a destination farm of the chosen farm type is available within that shipping distance, the movement is completed. For movements through markets, the same process is followed to generate a movement, select a shipping distance, and choose a market within that shipping distance band. Then, the model determines a farm type and shipping distance for the final destination of that movement through the market. The movement parameters were entered and tested to ensure that all production types were making shipments to other farms and markets at expected rates. This was confirmed by comparing the observed movement rates to the model parameter distributions, as shown in Supplementary material 3.

The base efforts to control the outbreaks were surveillance, movement restriction, and depopulation. Upon the detection of the first IP, the control measures began. Surveillance measures were implemented in the control areas and in surveillance zones drawn around IPs, increasing the likelihood of detection of a clinical farm. Movements from detected IPs were stopped with 100% efficacy, consistent with the use of state or federal quarantine orders, and other movements from all other farms were decreased. Depopulation was triggered when the farm was detected and was completed within a time period specified for the farm type. Farms are queued for depopulation based on the order of detection and farm type. Each day, the number of farms depopulated is constrained by the daily capacity limit for that farm type. Capacity limits vary with operation type and size, e.g., large farms take more days to depopulate. The details of the depopulation time and time to the completion of transmission potential from that farm are detailed in Supplementary material 1.

### 2.3 Initiation of outbreaks

Outbreaks are started in ISP with an “epidemic history” file that details the unique index farm identification infected on day 1 and the day the first farm becomes detected. To capture a range of detection days, we modeled detection on days 8, 14, and 21. Outbreaks were not regionally limited; every farm and farm type in the US population file was eligible for infection across all scenarios. The model parameters capture the networks of animal trade in the US by randomly selecting movement destinations according to production type and defined distance probability distributions. This characteristic of the model helps represent the spatial relationships that may drive outbreak spread in a real-world outbreak. Furthermore, allowing all species to become infected and transmit is consistent with biological expectations and yields more realistic simulated outbreaks.

To gain insights into multiple regions of the US and multiple farm types, eight locations and four different farm types were selected. USDA NASS census data, research interest, and published literature were used to identify the areas of interest in each region. The index cases are detailed in Table 1.

**Table 1:** Details for index case selection.

State	Farm type (herd size)	Justification
California	Dairy (1,386)	California is the top dairy-producing state in the US (32). The selected farm was in the approximate location of Tulare County. Tulare County has the most dairy cattle in California (33).
New York	Dairy (1,859)	New York is the third-largest dairy-producing state in the US (34). The selected farm was in the approximate location of Steuben County in rural western New York.
Texas	Feedlot (37,693)	The Texas Panhandle has over 100,000 head of cattle per county (35). A large feedlot was selected from that region. The Texas panhandle is also a common destination of cattle imported into the US (36, 37).
New Mexico	Stocker (6,053)	A stocker farm in the south-central region of New Mexico was selected based on models from Gorsich et al. (36, 37) that estimated movement of imported cattle in the southern US.
Nebraska	Feedlot (6,616)	A feedlot in northeastern Nebraska was selected to represent the Midwest for its proximity to other major livestock-producing states, Kansas and Iowa.
Nebraska	Cow–calf (642)	Nebraska has approximately 6 million cattle and calves (38). A farm in central NE was selected for its proximity to many beef-producing counties in the state.
Tennessee	Dairy (694)	Tennessee was selected to represent the southeastern US. Tennessee ranks 33rd for dairy production (39). The selected farm was in the approximate location of White County, which is in central Tennessee and has the 5th most milk production (40).
Tennessee	Cow–calf (386)	Tennessee was selected to represent the southeastern US. Tennessee ranks 22nd for cattle and calves (39). The selected farm was in the approximate location of Lincoln County, which has the most cattle and calves (41).

### 2.4 Scenario specific parameters

#### 2.4.1 Tracing parameters

In ISP, the tracing parameter is defined by the speed-to-completion in days, the likelihood of completion, and the likelihood that the movement is not traced (trace failure). Some parameters define the speed and accuracy of tracing all species included in the population file. The tracing parameters of interest in this project consisted of the current tracing ability, a partial implementation of cattle EID tracing ability, and an ideal cattle EID implementation. The tracing parameters for all non-cattle species were held constant across the scenarios. The tracing levels were developed with input from the USDA-CEAH and the United States National Animal Disease Traceability and Veterinary Accreditation Center (USDA-NADTVAC). The data for current tracing parameterization were primarily derived from real-world brucellosis and bovine tuberculosis tracing efforts, along with expert opinion from USDA-NADTVAC.

The “current” level of tracing depended on the production type. For beef cattle traces, 85% were successfully completed (15% trace failure). Of successful traces, 10% were completed within 2 days, 41% within 4 days, and 98% within 10 days. Successful completion of dairy cattle tracing was 85% in dairy heifer calf units and 87.5% in all other dairy farm types. Of successful dairy traces, 29% were completed within 2 days, 77% within 4 days, and 98% within 10 days.

Partial implementation of EID-based tracing improved the current tracing level but did not achieve the complete capability of an entirely EID-based system. Beef and dairy cattle tracing speed improved with 52% of traces being completed within 2 days, 97% within 5 days, and 99% by day 7. For all cattle, 90% of traces were completed.

The “ideal” implementation of EID-based tracing achieved a 99% success rate, with all traces completed within 1 day for all beef and dairy cattle. The full details of the traceability parameters are provided in Supplementary material 4.

#### 2.4.2 Zones

The standard control area and surveillance zones used for this project are those described in the USDA FMD Red Book, in which the control area has a 10 km radius from the detected IP and the surveillance zone extends 10 km beyond that (10 km to 20 km from the IP) (21). The standard control area was modeled with all 3 tracing levels. We also evaluated a “reduced” zone size at the ideal level of tracing with a control area radius around a detected IP of 7 km and a surveillance zone that extended another 7 km beyond that (7 km to 14 km from the IP).

### 2.5 Analysis

Each iteration of ISP models the introduction and outbreak of FMD. The model completes the outbreak 60 days after the last detection. The outputs from ISP are plain-text files, with each change in a specific farm’s outbreak state (e.g., infected, detected, depopulated) reported on a new line. In the surveillance zone and control area outputs, a farm is only reported on the first instance of being in a control area or surveillance zone. If the farm is later included in an overlapping zone or area before the expiration of the first zone, it will not be reported again, as its farm state did not change. If a farm were in an area or zone that expired and a new zone or area was drawn subsequently, it would be counted again. The number of markets and processors is not included in the count of IPs reported in this analysis, but they were included as infected and capable of transmitting infection during the simulation of the outbreak. All analyses and data manipulation were performed using R version 4.1.1 and R Studio 2024.09.0 (24, 25).

The number of IPs was calculated as the total number of farms of any species that were ever infected during a given iteration, regardless of the detection status of the farm. The duration of an outbreak was the time from outbreak initiation to the day of final detection. The number of farms impacted by control areas and surveillance zones was calculated by the number of farms that were ever in a control area or surveillance zone. Farms in control areas or surveillance zones may never have become infected, but they were subject to the control measures in those areas or zones.

Using the rogme R package, the shift function was used to calculate differences between selected scenarios (26). For our primary objective, we compared current tracing to partial EID and ideal EID tracing. For the secondary objective, we compared ideal tracing with standard (current Red Book) control areas and zones to ideal tracing with reduced area and zone sizes. For all comparisons, we compared predetermined percentiles: 10th, 25th, median, 75th, and 90th, and utilized bootstrap sampling (*n* = 2,000) to estimate a 95% confidence interval for the difference at each percentile.

We tested each introduction-site scenario for convergence of the number of IPs using current tracing and day 21 detection. The scenarios were run for an initial 100 iterations. The total number of IPs in each iteration was calculated, and then the median and 90th-percentile ranked outbreaks across 100 iterations were compared to the median and 90th-percentile outbreaks at 90 iterations. If there was less than a 5% change in both metrics, the scenario was considered converged. In small outbreaks, the percent change could exceed 5%, but if there were fewer than 10 farms between the 90th and 100th iterations, the model was still considered converged. If a scenario did not converge, another 50 iterations were run, and the median and 90th percentile at 150 iterations were compared with those at 100 iterations. If the scenario met the 5% change or less at 150 iterations, the 100 iterations were considered sufficient for convergence.

## 3 Results

### 3.1 Convergence

All scenarios except the feedlot introduction in Nebraska converged at 100 iterations, with the median and 90th percentile number of IPs. After an additional 50 iterations, the feedlot introduction in Nebraska had less than 5% change in IPs from 100 to 150 iterations. Thus, the results from 100 iterations were used in all scenarios.

### 3.2 General results

The simulated outbreaks tended to have a clear divergence in the number of IPs (outbreak size) between those that started in a cow–calf or stocker operation and those that started in a dairy or feedlot, across the other variables. The scenarios that began in a New Mexico stocker operation, or a cow–calf operation in Nebraska or Tennessee, had smaller outbreaks and often failed to sustain transmission beyond a few farms. Specifically, across the introduction sites, there were at least 29 and up to 39 iterations in which fewer than 10 farms were infected using day 21 detection and current tracing. These small outbreaks did not respond as markedly as the larger outbreaks to improvements in tracing. For the remainder of this report, we will refer to the groups of outbreaks collectively as large (feedlot and dairy) or small (cow–calf and stocker).

The day that outbreaks were detected was also an influential predictor of outbreak size. Across all introduction sites and tracing levels, the outbreaks detected on day 8 were the smallest, followed by those on day 14 and day 21. The outbreaks detected on day 8 were seldom remarkable in their response to changes in tracing level until they reached the 75th percentile or larger. The outbreaks detected on day 14 were more variable in their responses to tracing than those detected on day 8, but the changes were still modest. The limited size of the outbreaks that were detected early likely reduced the opportunity to implement tracing in the control efforts. The impact of improved tracing was substantial for outbreaks detected on day 21. For these reasons, the day 21 outbreaks will be the focus of the remainder of the results section of this report. Key outbreak metrics are provided in figures. Detailed data for all day 21 outbreak metrics are included in tabular format in Supplementary material 5. The outbreak metrics from those detected on days 8 and 14 are included in Supplementary material 6.

### 3.3 Level of tracing

#### 3.3.1 Infected premises (IPs)

**Figure 1:**
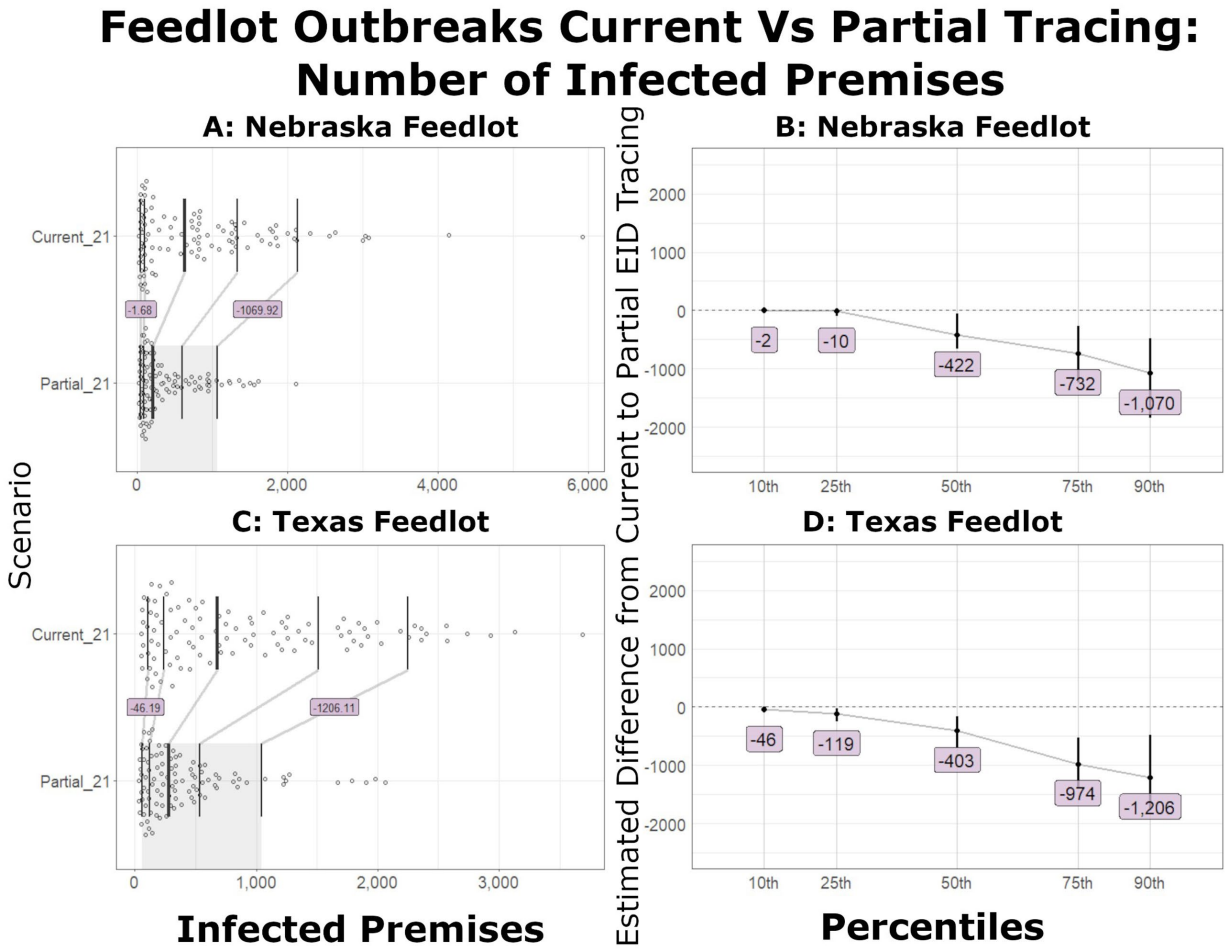
Number of infected premises for outbreaks that began in feedlots. **(A)** Shift function overlaid on scatterplots of the number of IPs in Nebraska outbreaks detected on day 21 with current and partial tracing. **(B)** The estimated difference between the partial and current level of tracing and the bootstrap confidence interval (vertical line) for Nebraska outbreaks. **(C)** Shift function for Texas outbreaks. **(D)** Bootstrap difference confidence intervals for Texas outbreaks.

**Figure 2:**
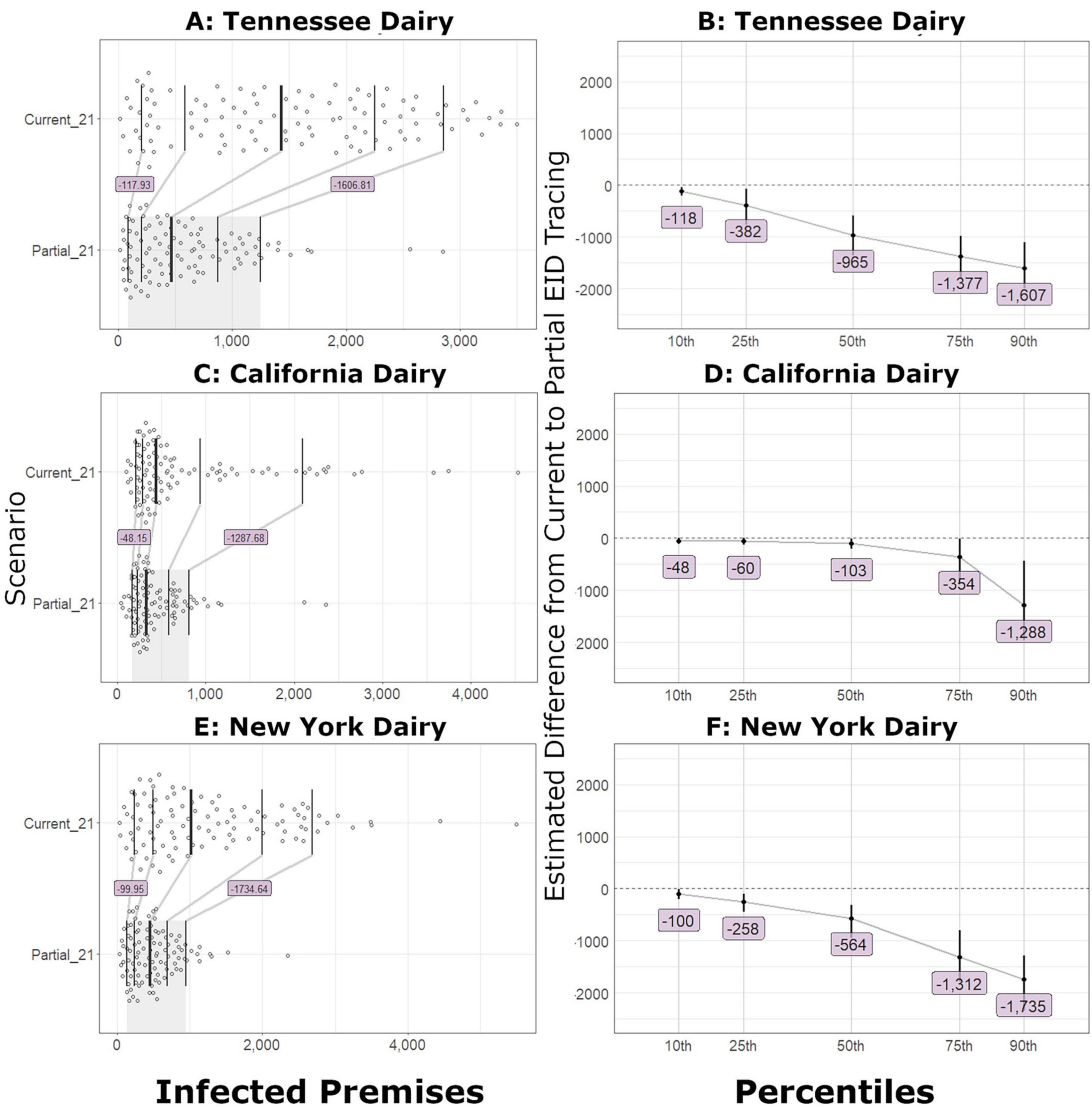
Number of infected premises for outbreaks that began in dairies. **(A)** Shift function overlaid on scatterplots of the number of IPs in Tennessee outbreaks detected on day 21 with current and partial tracing. **(B)** The estimated difference between the partial and current level of tracing and bootstrap confidence interval (vertical line) for Tennessee outbreaks. **(C)** Shift function for California outbreaks. **(D)** Bootstrap difference confidence intervals for California outbreaks. **(E)** Shift function for New York outbreaks. **(F)** Bootstrap difference confidence intervals for New York outbreaks.

In large outbreaks, the number of IPs decreased markedly with improved tracing. In all scenarios, the estimated number of IPs decreased when partial EID tracing was compared to current tracing. The bootstrap confidence interval (CI) for the differences excluded zero at the 10th percentile for Tennessee and New York dairy and Texas feedlot introductions. The California dairy introduction difference CI excluded zero at the 25th percentile, and the Nebraska feedlot introduction difference CI excluded zero at the median. Figure 1 shows the distribution, shift function, and bootstrap difference confidence intervals for outbreaks that started in feedlots. Figure 2 depicts the distribution, shift function, and bootstrap difference confidence intervals of the outbreaks that started in dairies. The most notable changes were in New York and Tennessee dairy introduction scenarios, where 100–120 IP decreases were seen at the 10th percentile and over 1,600 fewer IPs at the 90th percentile.

**Figure 3:**
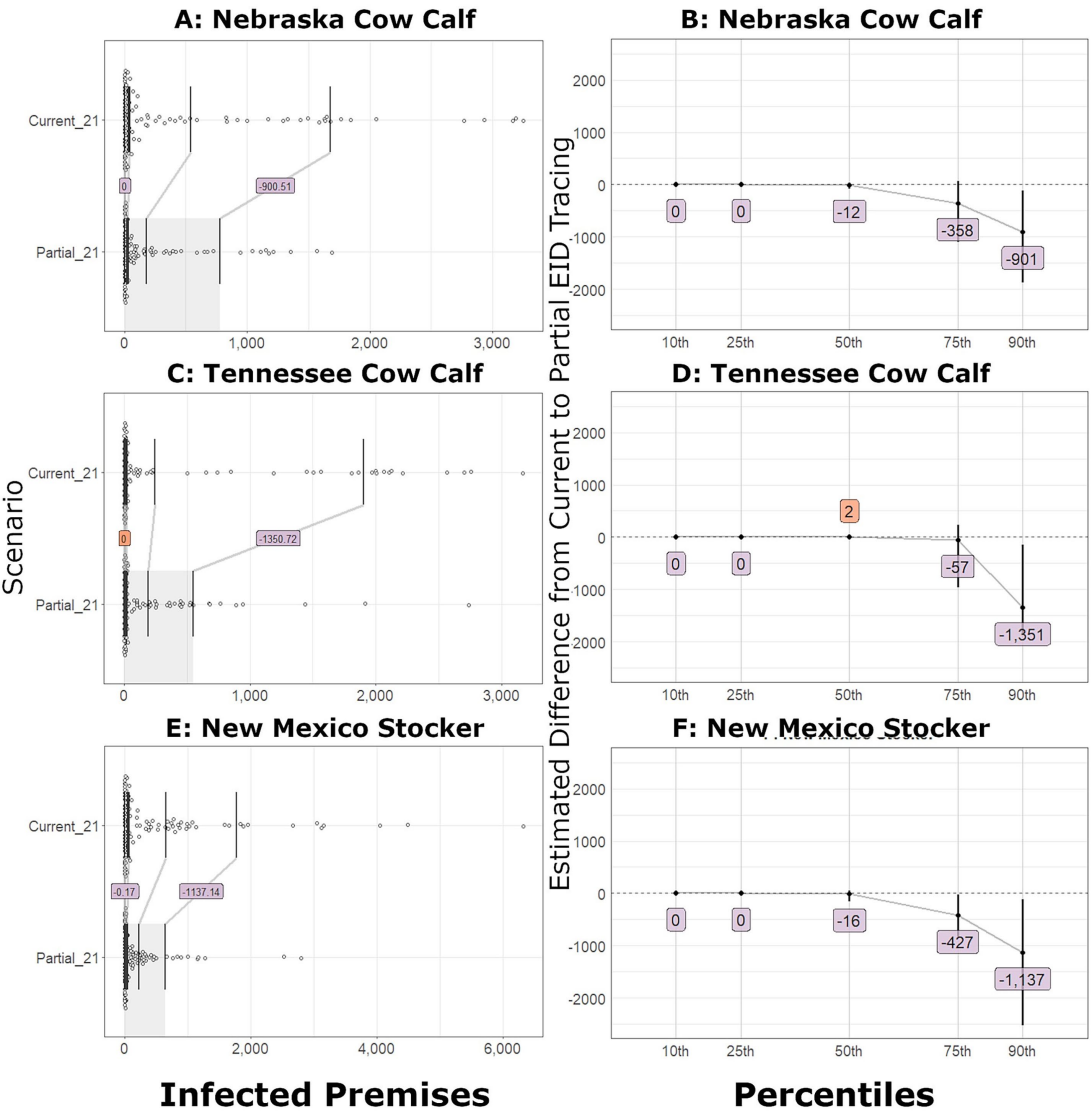
Number of infected premises for outbreaks that began in small outbreak scenarios. **(A)** Shift function overlaid on scatterplots of the number of IPs in Nebraska cow–calf outbreaks detected on day 21 with current and partial tracing. **(B)** The estimated difference between the partial and current level of tracing and bootstrap confidence interval (vertical line) for Nebraska cow–calf outbreaks. **(C)** Shift function for Tennessee cow–calf outbreaks. **(D)** Bootstrap difference confidence intervals for Tennessee cow–calf outbreaks. **(E)** Shift function for New Mexico stocker outbreaks. **(F)** Bootstrap difference confidence intervals for New Mexico stocker outbreaks.

In small outbreaks, the number of IPs decreased with improved tracing, but not to the same magnitude as the large outbreaks. When partial EID tracing was implemented, the number of IPs at the 10th and 25th percentiles was similar to the current level of tracing. At the median outbreak, the predicted decrease in the number of IPs was less than 20 farms across all introduction sites. In outbreaks starting in a New Mexico stocker operation, the 75th-percentile outbreak had an estimated 427 fewer IPs, and the bootstrap difference confidence interval excluded zero. The confidence interval of the partial and current IP difference for the two cow–calf introduction scenarios did not exclude zero until the 90th percentile, when the predicted decrease was approximately 900–1,300 farms. Figure 3 illustrates the shift function and differences for the partial EID compared to the current tracing for small outbreak scenarios.

When comparing the current implementation with the ideal implementation of EID tracing, the resultant number of IPs in large outbreak scenarios decreased dramatically. Bootstrap confidence intervals for the estimated differences excluded zero at the 10th percentile outbreak in all large outbreak scenarios except for the Nebraska feedlot introduction, where zero was excluded at the 25th percentile. The outbreaks in dairies were estimated to decrease by 140 to over 1,200 IPs at the median and by up to 2,200 fewer IPs at the 90^th^ percentile.

When ideal EID tracing was compared with the current approach, small outbreaks showed a pronounced decrease in the number of IPs. In Nebraska cow–calf and New Mexico stocker introductions, the difference confidence interval excluded zero at the 75th percentile, with approximately 400 fewer IPs and over 1,300 fewer IPs at the 90th percentile of the outbreak. Outbreaks that began in a Tennessee cow–calf reached a confidence interval that excluded zero at the 90^th^ percentile with an estimated 1,765 fewer farms infected.

#### 3.3.2 Duration of outbreaks

The changes in duration of outbreaks with improved traceability were less marked than the changes in the number of IPs. Similar to IPs, changes were small for the outbreaks with detection on days 8 and 14 (Supplementary material 6) but more notable on day 21. The overall trend was a decrease in the time to the last detection of an IP, but the magnitude and significance of that decrease were highly variable.

**Figure 4:**
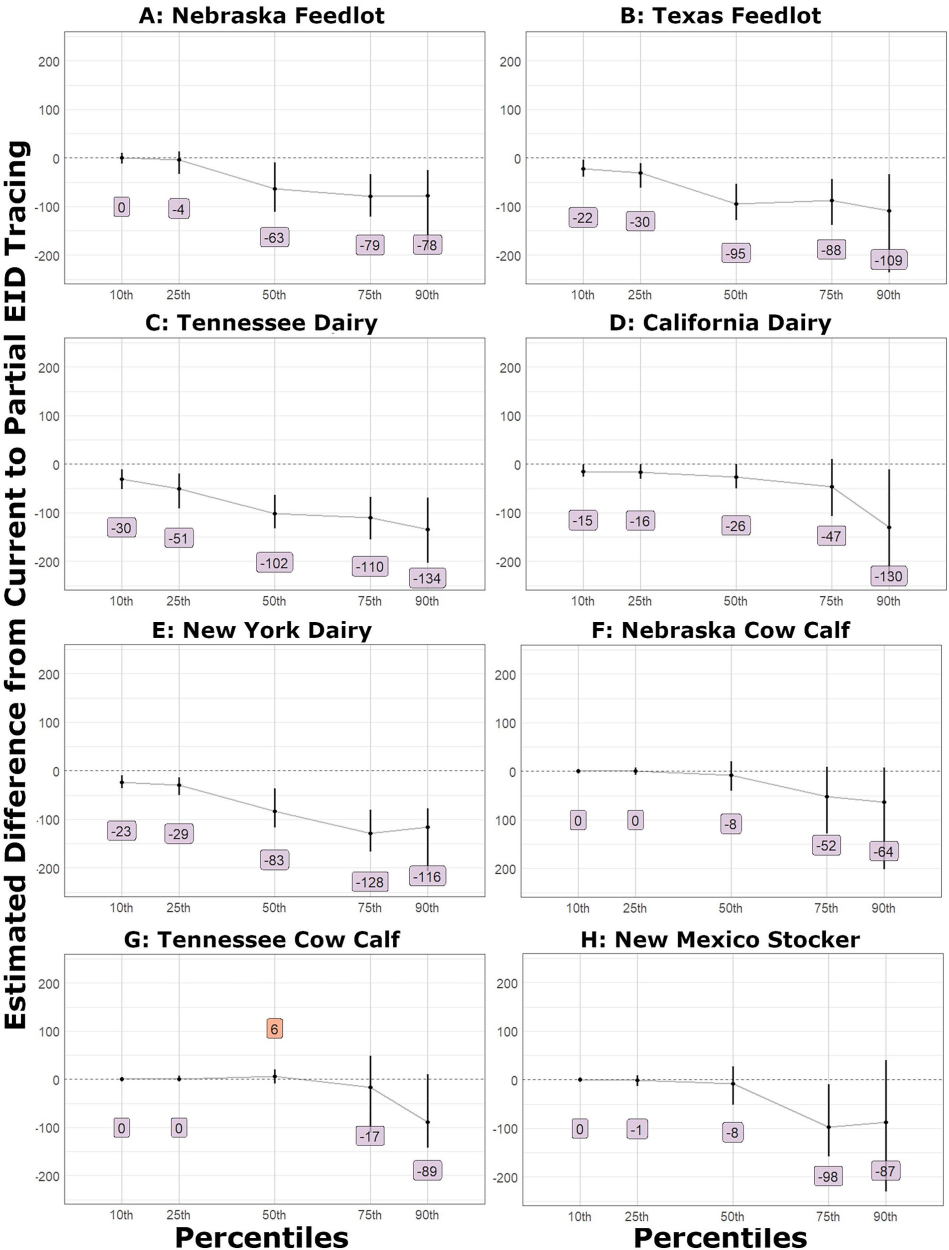
Shift function plots for duration of outbreaks in days. The estimated difference and the bootstrap confidence interval: **(A)** Nebraska feedlot, **(B)** Texas feedlot, **(C)** Tennessee dairy, **(D)** California dairy, **(E)** New York dairy, **(F)** Nebraska cow calf, **(G)** Tennessee cow calf, **(H)** New Mexico stocker.

In the large outbreak scenarios that utilized partial EID tracing, the duration of outbreaks was consistently decreased based on bootstrap difference confidence intervals. The difference confidence interval excluded zero at the 10th percentile except for the Nebraska feedlot. At the median, the Nebraska feedlot had an estimated 63 fewer days of duration, and the confidence interval did not include zero. The largest decrease was observed in the Tennessee dairy scenario 90th-percentile outbreak, with a 134-day reduction in duration. For the small outbreak scenarios with partial EID implementation, the estimated differences indicated a decrease in outbreak duration, but the confidence interval did not exclude zero or any percentile in any location. These results are detailed in Figure 4.

In large outbreak scenarios with ideal EID tracing, the decreased duration of the outbreaks was evident at the 10th percentile for all but the Nebraska feedlot scenario, which was decreased at the 25th percentile by approximately 16 days. Median reduction in duration ranged from 41 to 139 days. The small outbreaks were not notably shorter until the 75th percentile for the Nebraska cow–calf and New Mexico stocker scenarios and the 90th percentile for the Tennessee cow–calf scenario. The small outbreaks were 135–175 days shorter at the 90th percentile with ideal tracing.

#### 3.3.3 Number of farms under surveillance

The number of farms under surveillance includes those that were in control areas and surveillance zones. They serve as a representation of the farms affected by outbreak control efforts, even if they are not infected.

In the large outbreak scenarios, the implementation of partial EID tracing resulted in significant decreases in the number of farms in control areas (0–10 km from IP) at the 10th percentile for the dairy introductions and at the median for the feedlot scenarios. The largest change at the median outbreak was from outbreaks starting in a Tennessee dairy, where an estimated 68,000 fewer farms were in control areas.

**Figure 5:**
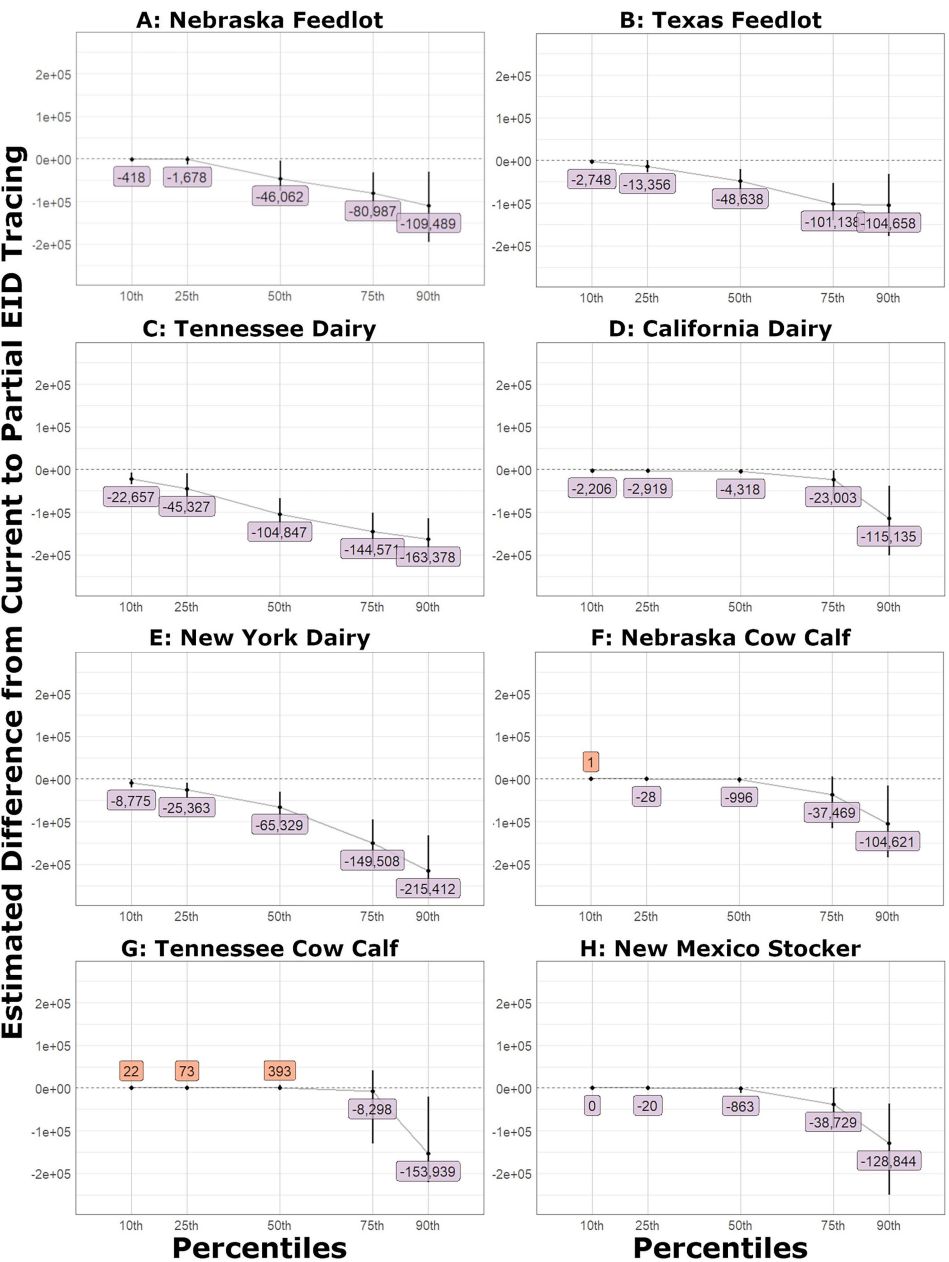
Shift function plots for the number of farms in surveillance zones. The estimated difference and the bootstrap confidence interval. **(A)** Nebraska feedlot, **(B)** Texas feedlot, **(C)** Tennessee dairy, **(D)** California dairy, **(E)** New York dairy, **(F)** Nebraska cow calf, **(G)** Tennessee cow calf, **(H)** New Mexico stocker.

In the surveillance zones (10–20 km from an IP), this same trend continued with significant decreases at the 10th percentile for dairy introductions and the median outbreak for feedlot introductions. The largest overall decrease was over 200,000 fewer farms in the surveillance zones at the 90th-percentile outbreak that began in a New York dairy. The data for the number of farms in surveillance zones with partial EID implementation is shown in Figure 5.

In the small outbreak scenarios, the use of partial EID tracing made very small decreases in the number of farms in control areas and surveillance zones that were not significant until the 90th percentile for most scenarios. The outbreaks that began in New Mexico stockers reached a significant change at the 75th percentile outbreak, with an estimated 23,000 fewer farms in control areas and 38,000 farms in surveillance zones. This data is shown in Figure 5.

When ideal tracing was implemented, the decrease in the number of farms in control areas was sufficient to exclude zero from the confidence interval beginning at the 10th percentile for all introduction sites except the Nebraska Feedlot (Supplementary material 5). At the median, there were approximately 33,000 fewer farms in control areas in outbreaks that began in a Nebraska feedlot. The dairy introductions saw 79,000 to 164,000 fewer farms in control areas at the 90th percentile of the outbreak. The trend continued in the surveillance zones with all locations except Nebraska feedlots reaching significant decreases at the 10th percentile and Nebraska feedlot reaching significance at the median outbreak. The New York dairy scenario remained the site with the largest change, having an estimated 271,000 fewer farms in surveillance zones at the 90th percentile when ideal EID use was implemented.

In the small outbreaks, the effect of ideal EID tracing on farms under surveillance was less remarkable until the 75th to 90th percentile of the outbreaks. Across the 3 small outbreak scenarios, at the 90th percentile, there was an estimated decrease of 90,000 or more farms impacted by control areas and 150,000 fewer farms impacted by surveillance zones (Supplementary material 5).

### 3.4 Reduction of control areas and surveillance zone size

The reduced control area and surveillance zone scenario was combined with ideal EID tracing for all introduction sites. These scenarios address the secondary objective, which was to assess the potential for further decreasing the resources required for outbreak control. All scenarios in this objective utilize ideal EID tracing parameters and day 21 detection.

**Figure 6:**
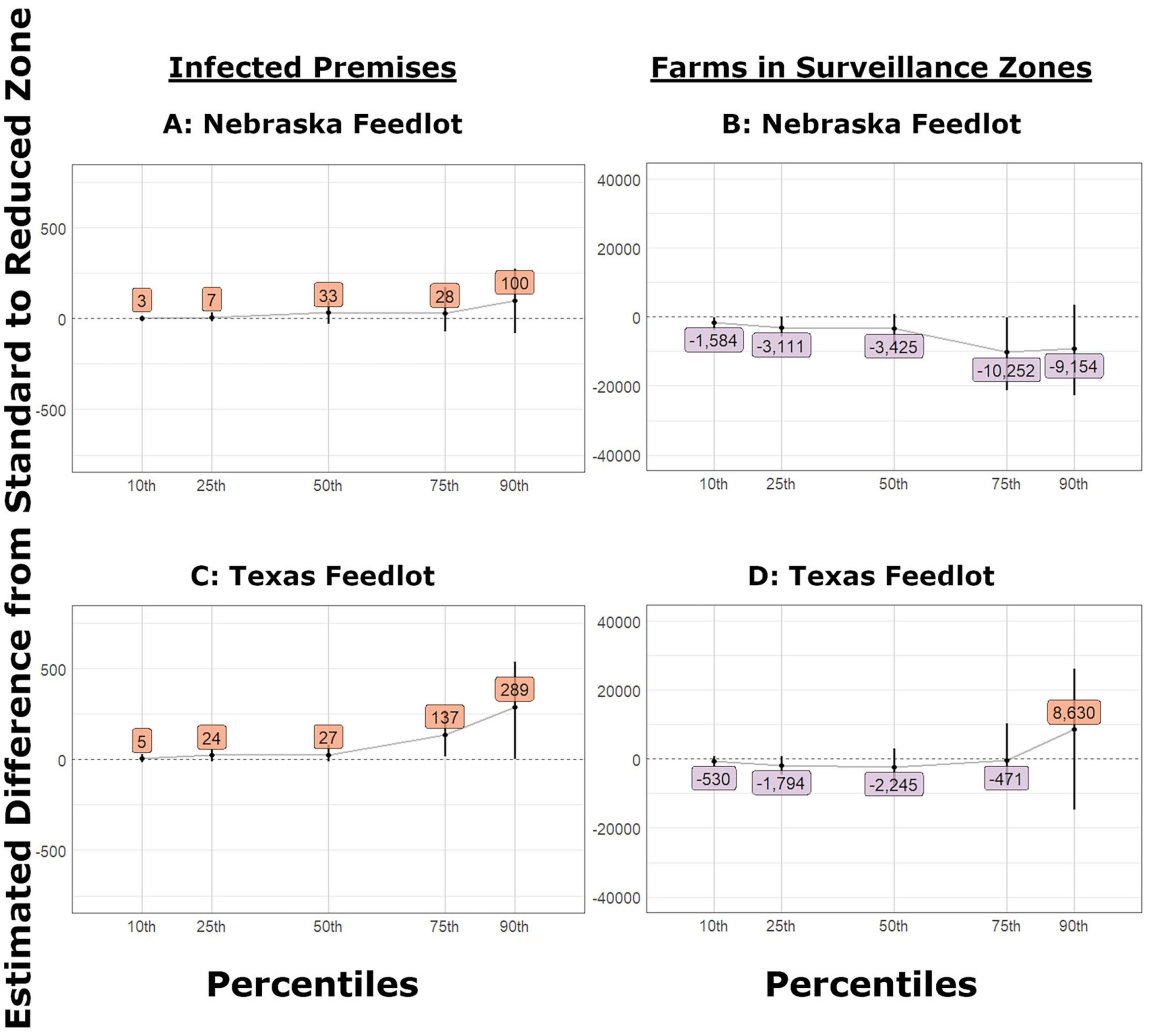
Change in number of IPs and farms in surveillance zones in outbreaks that began in feedlots and used reduced control area and surveillance zone radii. The estimated difference between the partial and current level of tracing and bootstrap confidence interval (vertical line) for: **(A)** Nebraska Feedlot outbreaks infected premises, **(B)** Nebraska Feedlot outbreaks farms under surveillance, **(C)** Texas Feedlot outbreaks infected premises, **(D)** Texas Feedlot outbreaks farms under surveillance.

**Figure 7:**
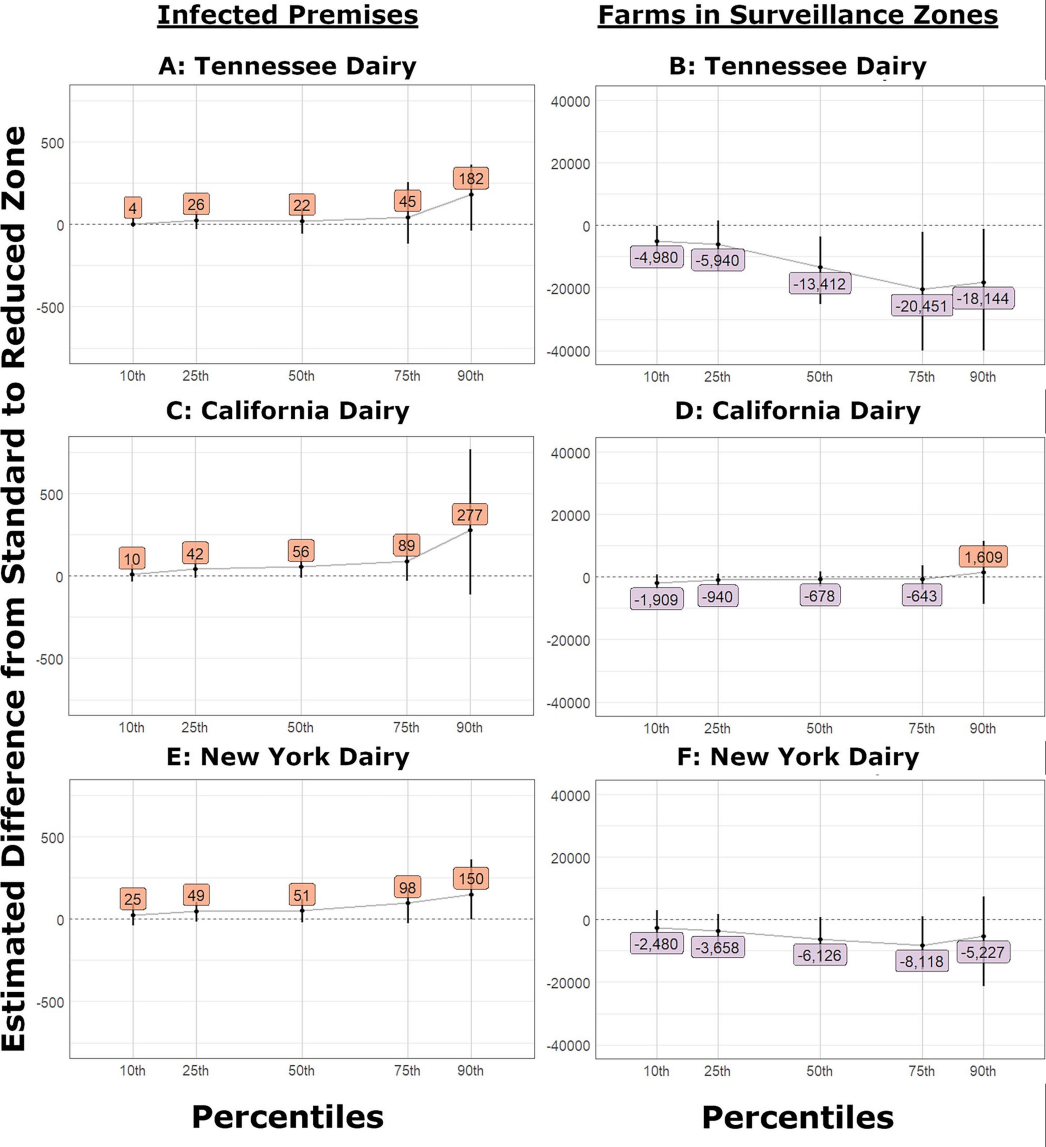
Change in number of IPs and farms in surveillance zones in outbreaks that began in dairies and used reduced control area and surveillance zone radii. The estimated difference between the partial and current level of tracing and bootstrap confidence interval (vertical line) for: **(A)** Tennessee Dairy outbreaks infected premises, **(B)** Tennessee Dairy outbreaks farms under surveillance, **(C)** California Dairy outbreaks infected premises, **(D)** California Dairy outbreaks farms under surveillance, **(E)** New York Dairy outbreaks infected premises, **(F)** New York Dairy outbreaks farms under surveillance.

**Figure 8:**
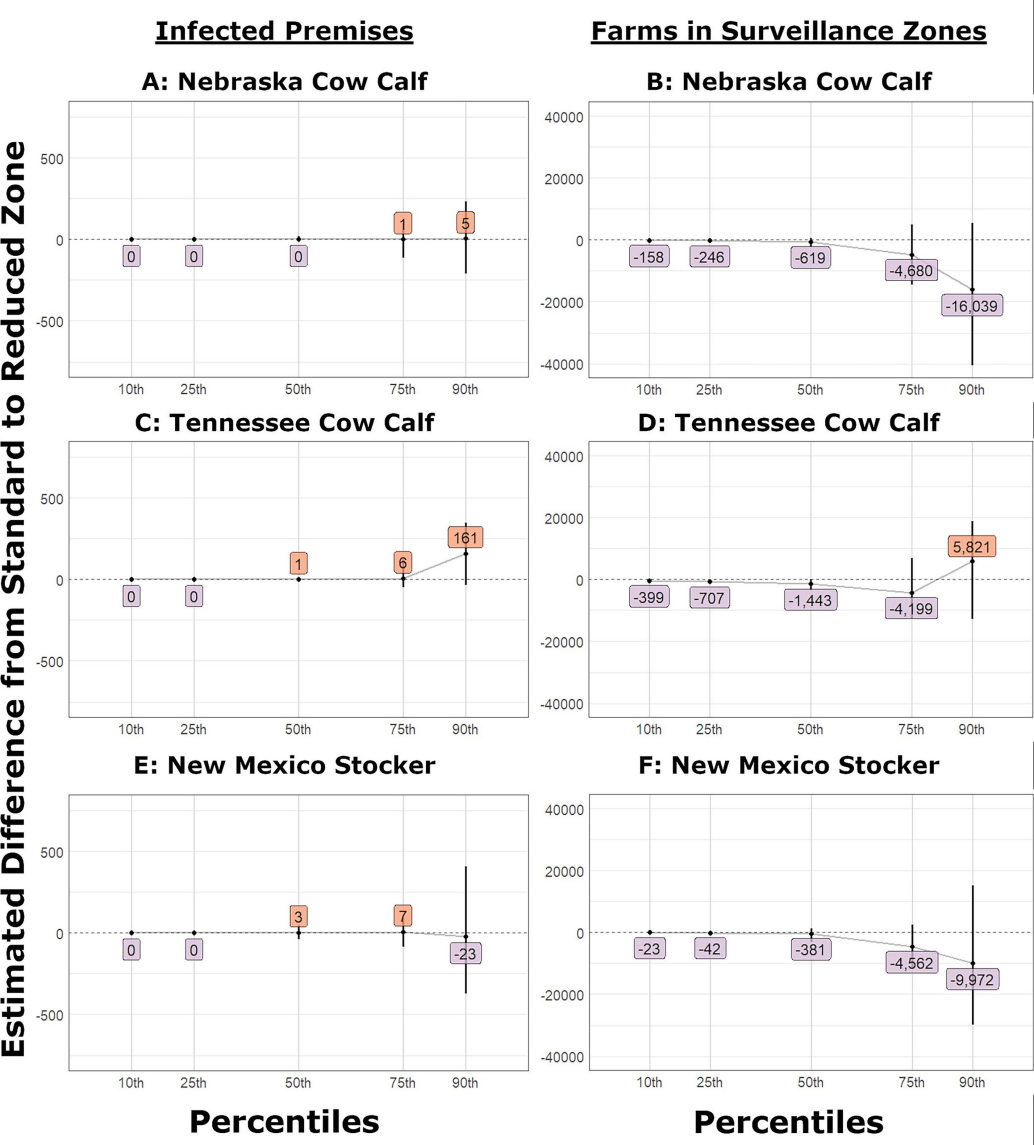
Change in number of IPs and farms in surveillance zones in small outbreak scenarios that used reduced control area and surveillance zone radii. The estimated difference between the partial and current level of tracing and bootstrap confidence interval (vertical line) for: **(A)** Nebraska Cow Calf outbreaks infected premises, **(B)** Nebraska Cow Calf outbreaks farms under surveillance, **(C)** Tennessee Cow Calf outbreaks infected premises, **(D)** Tennessee Cow Calf outbreaks farms under surveillance, **(E)** New Mexico Stocker outbreaks infected premises, **(F)** New Mexico Stocker outbreaks farms under surveillance.

Across all scenarios, the estimated number of IPs increased marginally when the areas under surveillance were decreased. In the large outbreak scenarios, this increase was significant in the Texas feedlot scenario at the 75th percentile and 90th percentile (Figure 6). The New York dairy scenario at the 90th percentile was very close to excluding zero (Figure 7). The small outbreaks had similar numbers of IPs with reduced control and surveillance zones. The 90th percentile of the New Mexico stocker introduction showed a decrease in the number of IPs with a reduction of zone size; this is likely an artifact of stochastic processes in the model. The wide confidence interval supports a high uncertainty around this estimation (Figure 8). The impact of reducing the control area and surveillance zones on the duration of outbreaks was less predictable. In the majority of outbreaks, regardless of the introduction site, the change in duration was minimal. When outbreaks began in a Nebraska feedlot, Tennessee dairy, or New Mexico Stocker, at the 90th percentile, the increase in duration was less than 30 days. The Texas feedlot in the 90th-percentile outbreak had the largest estimated change, with up to 70 more days to control the outbreak when the area and zone sizes were decreased. The California dairy introduction had the next-largest number, with 48 more days of the outbreak at the 90th percentile.

The change in the number of farms under surveillance was the most remarkable of the metrics we evaluated. In most scenarios, there was a slight decrease in the number of farms under surveillance in both the control areas and surveillance zones in the smaller outbreak percentiles. The surveillance zone shift functions are shown in Figures 6–8.

When looking at the number of farms in control areas, the Tennessee dairy introduction showed a significant decrease, even at the 90th percentile. Conversely, in the Tennessee cow–calf and Texas feedlot scenarios, an estimated increase with a wide confidence interval was observed at the 90th percentile. When looking at farms in surveillance zones, a similar trend was observed; most smaller outbreaks decreased, and some larger outbreaks had the potential to affect more farms. The Tennessee dairy experienced a significant decrease in the number of farms affected at the 90th percentile. Conversely, the Texas feedlot and Tennessee cow–calf are joined by the California dairy as sites that have increased estimated farms at the 90th percentile. The data for farms in surveillance zones are shown in Supplementary material 5.

## 4 Discussion

This study’s primary objective was to assess the impacts of three different levels of US cattle traceability in simulated FMD outbreaks. We simulated a variety of introduction sites and farm types to estimate the potential range of effects on outbreak size, duration, and the total number of farms that were under surveillance. The collective result of the simulations was a decrease in the likelihood of large outbreaks across introduction sites and detection days. This was consistent with our hypothesis that increasing the speed and accuracy of tracing could improve the ability to surveil at-risk premises and detect their infection more quickly, resulting in enhanced implementation of disease control. Across all simulated scenarios, improved traceability reduced the 90th percentile of the outbreak and, in most cases, to values below the median or lower percentiles. In smaller outbreaks, there was less opportunity for improved tracing to be impactful in shifting the median or 10^th^ percentile metrics. When outbreaks were larger, improved tracing reduced the outbreaks dramatically, in some cases even down to the 10th percentile. This is consistent with the previous report Smith and Sanderson (19) but at a greater magnitude. The value of traceability accrues from both the percentage of traces completed and the speed with which they are completed. This is particularly important for a disease like FMD, which is rapidly transmitted between animals and farms. Delayed completion of traces for FMD results in extended periods of infectivity for undetected and unquarantined herds and delays in implementation of control efforts. These delays may result in increases in outbreak size, as supported by the results of this study.

Our results were extended beyond the implementation of an imagined ideal EID traceability program to include a partial implementation with improved but imperfect tracing. This suggests that even the partial implementation of an EID program could reduce the risk of large, disastrous outbreaks. The partial implementation of an EID program could be more achievable in the short term. Allowing more reasonable goal setting by producers and animal health officials while still providing substantial benefit in the truncation of FMD outbreak risk.

The potential value of improved traceability includes decreased numbers of IPs, decreased duration of the outbreak, and a decrease in the number of premises that were under surveillance during the outbreak. The number of IPs is a key indicator of the cost of an outbreak due to costs to producers and the government for quarantine and depopulation efforts (27, 28). Duration of the outbreak is also an important metric for outbreak cost as it relates to the potential duration of time the country would be excluded from international markets and incur economic export losses (28). The number of premises under surveillance in the control area and surveillance zone also adds to outbreak costs, both through government surveillance costs and, more substantially, through costs borne by premises under surveillance. Farms within control areas and surveillance zones are subject to limitations that impact their daily operations and business continuity. This could include a decreased ability to move animals to market promptly, increased difficulty in acquiring feed and consumables, and costs associated with biosecurity efforts. To maintain the operational viability of the livestock industries, less interruption of business continuity in the process of outbreak control is important. Balancing the need for robust outbreak control with ongoing business continuity is challenging. Improved traceability has the potential to enhance control, reduce the number of farms affected by control efforts, and increase overall industry business continuity.

The secondary objective of this study was to explore the effects of decreasing the size of control areas and surveillance zones with the implementation of ideal EID traceability. The hypothesis for this objective was that increased speed and accuracy in tracing could enable smaller surveillance areas, thereby decreasing resource requirements and increasing business continuity. The potential cost savings from eliminating the human labor required to conduct surveillance and improve continuity of business operations could be highly valuable during and after an outbreak. Interestingly, the results of this project suggest that while a smaller control area and surveillance zone may be beneficial in some outbreaks, in others, the number of farms that must be surveilled increases. Furthermore, across scenarios, as the control area and surveillance zone sizes were decreased, the number of IPs increased to some degree. We suspect that in scenarios where the number of farms under surveillance increased, this was due to an increase in the number of IPs, resulting in more control areas and surveillance zones being established, and in the total area under surveillance and the total number of farms in control areas and surveillance zones. The mixed results found here do not support the claim that reduced zone sizes reliably benefit by decreasing the total number of farms under surveillance, and support the current recommendation for zone size.

The value of early detection was evident across all scenarios. Consistently, across locations and tracing levels, early detection of the outbreak was highly impactful in determining the outbreak size. Outbreaks detected on day 8 were smaller than those on days 14 and 21, respectively. These results are consistent with previous findings by Smith and Sanderson (19), Carpenter et al. (29), and Bates et al. (30). This supports the continued need for producer and veterinarian education to support early identification. While the value of improved tracing following identification of outbreaks by day 8 is limited, such early detection may be optimistic, and detection by day 14 or 21 may better reflect reality and the potential value of traceability.

Given the small size of many outbreaks, tracing efforts had fewer opportunities to affect their course. These outbreaks were consistent with published literature from other models of the US. For example, Tsao et al. (31), in their 2020 publication, found that the areas of Tennessee and New Mexico that we selected had very few resultant IPs when their models were seeded in those counties.

This study does not provide a full explanation for the observed differences in the introduction scenarios. Comparison of the outputs is confounded by region and index farm type. Additional scenarios with multiple index farm types within each region would help sort out this effect. We expect interactions among the farm type, direct and indirect contact rates, farm density in an area, and the local disease spread parameters. For example, dairies and feedlots are parameterized to have much higher rates of indirect contact than stockers or cow–calf operations. Moreover, dairies have a higher direct-contact rate. This study evaluated improved tracing for cattle in a US FMD outbreak. Improved tracing for additional species could increase the overall impact on the FMD outbreak. Despite these limitations, the results are consistent across regions and index farm types. The results are based on a synthetic population of farms generated from aggregate data. To the extent that the synthetic farm file deviates from the true farm and geographic distributions, the results could be biased. However, the consistent effect across different regions suggests the general results are robust. Finally, the FMD parameters used represent aggregate estimates of infectivity based on the referred literature, which is predominated by a few strains that have been more thoroughly researched. An FMD strain with lower relative infectivity might result in a less notable tracing value, similar to the effect noted here in smaller outbreaks. Similarly, an FMD strain with higher relative infectivity might yield a more notable tracing value, similar to the effect noted here in larger outbreaks. In all cases, whether a more or less infective strain will be introduced, or whether an outbreak will be large or small, is not known in advance, but traceability systems must be in place in advance to be useful.

Historically, in the US, the implementation of EIDs or radio frequency identification (RFID) tags has been proposed multiple times but has been met with producer resistance (16). This research supports the value and need for rapid and accurate traceability, and the most likely means to achieve it is through the use of EID technology. More economic research is needed to estimate the cost–benefit ratio of improved traceability during an outbreak. Clear economic value of traceability would support the adoption of EID technology. Further, an economic analysis may be warranted to assess the trade-offs associated with decreasing the radii of control areas and surveillance zones. While possibly useful when surveillance personnel are limited, the risk of detrimental increases in IPs and farms under surveillance during large outbreaks is concerning, so the practice cannot currently be recommended.

## 5 Conclusion

In the modeled scenario, tracing was most effective at reducing the likelihood of large outbreaks. This was especially evident in outbreaks with detection delayed to day 21 in the feedlot and dairy introductions. There was substantial variability in outbreak size and the magnitude of tracing impact depending on the day of detection. The impacts of reducing the size of control areas and surveillance zones were also highly variable and could be detrimental in large outbreaks. Ultimately, more research into the factors that lead to the variability and potential economic impacts of these control strategies is needed for animal health officials to make the most informed decision.

## Data Availability

The raw data supporting the conclusions of this article will be made available by the authors, without undue reservation.
