# Supplementary material for: Animal disease traceability: evaluation of simulated foot-and-mouth disease outbreak metrics with implementation of improved contact tracing of cattle
Source: Front Vet Sci. 2026 May 5;13:1804982. doi: 10.3389/fvets.2026.1804982 (PMC13196379; doi:10.3389/fvets.2026.1804982)
Supplement: Supplementary file 1 [file Data_Sheet_1.PDF]

Animal Disease Traceability: Evaluation of Simulated Foot-and-Mouth Disease Outbreak[1] Metrics With Implementation of Improved Contact Tracing

Supplement 1: Key Parameters

```
[Infectivity1]
TimePeriodStart=1
TimePeriodStop=90
InfectivityRelativeTo=infection
MaximumTimeOfInfectiousness=BetaPert 30 34 42
ResetInfection=Y
ResetInfectionDelay=BetaPert 150 180 210
MaximumTimeOfInfectiousness[bison|dealer_bison|cow_calf_l|cow_calf_s|dairy_l|dairy_s|dairy_heifer_calf_l|dairy_heifer_calf_m|dairy_he
ifer_calf_s|feedlot_cattle_l|feedlot_cattle_m|feedlot_cattle_s|stocker_l|stocker_s|dealer_cattle|goat_dairy|goat_other|dealer_goat|sh
eep_l|sheep_s|feedlot_sheep|dealer_sheep|transitional_swine|farrow_to_feeder_l|farrow_to_feeder_s|farrow_to_finish_l|farrow_to_finish
_s|farrow_to_wean_l|farrow_to_wean_s|grower_finisher_l|grower_finisher_s|nursery_l|nursery_s|other_swine_l|other_swine_s|dealer_swine
][]=BetaPert 30 34 42
MaximumTimeOfInfectiousness[livestock_market_bison_cattle|livestock_market_goat_sheep|livestock_market_swine|processor_bison|processo
r_cattle|processor_cattle_l|processor_goat|processor_sheep|processor_swine|processor_swine_l][]=BetaPert 7 10 14
ResetInfectionDelay[bison|dealer_bison|cow_calf_l|cow_calf_s|dairy_l|dairy_s|dairy_heifer_calf_l|dairy_heifer_calf_m|dairy_heifer_cal
f_s|feedlot_cattle_l|feedlot_cattle_m|feedlot_cattle_s|stocker_l|stocker_s|dealer_cattle|goat_dairy|goat_other|dealer_goat|sheep_l|sh
eep_s|feedlot_sheep|dealer_sheep|transitional_swine|farrow_to_feeder_l|farrow_to_feeder_s|farrow_to_finish_l|farrow_to_finish_s|farro
w_to_wean_l|farrow_to_wean_s|grower_finisher_l|grower_finisher_s|nursery_l|nursery_s|other_swine_l|other_swine_s|dealer_swine][]=Beta
Pert 150 180 210
ResetInfectionDelay[livestock_market_bison_cattle|livestock_market_goat_sheep|livestock_market_swine|processor_bison|processor_cattle
|processor_cattle_l|processor_goat|processor_sheep|processor_swine|processor_swine_l][]=Constant 1
TimeToClinicalSigns[bison]=Lookup 1,0.0014,0.0208,0.1257,0.4093,0.7927,1;0,0,1,2,3,4,5
TimeToClinicalSigns[cattle]=Lookup 1,0.0014,0.0208,0.1257,0.4093,0.7927,1;0,0,1,2,3,4,5
TimeToClinicalSigns[goat]=Lookup
1,0.0014,0.0104,0.0403,0.1058,0.2138,0.3561,0.5124,0.6596,0.7808,0.8695,0.9631,0.9921,0.9967,0.9987,0.9995,0.9998,0.9999,1;0,0,1,2,3,
4,5,6,7,8,9,10,11,12,13,14,15,16,17
TimeToClinicalSigns[sheep]=Lookup
1,0.0014,0.0104,0.0403,0.1058,0.2138,0.3561,0.5124,0.6596,0.7808,0.8695,0.9631,0.9921,0.9967,0.9987,0.9995,0.9998,0.9999,1;0,0,1,2,3,
4,5,6,7,8,9,10,11,12,13,14,15,16,17
TimeToClinicalSigns[sow]=Lookup 1,0,0.7347,0.9959,1;0,0,1,2,3
TimeToClinicalSigns[piglet]=Lookup 1,0,0.7347,0.9959,1;0,0,1,2,3
TimeToClinicalSigns[nursery]=Lookup 1,0,0.7347,0.9959,1;0,0,1,2,3
TimeToClinicalSigns[finisher]=Lookup 1,0,0.7347,0.9959,1;0,0,1,2,3
TimeToClinicalSigns[other_swine]=Lookup 1,0,0.7347,0.9959,1;0,0,1,2,3
Infectivity[][(in_atlantic_coast_climate_zone|in_northwest_climate_zone|in_west_climate_zone)&!(vaccinated_bison_cattle|vaccimmune_bi
son_cattle|vaccinated_swine_1|vaccinated_swine_2|vaccimmune_swine)][]=Constant Constant 1
Infectivity[][in_central_climate_zone&!(vaccinated_bison_cattle|vaccimmune_bison_cattle|vaccinated_swine_1|vaccinated_swine_2|vaccimm
une_swine)][]=Constant Constant 0.9
Infectivity[][in_new_england_climate_zone&!(vaccinated_bison_cattle|vaccimmune_bison_cattle|vaccinated_swine_1|vaccinated_swine_2|vac
cimmune_swine)][]=Constant Constant 0.85
Infectivity[][vaccinated_bison_cattle&!vaccimmune_bison_cattle][]=Constant Constant 0.05


[Infectivity2]
TimePeriodStart=91
TimePeriodStop=182
```

```
InfectivityRelativeTo=infection
MaximumTimeOfInfectiousness=BetaPert 30 34 42
ResetInfection=Y
ResetInfectionDelay=BetaPert 150 180 210
MaximumTimeOfInfectiousness[bison|dealer_bison|cow_calf_l|cow_calf_s|dairy_l|dairy_s|dairy_heifer_calf_l|dairy_heifer_calf_m|dairy_he
ifer_calf_s|feedlot_cattle_l|feedlot_cattle_m|feedlot_cattle_s|stocker_l|stocker_s|dealer_cattle|goat_dairy|goat_other|dealer_goat|sh
eep_l|sheep_s|feedlot_sheep|dealer_sheep|transitional_swine|farrow_to_feeder_l|farrow_to_feeder_s|farrow_to_finish_l|farrow_to_finish
_s|farrow_to_wean_l|farrow_to_wean_s|grower_finisher_l|grower_finisher_s|nursery_l|nursery_s|other_swine_l|other_swine_s|dealer_swine
][]=BetaPert 30 34 42
MaximumTimeOfInfectiousness[livestock_market_bison_cattle|livestock_market_goat_sheep|livestock_market_swine|processor_bison|processo
r_cattle|processor_cattle_l|processor_goat|processor_sheep|processor_swine|processor_swine_l][]=BetaPert 7 10 14
ResetInfectionDelay[bison|dealer_bison|cow_calf_l|cow_calf_s|dairy_l|dairy_s|dairy_heifer_calf_l|dairy_heifer_calf_m|dairy_heifer_cal
f_s|feedlot_cattle_l|feedlot_cattle_m|feedlot_cattle_s|stocker_l|stocker_s|dealer_cattle|goat_dairy|goat_other|dealer_goat|sheep_l|sh
eep_s|feedlot_sheep|dealer_sheep|transitional_swine|farrow_to_feeder_l|farrow_to_feeder_s|farrow_to_finish_l|farrow_to_finish_s|farro
w_to_wean_l|farrow_to_wean_s|grower_finisher_l|grower_finisher_s|nursery_l|nursery_s|other_swine_l|other_swine_s|dealer_swine][]=Beta
Pert 150 180 210
ResetInfectionDelay[livestock_market_bison_cattle|livestock_market_goat_sheep|livestock_market_swine|processor_bison|processor_cattle
|processor_cattle_l|processor_goat|processor_sheep|processor_swine|processor_swine_l][]=Constant 1
TimeToClinicalSigns[bison]=Lookup 1,0.0014,0.0208,0.1257,0.4093,0.7927,1;0,0,1,2,3,4,5
TimeToClinicalSigns[cattle]=Lookup 1,0.0014,0.0208,0.1257,0.4093,0.7927,1;0,0,1,2,3,4,5
TimeToClinicalSigns[goat]=Lookup
1,0.0014,0.0104,0.0403,0.1058,0.2138,0.3561,0.5124,0.6596,0.7808,0.8695,0.9631,0.9921,0.9967,0.9987,0.9995,0.9998,0.9999,1;0,0,1,2,3,
4,5,6,7,8,9,10,11,12,13,14,15,16,17
TimeToClinicalSigns[sheep]=Lookup
1,0.0014,0.0104,0.0403,0.1058,0.2138,0.3561,0.5124,0.6596,0.7808,0.8695,0.9631,0.9921,0.9967,0.9987,0.9995,0.9998,0.9999,1;0,0,1,2,3,
4,5,6,7,8,9,10,11,12,13,14,15,16,17
TimeToClinicalSigns[sow]=Lookup 1,0,0.7347,0.9959,1;0,0,1,2,3
TimeToClinicalSigns[piglet]=Lookup 1,0,0.7347,0.9959,1;0,0,1,2,3
TimeToClinicalSigns[nursery]=Lookup 1,0,0.7347,0.9959,1;0,0,1,2,3
TimeToClinicalSigns[finisher]=Lookup 1,0,0.7347,0.9959,1;0,0,1,2,3
TimeToClinicalSigns[other_swine]=Lookup 1,0,0.7347,0.9959,1;0,0,1,2,3
Infectivity[][(in_atlantic_coast_climate_zone|in_central_climate_zone|in_new_england_climate_zone|in_northwest_climate_zone|in_west_c
limate_zone)&!(vaccinated_bison_cattle|vaccimmune_bison_cattle|vaccinated_swine_1|vaccinated_swine_2|vaccimmune_swine)][]=Constant
Constant 1

[Infectivity3]
TimePeriodStart=183
TimePeriodStop=274
InfectivityRelativeTo=infection
MaximumTimeOfInfectiousness=BetaPert 30 34 42
ResetInfection=Y
ResetInfectionDelay=BetaPert 150 180 210
MaximumTimeOfInfectiousness[bison|dealer_bison|cow_calf_l|cow_calf_s|dairy_l|dairy_s|dairy_heifer_calf_l|dairy_heifer_calf_m|dairy_he
ifer_calf_s|feedlot_cattle_l|feedlot_cattle_m|feedlot_cattle_s|stocker_l|stocker_s|dealer_cattle|goat_dairy|goat_other|dealer_goat|sh
eep_l|sheep_s|feedlot_sheep|dealer_sheep|transitional_swine|farrow_to_feeder_l|farrow_to_feeder_s|farrow_to_finish_l|farrow_to_finish
_s|farrow_to_wean_l|farrow_to_wean_s|grower_finisher_l|grower_finisher_s|nursery_l|nursery_s|other_swine_l|other_swine_s|dealer_swine
][]=BetaPert 30 34 42
MaximumTimeOfInfectiousness[livestock_market_bison_cattle|livestock_market_goat_sheep|livestock_market_swine|processor_bison|processo
r_cattle|processor_cattle_l|processor_goat|processor_sheep|processor_swine|processor_swine_l][]=BetaPert 7 10 14
ResetInfectionDelay[bison|dealer_bison|cow_calf_l|cow_calf_s|dairy_l|dairy_s|dairy_heifer_calf_l|dairy_heifer_calf_m|dairy_heifer_cal
f_s|feedlot_cattle_l|feedlot_cattle_m|feedlot_cattle_s|stocker_l|stocker_s|dealer_cattle|goat_dairy|goat_other|dealer_goat|sheep_l|sh
eep_s|feedlot_sheep|dealer_sheep|transitional_swine|farrow_to_feeder_l|farrow_to_feeder_s|farrow_to_finish_l|farrow_to_finish_s|farro
```

```
w_to_wean_l|farrow_to_wean_s|grower_finisher_l|grower_finisher_s|nursery_l|nursery_s|other_swine_l|other_swine_s|dealer_swine][]=Beta
Pert 150 180 210
ResetInfectionDelay[livestock_market_bison_cattle|livestock_market_goat_sheep|livestock_market_swine|processor_bison|processor_cattle
|processor_cattle_l|processor_goat|processor_sheep|processor_swine|processor_swine_l][]=Constant 1
TimeToClinicalSigns[bison]=Lookup 1,0.0014,0.0208,0.1257,0.4093,0.7927,1;0,0,1,2,3,4,5
TimeToClinicalSigns[cattle]=Lookup 1,0.0014,0.0208,0.1257,0.4093,0.7927,1;0,0,1,2,3,4,5
TimeToClinicalSigns[goat]=Lookup
1,0.0014,0.0104,0.0403,0.1058,0.2138,0.3561,0.5124,0.6596,0.7808,0.8695,0.9631,0.9921,0.9967,0.9987,0.9995,0.9998,0.9999,1;0,0,1,2,3,
4,5,6,7,8,9,10,11,12,13,14,15,16,17
TimeToClinicalSigns[sheep]=Lookup
1,0.0014,0.0104,0.0403,0.1058,0.2138,0.3561,0.5124,0.6596,0.7808,0.8695,0.9631,0.9921,0.9967,0.9987,0.9995,0.9998,0.9999,1;0,0,1,2,3,
4,5,6,7,8,9,10,11,12,13,14,15,16,17
TimeToClinicalSigns[sow]=Lookup 1,0,0.7347,0.9959,1;0,0,1,2,3
TimeToClinicalSigns[piglet]=Lookup 1,0,0.7347,0.9959,1;0,0,1,2,3
TimeToClinicalSigns[nursery]=Lookup 1,0,0.7347,0.9959,1;0,0,1,2,3
TimeToClinicalSigns[finisher]=Lookup 1,0,0.7347,0.9959,1;0,0,1,2,3
TimeToClinicalSigns[other_swine]=Lookup 1,0,0.7347,0.9959,1;0,0,1,2,3
Infectivity[][in_atlantic_coast_climate_zone&!(vaccinated_bison_cattle|vaccimmune_bison_cattle|vaccinated_swine_1|vaccinated_swine_2|
vaccimmune_swine)][]=Constant Constant 0.9
Infectivity[][(in_central_climate_zone|in_west_climate_zone)&!(vaccinated_bison_cattle|vaccimmune_bison_cattle|vaccinated_swine_1|vac
cinated_swine_2|vaccimmune_swine)][]=Constant Constant 0.85
Infectivity[][(in_new_england_climate_zone|in_northwest_climate_zone)&!(vaccinated_bison_cattle|vaccimmune_bison_cattle|vaccinated_sw
ine_1|vaccinated_swine_2|vaccimmune_swine)][]=Constant Constant 0.95

[Infectivity4]
TimePeriodStart=275
TimePeriodStop=365
InfectivityRelativeTo=infection
MaximumTimeOfInfectiousness=BetaPert 30 34 42
ResetInfection=Y
ResetInfectionDelay=BetaPert 150 180 210
MaximumTimeOfInfectiousness[livestock_market_bison_cattle|livestock_market_goat_sheep|livestock_market_swine][]=BetaPert 7 10 14
MaximumTimeOfInfectiousness[bison|dealer_bison|cow_calf_l|cow_calf_s|dairy_l|dairy_s|dairy_heifer_calf_l|dairy_heifer_calf_m|dairy_he
ifer_calf_s|feedlot_cattle_l|feedlot_cattle_m|feedlot_cattle_s|stocker_l|stocker_s|dealer_cattle|goat_dairy|goat_other|dealer_goat|sh
eep_l|sheep_s|feedlot_sheep|dealer_sheep|transitional_swine|farrow_to_feeder_l|farrow_to_feeder_s|farrow_to_finish_l|farrow_to_finish
_s|farrow_to_wean_l|farrow_to_wean_s|grower_finisher_l|grower_finisher_s|nursery_l|nursery_s|other_swine_l|other_swine_s|dealer_swine
][]=BetaPert 30 34 42
ResetInfectionDelay[bison|dealer_bison|cow_calf_l|cow_calf_s|dairy_l|dairy_s|dairy_heifer_calf_l|dairy_heifer_calf_m|dairy_heifer_cal
f_s|feedlot_cattle_l|feedlot_cattle_m|feedlot_cattle_s|stocker_l|stocker_s|dealer_cattle|goat_dairy|goat_other|dealer_goat|sheep_l|sh
eep_s|feedlot_sheep|dealer_sheep|transitional_swine|farrow_to_feeder_l|farrow_to_feeder_s|farrow_to_finish_l|farrow_to_finish_s|farro
w_to_wean_l|farrow_to_wean_s|grower_finisher_l|grower_finisher_s|nursery_l|nursery_s|other_swine_l|other_swine_s|dealer_swine][]=Beta
Pert 150 180 210
ResetInfectionDelay[livestock_market_bison_cattle|livestock_market_goat_sheep|livestock_market_swine|processor_bison|processor_cattle
|processor_cattle_l|processor_goat|processor_sheep|processor_swine|processor_swine_l][]=Constant 1
TimeToClinicalSigns[bison]=Lookup 1,0.0014,0.0208,0.1257,0.4093,0.7927,1;0,0,1,2,3,4,5
TimeToClinicalSigns[cattle]=Lookup 1,0.0014,0.0208,0.1257,0.4093,0.7927,1;0,0,1,2,3,4,5
TimeToClinicalSigns[goat]=Lookup
1,0.0014,0.0104,0.0403,0.1058,0.2138,0.3561,0.5124,0.6596,0.7808,0.8695,0.9631,0.9921,0.9967,0.9987,0.9995,0.9998,0.9999,1;0,0,1,2,3,
4,5,6,7,8,9,10,11,12,13,14,15,16,17
TimeToClinicalSigns[sheep]=Lookup
1,0.0014,0.0104,0.0403,0.1058,0.2138,0.3561,0.5124,0.6596,0.7808,0.8695,0.9631,0.9921,0.9967,0.9987,0.9995,0.9998,0.9999,1;0,0,1,2,3,
4,5,6,7,8,9,10,11,12,13,14,15,16,17
```

```
TimeToClinicalSigns[sow]=Lookup 1,0,0.7347,0.9959,1;0,0,1,2,3
TimeToClinicalSigns[piglet]=Lookup 1,0,0.7347,0.9959,1;0,0,1,2,3
TimeToClinicalSigns[nursery]=Lookup 1,0,0.7347,0.9959,1;0,0,1,2,3
TimeToClinicalSigns[finisher]=Lookup 1,0,0.7347,0.9959,1;0,0,1,2,3
TimeToClinicalSigns[other_swine]=Lookup 1,0,0.7347,0.9959,1;0,0,1,2,3
Infectivity[][(in_atlantic_coast_climate_zone|in_central_climate_zone|in_new_england_climate_zone|in_northwest_climate_zone)&!(vaccin
ated_bison_cattle|vaccimmune_bison_cattle|vaccinated_swine_1|vaccinated_swine_2|vaccimmune_swine)][]=Constant Constant 1
Infectivity[][in_west_climate_zone&!(vaccinated_bison_cattle|vaccimmune_bison_cattle|vaccinated_swine_1|vaccinated_swine_2|vaccimmune
_swine)][]=Constant Constant 0.95

[Infectivity5]
TimePeriodStart=366
TimePeriodStop=455
InfectivityRelativeTo=infection
MaximumTimeOfInfectiousness=BetaPert 30 34 42
ResetInfection=Y
ResetInfectionDelay=BetaPert 150 180 210
MaximumTimeOfInfectiousness[bison|dealer_bison|cow_calf_l|cow_calf_s|dairy_l|dairy_s|dairy_heifer_calf_l|dairy_heifer_calf_m|dairy_he
ifer_calf_s|feedlot_cattle_l|feedlot_cattle_m|feedlot_cattle_s|stocker_l|stocker_s|dealer_cattle|goat_dairy|goat_other|dealer_goat|sh
eep_l|sheep_s|feedlot_sheep|dealer_sheep|transitional_swine|farrow_to_feeder_l|farrow_to_feeder_s|farrow_to_finish_l|farrow_to_finish
_s|farrow_to_wean_l|farrow_to_wean_s|grower_finisher_l|grower_finisher_s|nursery_l|nursery_s|other_swine_l|other_swine_s|dealer_swine
][]=BetaPert 30 34 42
MaximumTimeOfInfectiousness[livestock_market_bison_cattle|livestock_market_goat_sheep|livestock_market_swine|processor_bison|processo
r_cattle|processor_cattle_l|processor_goat|processor_sheep|processor_swine|processor_swine_l][]=BetaPert 7 10 14
ResetInfectionDelay[bison|dealer_bison|cow_calf_l|cow_calf_s|dairy_l|dairy_s|dairy_heifer_calf_l|dairy_heifer_calf_m|dairy_heifer_cal
f_s|feedlot_cattle_l|feedlot_cattle_m|feedlot_cattle_s|stocker_l|stocker_s|dealer_cattle|goat_dairy|goat_other|dealer_goat|sheep_l|sh
eep_s|feedlot_sheep|dealer_sheep|transitional_swine|farrow_to_feeder_l|farrow_to_feeder_s|farrow_to_finish_l|farrow_to_finish_s|farro
w_to_wean_l|farrow_to_wean_s|grower_finisher_l|grower_finisher_s|nursery_l|nursery_s|other_swine_l|other_swine_s|dealer_swine][]=Beta
Pert 150 180 210
ResetInfectionDelay[livestock_market_bison_cattle|livestock_market_goat_sheep|livestock_market_swine|processor_bison|processor_cattle
|processor_cattle_l|processor_goat|processor_sheep|processor_swine|processor_swine_l][]=Constant 1
TimeToClinicalSigns[bison]=Lookup 1,0.0014,0.0208,0.1257,0.4093,0.7927,1;0,0,1,2,3,4,5
TimeToClinicalSigns[cattle]=Lookup 1,0.0014,0.0208,0.1257,0.4093,0.7927,1;0,0,1,2,3,4,5
TimeToClinicalSigns[goat]=Lookup
1,0.0014,0.0104,0.0403,0.1058,0.2138,0.3561,0.5124,0.6596,0.7808,0.8695,0.9631,0.9921,0.9967,0.9987,0.9995,0.9998,0.9999,1;0,0,1,2,3,
4,5,6,7,8,9,10,11,12,13,14,15,16,17
TimeToClinicalSigns[sheep]=Lookup
1,0.0014,0.0104,0.0403,0.1058,0.2138,0.3561,0.5124,0.6596,0.7808,0.8695,0.9631,0.9921,0.9967,0.9987,0.9995,0.9998,0.9999,1;0,0,1,2,3,
4,5,6,7,8,9,10,11,12,13,14,15,16,17
TimeToClinicalSigns[sow]=Lookup 1,0,0.7347,0.9959,1;0,0,1,2,3
TimeToClinicalSigns[piglet]=Lookup 1,0,0.7347,0.9959,1;0,0,1,2,3
TimeToClinicalSigns[nursery]=Lookup 1,0,0.7347,0.9959,1;0,0,1,2,3
TimeToClinicalSigns[finisher]=Lookup 1,0,0.7347,0.9959,1;0,0,1,2,3
TimeToClinicalSigns[other_swine]=Lookup 1,0,0.7347,0.9959,1;0,0,1,2,3
Infectivity[][(in_atlantic_coast_climate_zone|in_northwest_climate_zone|in_west_climate_zone)&!(vaccinated_bison_cattle|vaccimmune_bi
son_cattle|vaccinated_swine_1|vaccinated_swine_2|vaccimmune_swine)][]=Constant Constant 1
Infectivity[][in_central_climate_zone&!(vaccinated_bison_cattle|vaccimmune_bison_cattle|vaccinated_swine_1|vaccinated_swine_2|vaccimm
une_swine)][]=Constant Constant 0.9
Infectivity[][in_new_england_climate_zone&!(vaccinated_bison_cattle|vaccimmune_bison_cattle|vaccinated_swine_1|vaccinated_swine_2|vac
cimmune_swine)][]=Constant Constant 0.85

[Infectivity6]
```

```
TimePeriodStart=456
TimePeriodStop=547
InfectivityRelativeTo=infection
MaximumTimeOfInfectiousness=BetaPert 30 34 42
ResetInfection=Y
ResetInfectionDelay=BetaPert 150 180 210
MaximumTimeOfInfectiousness[bison|dealer_bison|cow_calf_l|cow_calf_s|dairy_l|dairy_s|dairy_heifer_calf_l|dairy_heifer_calf_m|dairy_he
ifer_calf_s|feedlot_cattle_l|feedlot_cattle_m|feedlot_cattle_s|stocker_l|stocker_s|dealer_cattle|goat_dairy|goat_other|dealer_goat|sh
eep_l|sheep_s|feedlot_sheep|dealer_sheep|transitional_swine|farrow_to_feeder_l|farrow_to_feeder_s|farrow_to_finish_l|farrow_to_finish
_s|farrow_to_wean_l|farrow_to_wean_s|grower_finisher_l|grower_finisher_s|nursery_l|nursery_s|other_swine_l|other_swine_s|dealer_swine
][]=BetaPert 30 34 42
MaximumTimeOfInfectiousness[livestock_market_bison_cattle|livestock_market_goat_sheep|livestock_market_swine|processor_bison|processo
r_cattle|processor_cattle_l|processor_goat|processor_sheep|processor_swine|processor_swine_l][]=BetaPert 7 10 14
ResetInfectionDelay[bison|dealer_bison|cow_calf_l|cow_calf_s|dairy_l|dairy_s|dairy_heifer_calf_l|dairy_heifer_calf_m|dairy_heifer_cal
f_s|feedlot_cattle_l|feedlot_cattle_m|feedlot_cattle_s|stocker_l|stocker_s|dealer_cattle|goat_dairy|goat_other|dealer_goat|sheep_l|sh
eep_s|feedlot_sheep|dealer_sheep|transitional_swine|farrow_to_feeder_l|farrow_to_feeder_s|farrow_to_finish_l|farrow_to_finish_s|farro
w_to_wean_l|farrow_to_wean_s|grower_finisher_l|grower_finisher_s|nursery_l|nursery_s|other_swine_l|other_swine_s|dealer_swine][]=Beta
Pert 150 180 210
ResetInfectionDelay[livestock_market_bison_cattle|livestock_market_goat_sheep|livestock_market_swine|processor_bison|processor_cattle
|processor_cattle_l|processor_goat|processor_sheep|processor_swine|processor_swine_l][]=Constant 1
TimeToClinicalSigns[bison]=Lookup 1,0.0014,0.0208,0.1257,0.4093,0.7927,1;0,0,1,2,3,4,5
TimeToClinicalSigns[cattle]=Lookup 1,0.0014,0.0208,0.1257,0.4093,0.7927,1;0,0,1,2,3,4,5
TimeToClinicalSigns[goat]=Lookup
1,0.0014,0.0104,0.0403,0.1058,0.2138,0.3561,0.5124,0.6596,0.7808,0.8695,0.9631,0.9921,0.9967,0.9987,0.9995,0.9998,0.9999,1;0,0,1,2,3,
4,5,6,7,8,9,10,11,12,13,14,15,16,17
TimeToClinicalSigns[sheep]=Lookup
1,0.0014,0.0104,0.0403,0.1058,0.2138,0.3561,0.5124,0.6596,0.7808,0.8695,0.9631,0.9921,0.9967,0.9987,0.9995,0.9998,0.9999,1;0,0,1,2,3,
4,5,6,7,8,9,10,11,12,13,14,15,16,17
TimeToClinicalSigns[sow]=Lookup 1,0,0.7347,0.9959,1;0,0,1,2,3
TimeToClinicalSigns[piglet]=Lookup 1,0,0.7347,0.9959,1;0,0,1,2,3
TimeToClinicalSigns[nursery]=Lookup 1,0,0.7347,0.9959,1;0,0,1,2,3
TimeToClinicalSigns[finisher]=Lookup 1,0,0.7347,0.9959,1;0,0,1,2,3
TimeToClinicalSigns[other_swine]=Lookup 1,0,0.7347,0.9959,1;0,0,1,2,3
Infectivity[][(in_atlantic_coast_climate_zone|in_central_climate_zone|in_new_england_climate_zone|in_northwest_climate_zone|in_west_c
limate_zone)&!(vaccinated_bison_cattle|vaccimmune_bison_cattle|vaccinated_swine_1|vaccinated_swine_2|vaccimmune_swine)][]=Constant
Constant 1

[Infectivity7]
TimePeriodStart=548
TimePeriodStop=639
InfectivityRelativeTo=infection
MaximumTimeOfInfectiousness=BetaPert 30 34 42
ResetInfection=Y
ResetInfectionDelay=BetaPert 150 180 210
MaximumTimeOfInfectiousness[bison|dealer_bison|cow_calf_l|cow_calf_s|dairy_l|dairy_s|dairy_heifer_calf_l|dairy_heifer_calf_m|dairy_he
ifer_calf_s|feedlot_cattle_l|feedlot_cattle_m|feedlot_cattle_s|stocker_l|stocker_s|dealer_cattle|goat_dairy|goat_other|dealer_goat|sh
eep_l|sheep_s|feedlot_sheep|dealer_sheep|transitional_swine|farrow_to_feeder_l|farrow_to_feeder_s|farrow_to_finish_l|farrow_to_finish
_s|farrow_to_wean_l|farrow_to_wean_s|grower_finisher_l|grower_finisher_s|nursery_l|nursery_s|other_swine_l|other_swine_s|dealer_swine
][]=BetaPert 30 34 42
MaximumTimeOfInfectiousness[livestock_market_bison_cattle|livestock_market_goat_sheep|livestock_market_swine|processor_bison|processo
r_cattle|processor_cattle_l|processor_goat|processor_sheep|processor_swine|processor_swine_l][]=BetaPert 7 10 14
```

```
ResetInfectionDelay[bison|dealer_bison|cow_calf_l|cow_calf_s|dairy_l|dairy_s|dairy_heifer_calf_l|dairy_heifer_calf_m|dairy_heifer_cal
f_s|feedlot_cattle_l|feedlot_cattle_m|feedlot_cattle_s|stocker_l|stocker_s|dealer_cattle|goat_dairy|goat_other|dealer_goat|sheep_l|sh
eep_s|feedlot_sheep|dealer_sheep|transitional_swine|farrow_to_feeder_l|farrow_to_feeder_s|farrow_to_finish_l|farrow_to_finish_s|farro
w_to_wean_l|farrow_to_wean_s|grower_finisher_l|grower_finisher_s|nursery_l|nursery_s|other_swine_l|other_swine_s|dealer_swine][]=Beta
Pert 150 180 210
ResetInfectionDelay[livestock_market_bison_cattle|livestock_market_goat_sheep|livestock_market_swine|processor_bison|processor_cattle
|processor_cattle_l|processor_goat|processor_sheep|processor_swine|processor_swine_l][]=Constant 1
TimeToClinicalSigns[bison]=Lookup 1,0.0014,0.0208,0.1257,0.4093,0.7927,1;0,0,1,2,3,4,5
TimeToClinicalSigns[cattle]=Lookup 1,0.0014,0.0208,0.1257,0.4093,0.7927,1;0,0,1,2,3,4,5
TimeToClinicalSigns[goat]=Lookup
1,0.0014,0.0104,0.0403,0.1058,0.2138,0.3561,0.5124,0.6596,0.7808,0.8695,0.9631,0.9921,0.9967,0.9987,0.9995,0.9998,0.9999,1;0,0,1,2,3,
4,5,6,7,8,9,10,11,12,13,14,15,16,17
TimeToClinicalSigns[sheep]=Lookup
1,0.0014,0.0104,0.0403,0.1058,0.2138,0.3561,0.5124,0.6596,0.7808,0.8695,0.9631,0.9921,0.9967,0.9987,0.9995,0.9998,0.9999,1;0,0,1,2,3,
4,5,6,7,8,9,10,11,12,13,14,15,16,17
TimeToClinicalSigns[sow]=Lookup 1,0,0.7347,0.9959,1;0,0,1,2,3
TimeToClinicalSigns[piglet]=Lookup 1,0,0.7347,0.9959,1;0,0,1,2,3
TimeToClinicalSigns[nursery]=Lookup 1,0,0.7347,0.9959,1;0,0,1,2,3
TimeToClinicalSigns[finisher]=Lookup 1,0,0.7347,0.9959,1;0,0,1,2,3
TimeToClinicalSigns[other_swine]=Lookup 1,0,0.7347,0.9959,1;0,0,1,2,3
Infectivity[][in_atlantic_coast_climate_zone&!(vaccinated_bison_cattle|vaccimmune_bison_cattle|vaccinated_swine_1|vaccinated_swine_2|
vaccimmune_swine)][]=Constant Constant 0.9
Infectivity[][(in_central_climate_zone|in_west_climate_zone)&!(vaccinated_bison_cattle|vaccimmune_bison_cattle|vaccinated_swine_1|vac
cinated_swine_2|vaccimmune_swine)][]=Constant Constant 0.85
Infectivity[][(in_new_england_climate_zone|in_northwest_climate_zone)&!(vaccinated_bison_cattle|vaccimmune_bison_cattle|vaccinated_sw
ine_1|vaccinated_swine_2|vaccimmune_swine)][]=Constant Constant 0.95

[Infectivity8]
TimePeriodStart=640
TimePeriodStop=730
InfectivityRelativeTo=infection
MaximumTimeOfInfectiousness=BetaPert 30 34 42
ResetInfection=Y
ResetInfectionDelay=BetaPert 150 180 210
MaximumTimeOfInfectiousness[livestock_market_bison_cattle|livestock_market_goat_sheep|livestock_market_swine][]=BetaPert 7 10 14
MaximumTimeOfInfectiousness[bison|dealer_bison|cow_calf_l|cow_calf_s|dairy_l|dairy_s|dairy_heifer_calf_l|dairy_heifer_calf_m|dairy_he
ifer_calf_s|feedlot_cattle_l|feedlot_cattle_m|feedlot_cattle_s|stocker_l|stocker_s|dealer_cattle|goat_dairy|goat_other|dealer_goat|sh
eep_l|sheep_s|feedlot_sheep|dealer_sheep|transitional_swine|farrow_to_feeder_l|farrow_to_feeder_s|farrow_to_finish_l|farrow_to_finish
_s|farrow_to_wean_l|farrow_to_wean_s|grower_finisher_l|grower_finisher_s|nursery_l|nursery_s|other_swine_l|other_swine_s|dealer_swine
][]=BetaPert 30 34 42
ResetInfectionDelay[bison|dealer_bison|cow_calf_l|cow_calf_s|dairy_l|dairy_s|dairy_heifer_calf_l|dairy_heifer_calf_m|dairy_heifer_cal
f_s|feedlot_cattle_l|feedlot_cattle_m|feedlot_cattle_s|stocker_l|stocker_s|dealer_cattle|goat_dairy|goat_other|dealer_goat|sheep_l|sh
eep_s|feedlot_sheep|dealer_sheep|transitional_swine|farrow_to_feeder_l|farrow_to_feeder_s|farrow_to_finish_l|farrow_to_finish_s|farro
w_to_wean_l|farrow_to_wean_s|grower_finisher_l|grower_finisher_s|nursery_l|nursery_s|other_swine_l|other_swine_s|dealer_swine][]=Beta
Pert 150 180 210
ResetInfectionDelay[livestock_market_bison_cattle|livestock_market_goat_sheep|livestock_market_swine|processor_bison|processor_cattle
|processor_cattle_l|processor_goat|processor_sheep|processor_swine|processor_swine_l][]=Constant 1
TimeToClinicalSigns[bison]=Lookup 1,0.0014,0.0208,0.1257,0.4093,0.7927,1;0,0,1,2,3,4,5
TimeToClinicalSigns[cattle]=Lookup 1,0.0014,0.0208,0.1257,0.4093,0.7927,1;0,0,1,2,3,4,5
TimeToClinicalSigns[goat]=Lookup
1,0.0014,0.0104,0.0403,0.1058,0.2138,0.3561,0.5124,0.6596,0.7808,0.8695,0.9631,0.9921,0.9967,0.9987,0.9995,0.9998,0.9999,1;0,0,1,2,3,
4,5,6,7,8,9,10,11,12,13,14,15,16,17
```

```
TimeToClinicalSigns[sheep]=Lookup
1,0.0014,0.0104,0.0403,0.1058,0.2138,0.3561,0.5124,0.6596,0.7808,0.8695,0.9631,0.9921,0.9967,0.9987,0.9995,0.9998,0.9999,1;0,0,1,2,3,
4,5,6,7,8,9,10,11,12,13,14,15,16,17
TimeToClinicalSigns[sow]=Lookup 1,0,0.7347,0.9959,1;0,0,1,2,3
TimeToClinicalSigns[piglet]=Lookup 1,0,0.7347,0.9959,1;0,0,1,2,3
TimeToClinicalSigns[nursery]=Lookup 1,0,0.7347,0.9959,1;0,0,1,2,3
TimeToClinicalSigns[finisher]=Lookup 1,0,0.7347,0.9959,1;0,0,1,2,3
TimeToClinicalSigns[other_swine]=Lookup 1,0,0.7347,0.9959,1;0,0,1,2,3
Infectivity[][(in_atlantic_coast_climate_zone|in_central_climate_zone|in_new_england_climate_zone|in_northwest_climate_zone)&!(vaccin
ated_bison_cattle|vaccimmune_bison_cattle|vaccinated_swine_1|vaccinated_swine_2|vaccimmune_swine)][]=Constant Constant 1
Infectivity[][in_west_climate_zone&!(vaccinated_bison_cattle|vaccimmune_bison_cattle|vaccinated_swine_1|vaccinated_swine_2|vaccimmune
_swine)][]=Constant Constant 0.95



[Depopulation1]
ControlName=depopulation_large_cattle
ActivationOption=detected_farm
FarmSelectionOption=detected_farm
FarmClasses=cow_calf_l | dairy_heifer_calf_l|feedlot_cattle_s|stocker_l|sheep_l|processor_cattle_l
ActionResource=resource_depopulation_large_cattle
CompletedFarmState=depopulated

[Depopulation2]
ControlName=depopulation_small_cattle_other
ActivationOption=detected_farm
FarmSelectionOption=detected_farm
FarmClasses=bison | livestock_market_bison_cattle | dealer_bison | cow_calf_s | dairy_s | dairy_heifer_calf_m | dairy_heifer_calf_s |
stocker_s | dealer_cattle | goat_dairy | goat_other | livestock_market_goat_sheep | dealer_goat | sheep_s | dealer_sheep |
livestock_market_swine|processor_bison|processor_cattle|processor_goat|processor_sheep
ActionResource=resource_depopulation_small_cattle_other
CompletedFarmState=depopulated

[Depopulation3]
ControlName=depopulation_large_feedlots
ActivationOption=detected_farm
FarmSelectionOption=detected_farm
FarmClasses=feedlot_cattle_l
ActionResource=resource_depopulation_large_feedlots
CompletedFarmState=depopulated

[Depopulation4]
ControlName=depopulation_medium_feedlots
ActivationOption=detected_farm
FarmSelectionOption=detected_farm
FarmClasses=dairy_l|feedlot_cattle_m|feedlot_sheep
ActionResource=resource_depopulation_medium_feedlots
CompletedFarmState=depopulated

[Depopulation5]
ControlName=depopulation_large_swine
ActivationOption=detected_farm
```

```
FarmSelectionOption=detected_farm
FarmClasses=farrow_to_feeder_l | farrow_to_finish_l | farrow_to_wean_l | grower_finisher_l | nursery_l |
other_swine_l|processor_swine_l
ActionResource=resource_depopulation_large_swine
CompletedFarmState=depopulated

[Depopulation6]
ControlName=depopulation_small_swine
ActivationOption=detected_farm
FarmSelectionOption=detected_farm
FarmClasses=transitional_swine | farrow_to_feeder_s | farrow_to_finish_s | farrow_to_wean_s | grower_finisher_s | nursery_s |
other_swine_s| dealer_swine|processor_swine
ActionResource=resource_depopulation_small_swine
CompletedFarmState=depopulated

[Depopulation7]
ControlName=post_depopulation_spread
ActivationOption=time_period
TimePeriodTrigger=first_detection
FarmSelectionOption=zone
SelectionZone=passive_surveillance_post_detection_zone
FarmStates=depopulated
ActionResource=resource_post_depopulation
DelayedFarmState=post_depopulation_spread_completed
TimePeriodToDelayedState=3
RemoveDetectedFarms=N



[Surveillance1]
ControlName=passive_surveillance_post_detection
ActivationOption=time_period
TimePeriodTrigger=first_detection
SelectionZone=passive_surveillance_post_detection_zone
SelectionProbability=1.0
VisitDelay=Constant 0
VisitFrequency=BetaPert 1 1 2
VisitDuration=Constant 730
DelayToDetection=BetaPert 2 2 3
DetectionRelativeTo=Clinical_signs
DetectionProbability[bison][in_passive_surveillance_outbreak_phase&clinical_signs][]=Table
1,0,1,2,3,4,14;0,0.164,0.223,0.282,0.341,0.4,0.4
DetectionProbability[dealer_bison|dealer_cattle][in_passive_surveillance_outbreak_phase&clinical_signs][]=Table 1,0,14;0,0.2,0.2
DetectionProbability[cow_calf_l|dairy_heifer_calf_l|dairy_heifer_calf_m][in_passive_surveillance_outbreak_phase&clinical_sign s][]=Tab
le 1,0,1,2,3,4,5,6,7,8,14;0,0.024,0.1635,0.303,0.4425,0.582,0.5865,0.591,0.5955,0.6,0.6
DetectionProbability[cow_calf_s|dairy_heifer_calf_s][in_passive_surveillance_outbreak_phase&clinical_signs][]=Table
1,0,1,2,3,4,14;0,0.246,0.3345,0.423,0.5115,0.6,0.6
DetectionProbability[dairy_l][in_passive_surveillance_outbreak_phase&clinical_signs][]=Table
1,0,1,2,3,4,14;0,0.045,0.459,0.873,0.8865,0.9,0.9
DetectionProbability[dairy_s][in_passive_surveillance_outbreak_phase&clinical_signs][]=Table
1,0,1,2,3,4,14;0,0.0063,0.4262,0.846,0.873,0.9,0.9
```

```
DetectionProbability[feedlot_cattle_l|feedlot_cattle_m|feedlot_cattle_s][in_passive_surveillance_outbreak_phase&clinical_signs][]=Table 1,0,1,2,14;0,0.136,0.468,0.8,0.8
DetectionProbability[stocker_l|stocker_s][in_passive_surveillance_outbreak_phase&clinical_signs][]=Table 1,0,1,2,3,4,14;0,0.08,0.185,0.29,0.395,0.5,0.5
DetectionProbability[goat_dairy|goat_other|sheep_l|sheep_s][in_passive_surveillance_outbreak_phase&clinical_signs][]=Table 1,0,1,2,3,4,5,6,7,8,9,10,11,12,14;0,0.015,0.087,0.159,0.174,0.189,0.177,0.165,0.1395,0.114,0.0885,0.063,0.0315,0,0
DetectionProbability[feedlot_sheep][in_passive_surveillance_outbreak_phase&clinical_signs][]=Table 1,0,1,2,3,4,5,6,7,8,9,10,11,12,14;0,0.02,0.116,0.212,0.232,0.252,0.236,0.22,0.186,0.152,0.118,0.084,0.042,0,0
DetectionProbability[dealer_goat|dealer_sheep][in_passive_surveillance_outbreak_phase&clinical_signs][]=Table 1,0,1,2,3,4,5,6,7,8,9,10,11,12,14;0,0.0125,0.0725,0.1325,0.145,0.1575,0.1475,0.1375,0.11625,0.095,0.07375,0.0525,0.02625,0,0
DetectionProbability[transitional_swine|farrow_to_feeder_s|farrow_to_wean_s|farrow_to_finish_s|grower_finisher_s|nursery_s|other_swine_s][in_passive_surveillance_outbreak_phase&clinical_signs][]=Table 1,0,14;0,0.9,0.9
DetectionProbability[farrow_to_feeder_l|farrow_to_finish_l|farrow_to_wean_l|grower_finisher_l|nursery_l|other_swine_l][in_passive_surveillance_outbreak_phase&clinical_signs][]=Table 1,0,1,2,14;0,0.783,0.8415,0.9,0.9
DetectionProbability[dealer_swine][in_passive_surveillance_outbreak_phase&clinical_signs][]=Table 1,0,14;0,0.25,0.25
DetectionProbability[livestock_market_bison_cattle][in_passive_surveillance_outbreak_phase&clinical_signs][]=Table 1,0,1,2,3,4,14;0,0.08,0.185,0.29,0.395,0.5,0.5
DetectionProbability[livestock_market_goat_sheep][in_passive_surveillance_outbreak_phase&clinical_signs][]=Table 1,0,1,2,3,4,5,6,7,8,9,10,11,12,14;0,0.015,0.087,0.159,0.174,0.189,0.177,0.165,0.1395,0.114,0.0885,0.063,0.0315,0,0
DetectionProbability[livestock_market_swine][in_passive_surveillance_outbreak_phase&clinical_signs][]=Table 1,0,14;0,0.9,0.9

[Surveillance2]
ControlName=tracing_livestock_movements
ActivationOption=tracing
TimePeriodTrigger=first_detection
SurveillanceFarmState=direct_contact_tracing
SelectionProbability=1.0
VisitDelay=BetaPert 0 1 2
VisitFrequency=Constant 5
VisitDuration=Constant 28
DelayToDetection=BetaPert 0 1 2
DetectionRelativeTo=Clinical_signs
DetectionProbability[][][bison]=Table 1,0,1,43;-1,0.7275,0.97,0.97
DetectionProbability[][][cattle]=Table 1,0,1,43;-1,0.7275,0.97,0.97
DetectionProbability[][][goat]=Table 1,0,1,43;-1,0.7275,0.97,0.97
DetectionProbability[][][sheep]=Table 1,0,1,43;-1,0.7275,0.97,0.97
DetectionProbability[][][sow]=Table 1,0,1,42;1,0.97,0.97,0.97
DetectionProbability[][][piglet]=Table 1,0,1,42;1,0.97,0.97,0.97
DetectionProbability[][][nursery]=Table 1,0,1,42;1,0.97,0.97,0.97
DetectionProbability[][][finisher]=Table 1,0,1,42;1,0.97,0.97,0.97
DetectionProbability[][][other_swine]=Table 1,0,1,42;1,0.97,0.97,0.97

[Surveillance3]
ControlName=tracing_indirect_contacts
ActivationOption=tracing
TimePeriodTrigger=first_detection
SurveillanceFarmState=indirect_contact_tracing
SelectionProbability=1.0
VisitDelay=BetaPert 0 2 6
VisitFrequency=Constant 5
VisitDuration=Constant 28
DelayToDetection=BetaPert 0 1 2
```

```
DetectionRelativeTo=Clinical_signs
DetectionProbability[][][bison]=Table 1,0,1,43;-1,0.7275,0.97,0.97
DetectionProbability[][][cattle]=Table 1,0,1,43;-1,0.7275,0.97,0.97
DetectionProbability[][][goat]=Table 1,0,1,43;-1,0.7275,0.97,0.97
DetectionProbability[][][sheep]=Table 1,0,1,43;-1,0.7275,0.97,0.97
DetectionProbability[][][sow]=Table 1,0,1,42;1,0.97,0.97,0.97
DetectionProbability[][][piglet]=Table 1,0,1,42;1,0.97,0.97,0.97
DetectionProbability[][][nursery]=Table 1,0,1,42;1,0.97,0.97,0.97
DetectionProbability[][][finisher]=Table 1,0,1,42;1,0.97,0.97,0.97
DetectionProbability[][][other_swine]=Table 1,0,1,42;1,0.97,0.97,0.97

[Surveillance4]
ControlName=processors_detection_multiple_species
ActivationOption=detected_farm
TimePeriodTrigger=first_detection
SurveillanceFarmState=in_0m_to_5m_detection_zone
SelectionZone=0m_to_5m_detection_zone
SelectionProbability=1.0
VisitDelay=Constant 0
VisitFrequency=Constant 1
VisitDuration=Constant 1
DelayToDetection=Constant 0
DetectionRelativeTo=Infection
DetectionProbability[processor_bison|processor_cattle|processor_cattle_l|processor_goat|processor_sheep|processor_swine|processor_swi
ne_l][][]=Constant 1
DetectionProbability[bison|dealer_bison|livestock_market_bison_cattle|cow_calf_l|cow_calf_s|dairy_l|dairy_s|dairy_heifer_calf_l|dairy
_heifer_calf_m|dairy_heifer_calf_s|feedlot_cattle_l|feedlot_cattle_m|feedlot_cattle_s|stocker_l|stocker_s|dealer_cattle|goat_dairy|go
at_other|livestock_market_goat_sheep|dealer_goat|sheep_l|sheep_s|feedlot_sheep|dealer_sheep|transitional_swine|farrow_to_feeder_l|far
row_to_feeder_s|farrow_to_finish_l|farrow_to_finish_s|farrow_to_wean_l|farrow_to_wean_s|grower_finisher_l|grower_finisher_s|nursery_l
|nursery_s|other_swine_l|other_swine_s|livestock_market_swine|dealer_swine][][]=Constant 0

[Surveillance5]
ControlName=active_surveillance_processors
ActivationOption=time_period
TimePeriodTrigger=first_detection
SelectionZone=passive_surveillance_post_detection_zone
SelectionProbability=1.0
VisitDelay=Constant 0
VisitFrequency=Constant 1
VisitDuration=Constant 730
DelayToDetection=Constant 0
DetectionRelativeTo=Clinical_signs
DetectionProbability[processor_bison][][]=Constant BetaPert 0.25 0.5 0.75
DetectionProbability[processor_cattle|processor_cattle_l][][]=Constant BetaPert 0.25 0.5 0.75
DetectionProbability[processor_goat|processor_sheep][][]=Constant BetaPert 0.125 0.25 0.375
DetectionProbability[processor_swine|processor_swine_l][][]=Constant BetaPert 0.25 0.5 0.75
DetectionProbability[bison|dealer_bison|livestock_market_bison_cattle|cow_calf_l|cow_calf_s|dairy_l|dairy_s|dairy_heifer_calf_l|dairy
_heifer_calf_m|dairy_heifer_calf_s|feedlot_cattle_l|feedlot_cattle_m|feedlot_cattle_s|stocker_l|stocker_s|dealer_cattle|goat_dairy|go
at_other|livestock_market_goat_sheep|dealer_goat|sheep_l|sheep_s|feedlot_sheep|dealer_sheep|transitional_swine|farrow_to_feeder_l|far
row_to_feeder_s|farrow_to_finish_l|farrow_to_finish_s|farrow_to_wean_l|farrow_to_wean_s|grower_finisher_l|grower_finisher_s|nursery_l
|nursery_s|other_swine_l|other_swine_s|livestock_market_swine|dealer_swine][][]=Constant 0
```

```
[Surveillance6]
ControlName=active_surveillance_livestock_markets
ActivationOption=time_period
TimePeriodTrigger=first_detection
SelectionZone=passive_surveillance_post_detection_zone
SelectionProbability=1.0
VisitDelay=Constant 0
VisitFrequency=Constant 7
VisitDuration=Constant 730
DelayToDetection=Constant 0
DetectionRelativeTo=Clinical_signs
DetectionProbability[livestock_market_bison_cattle][][]=Constant BetaPert 0.12 0.24 0.36
DetectionProbability[livestock_market_goat_sheep][][]=Constant BetaPert 0.04 0.08 0.12
DetectionProbability[livestock_market_swine][][]=Constant BetaPert 0.08 0.16 0.24
DetectionProbability[bison|dealer_bison|cow_calf_l|cow_calf_s|dairy_l|dairy_s|dairy_heifer_calf_l|dairy_heifer_calf_m|dairy_heifer_ca
lf_s|feedlot_cattle_l|feedlot_cattle_m|feedlot_cattle_s|stocker_l|stocker_s|dealer_cattle|goat_dairy|goat_other|dealer_goat|sheep_l|s
heep_s|feedlot_sheep|dealer_sheep|transitional_swine|farrow_to_feeder_l|farrow_to_feeder_s|farrow_to_finish_l|farrow_to_finish_s|farr
ow_to_wean_l|farrow_to_wean_s|grower_finisher_l|grower_finisher_s|nursery_l|nursery_s|other_swine_l|other_swine_s|dealer_swine|proces
sor_bison|processor_cattle|processor_cattle_l|processor_goat|processor_sheep|processor_swine|processor_swine_l][][]=Constant 0

[Surveillance7]
ControlName=control_zone_0km_to_10km_early
ActivationOption=detected_farm
TimePeriodTrigger=first_detection
SurveillanceFarmState=in_0km_to_10km_surveillance_zone
SelectionZone=0km_to_10km_surveillance_zone
SelectionProbability=1.0
VisitDelay=BetaPert 0 8 21
VisitFrequency=Constant 5
VisitDuration=Constant 14
DelayToDetection=BetaPert 0 1 2
DetectionRelativeTo=Clinical_signs
DetectionProbability[][][bison]=Table 1,0,1,43;-1,0.7275,0.97,0.97
DetectionProbability[][][cattle]=Table 1,0,1,43;-1,0.7275,0.97,0.97
DetectionProbability[][][goat]=Table 1,0,1,43;-1,0.7275,0.97,0.97
DetectionProbability[][][sheep]=Table 1,0,1,43;-1,0.7275,0.97,0.97
DetectionProbability[][][sow]=Table 1,0,1,42;1,0.97,0.97,0.97
DetectionProbability[][][piglet]=Table 1,0,1,42;1,0.97,0.97,0.97
DetectionProbability[][][nursery]=Table 1,0,1,42;1,0.97,0.97,0.97
DetectionProbability[][][finisher]=Table 1,0,1,42;1,0.97,0.97,0.97
DetectionProbability[][][other_swine]=Table 1,0,1,42;1,0.97,0.97,0.97

[Surveillance8]
ControlName=control_zone_0km_to_10km_late
ActivationOption=detected_farm
TimePeriodTrigger=first_detection
SurveillanceFarmState=in_0km_to_10km_surveillance_zone
SelectionZone=0km_to_10km_surveillance_zone
SelectionProbability=1.0
VisitDelay=Constant 15
VisitFrequency=Constant 10
VisitDuration=Constant 56
```

```
DelayToDetection=BetaPert 0 0 1
DetectionRelativeTo=Clinical_signs
DetectionProbability[][][bison]=Table 1,0,1,43;-1,0.7275,0.97,0.97
DetectionProbability[][][cattle]=Table 1,0,1,43;-1,0.7275,0.97,0.97
DetectionProbability[][][goat]=Table 1,0,1,43;-1,0.7275,0.97,0.97
DetectionProbability[][][sheep]=Table 1,0,1,43;-1,0.7275,0.97,0.97
DetectionProbability[][][sow]=Table 1,0,1,42;1,0.97,0.97,0.97
DetectionProbability[][][piglet]=Table 1,0,1,42;1,0.97,0.97,0.97
DetectionProbability[][][nursery]=Table 1,0,1,42;1,0.97,0.97,0.97
DetectionProbability[][][finisher]=Table 1,0,1,42;1,0.97,0.97,0.97
DetectionProbability[][][other_swine]=Table 1,0,1,42;1,0.97,0.97,0.97

[Surveillance9]
ControlName=surveillance_zone_10km_to_20km
ActivationOption=detected_farm
TimePeriodTrigger=first_detection
SurveillanceFarmState=in_10km_to_20km_surveillance_zone
SelectionZone=10km_to_20km_surveillance_zone
SelectionProbability=0.65
VisitDelay=BetaPert 1 8 21
VisitFrequency=Constant 21
VisitDuration=Constant 56
DelayToDetection=BetaPert 0 1 2
DetectionRelativeTo=Clinical_signs
DetectionProbability[][][bison]=Table 1,0,1,43;-1,0.7275,0.97,0.97
DetectionProbability[][][cattle]=Table 1,0,1,43;-1,0.7275,0.97,0.97
DetectionProbability[][][goat]=Table 1,0,1,43;-1,0.7275,0.97,0.97
DetectionProbability[][][sheep]=Table 1,0,1,43;-1,0.7275,0.97,0.97
DetectionProbability[][][sow]=Table 1,0,1,42;1,0.97,0.97,0.97
DetectionProbability[][][piglet]=Table 1,0,1,42;1,0.97,0.97,0.97
DetectionProbability[][][nursery]=Table 1,0,1,42;1,0.97,0.97,0.97
DetectionProbability[][][finisher]=Table 1,0,1,42;1,0.97,0.97,0.97
DetectionProbability[][][other_swine]=Table 1,0,1,42;1,0.97,0.97,0.97

[Surveillance10]
ControlName=tracing_livestock_movements1
ActivationOption=tracing
TimePeriodTrigger=first_detection
SurveillanceFarmState=direct_contact_tracing
SelectionProbability=1.0
VisitDelay=BetaPert 0 1 2
VisitFrequency=Constant 5
VisitDuration=Constant 28
DelayToDetection=BetaPert 0 1 2
DetectionRelativeTo=Clinical_signs
DetectionProbability[][][bison]=Table 1,0,1,43;-1,0.7275,0.97,0.97
DetectionProbability[][][cattle]=Table 1,0,1,43;-1,0.7275,0.97,0.97
DetectionProbability[][][goat]=Table 1,0,1,43;-1,0.7275,0.97,0.97
DetectionProbability[][][sheep]=Table 1,0,1,43;-1,0.7275,0.97,0.97
DetectionProbability[][][sow]=Table 1,0,1,42;1,0.97,0.97,0.97
DetectionProbability[][][piglet]=Table 1,0,1,42;1,0.97,0.97,0.97
DetectionProbability[][][nursery]=Table 1,0,1,42;1,0.97,0.97,0.97
```

```
DetectionProbability[][][finisher]=Table 1,0,1,42;1,0.97,0.97,0.97
DetectionProbability[][][other_swine]=Table 1,0,1,42;1,0.97,0.97,0.97



[MovementRestriction1]
ControlName=Standstill_livestock_movements
TimePeriodTrigger=movement_standstill
MovementTypes=bison_&_bison_dealer bison_movement_to_infect_market large_cow_calf_MW_PC large_cow_calf_GL_NE_SE small_cow_calf_MW_PC
small_cow_calf_GL_NE_SE large_dairy small_dairy large_dairy_heifer_calf medium_dairy_heifer_calf small_dairy_heifer_calf
large_medium_cattle_feedlot small_cattle_feedlot large_stocker_GL_MW_NE_PC large_stocker_SE small_stocker_GL_MW_NE_PC
small_stocker_SE cattle_movement_to_infect_market cattle_dealer dairy_goat fiber_meat_goat goat_movement_to_infect_market goat_dealer
large_sheep sheep_to_feedlot small_sheep sheep_feedlot sheep_movement_to_infect_market sheep_dealer sheep_dealer_to_feedlot
transitional_swine farrow nursery grower_finisher other_swine sow_movement_from_large_units sow_movement_from_small_units
transitional_swine_movement_to_infect_market commercial_swine_movement_to_infect_market swine_dealer bison_cattle_through_market
large_dairy_through_market stocker_through_market goat_through_market sheep_through_market transitional_swine_through_market
commercial_swine_through_market bison_market cattle_market goat_market sheep_market swine_market
livestock_movement_to_infect_processor
SourceFarmStates=!detected
ProbMovementRestricted=0.5

[MovementRestriction2]
ControlName=Standstill_indirect_contacts
TimePeriodTrigger=movement_standstill
MovementTypes=indirect_bison_market indirect_cattle_market indirect_goat_market indirect_sheep_market indirect_swine_market
indirect_livestock_processor indirect_medium_risk_GL_MW_NE_SE indirect_medium_risk_PC indirect_low_risk_GL_MW_NE_SE
indirect_low_risk_PC indirect_detected_premises
SourceFarmStates=!detected
ProbMovementRestricted=0.25

[MovementRestriction3]
ControlName=post_standstill_livestock_movements
TimePeriodTrigger=post_movement_standstill
MovementTypes=bison_&_bison_dealer bison_movement_to_infect_market large_cow_calf_MW_PC large_cow_calf_GL_NE_SE small_cow_calf_MW_PC
small_cow_calf_GL_NE_SE large_dairy small_dairy large_dairy_heifer_calf medium_dairy_heifer_calf small_dairy_heifer_calf
large_medium_cattle_feedlot small_cattle_feedlot large_stocker_GL_MW_NE_PC large_stocker_SE small_stocker_GL_MW_NE_PC
small_stocker_SE cattle_movement_to_infect_market cattle_dealer dairy_goat fiber_meat_goat goat_movement_to_infect_market goat_dealer
large_sheep sheep_to_feedlot small_sheep sheep_feedlot sheep_movement_to_infect_market sheep_dealer sheep_dealer_to_feedlot
transitional_swine farrow nursery grower_finisher other_swine sow_movement_from_large_units sow_movement_from_small_units
transitional_swine_movement_to_infect_market commercial_swine_movement_to_infect_market swine_dealer bison_cattle_through_market
large_dairy_through_market stocker_through_market goat_through_market sheep_through_market transitional_swine_through_market
commercial_swine_through_market bison_market cattle_market goat_market sheep_market swine_market
livestock_movement_to_infect_processor
SourceFarmStates=!detected & (in_0km_to_10km_surveillance_zone | direct_contact_tracing | indirect_contact_tracing)
SourceFarmClasses=bison | cow_calf_l | cow_calf_s | dairy_heifer_calf_l | dairy_heifer_calf_m | dairy_heifer_calf_s | dairy_l |
dairy_s | dealer_bison | dealer_cattle | dealer_goat | dealer_sheep | dealer_swine | farrow_to_finish_l | farrow_to_finish_s |
feedlot_cattle_l | feedlot_cattle_m | feedlot_cattle_s | feedlot_sheep | goat_dairy | goat_other | grower_finisher_l |
grower_finisher_s | livestock_market_bison_cattle | livestock_market_goat_sheep | livestock_market_swine | other_swine_l |
other_swine_s | processor_bison | processor_cattle | processor_cattle_l | processor_goat | processor_sheep | processor_swine |
sheep_l | sheep_s | stocker_l | stocker_s | transitional_swine | processor_swine_l
ProbMovementRestricted=0.75
```

```
[MovementRestriction4]
ControlName=post_standstill_indirect_contacts
TimePeriodTrigger=post_movement_standstill
MovementTypes=indirect_bison_market indirect_cattle_market indirect_goat_market indirect_sheep_market indirect_swine_market
indirect_livestock_processor indirect_medium_risk_GL_MW_NE_SE indirect_medium_risk_PC indirect_low_risk_GL_MW_NE_SE
indirect_low_risk_PC indirect_detected_premises
SourceFarmStates=!detected & (in_0km_to_10km_surveillance_zone | direct_contact_tracing | indirect_contact_tracing)
ProbMovementRestricted=0.25

[MovementRestriction5]
ControlName=post_standstill_livestock_movements_swine
TimePeriodTrigger=post_movement_standstill
MovementTypes=farrow nursery
SourceFarmStates=!detected & (in_0km_to_10km_surveillance_zone | direct_contact_tracing | indirect_contact_tracing)
SourceFarmClasses=farrow_to_feeder_l | farrow_to_feeder_s | farrow_to_wean_l | farrow_to_wean_s | nursery_l | nursery_s
ProbMovementRestricted=0.6
```
