# Supplementary material for: Animal disease traceability: evaluation of simulated foot-and-mouth disease outbreak metrics with implementation of improved contact tracing of cattle
Source: Front Vet Sci. 2026 May 5;13:1804982. doi: 10.3389/fvets.2026.1804982 (PMC13196379; doi:10.3389/fvets.2026.1804982)
Supplement: Supplementary file 2 [file Data_Sheet_2.PDF]

Animal Disease Traceability: FMD Improved Tracing

Supplement 2: Farm File Population

bison	2,547	goat_dairy	29,456
cow_calf_l	48,144	goat_other	98,498
cow_calf_s	678,491	grower_finisher_l	6,126
dairy_heifer_calf_l	474	grower_finisher_s	2,134
dairy_heifer_calf_m	1,373	livestock_market_bison_cattle	1,098
dairy_heifer_calf_s	1,498	livestock_market_goat_sheep	673
dairy_l	3,451	livestock_market_swine	876
dairy_s	32,523	nursery_l	1,030
dealer_bison	5	nursery_s	203
dealer_cattle	3,031	other_swine_l	969
dealer_goat	90	other_swine_s	271
dealer_sheep	182	processor_bison	65
dealer_swine	1,702	processor_cattle	625
farrow_to_feeder_l	124	processor_cattle_l	39
farrow_to_feeder_s	353	processor_goat	208
farrow_to_finish_l	1,480	processor_sheep	347
farrow_to_finish_s	2,234	processor_swine	557
farrow_to_wean_l	862	processor_swine_l	40
farrow_to_wean_s	322	sheep_l	2,922
feedlot_cattle_l	43	sheep_s	85,013
feedlot_cattle_m	416	stocker_l	1,871
feedlot_cattle_s	26,126	stocker_s	90,623
feedlot_sheep	23	transitional_swine	47,062
