# Supplementary material for: Animal disease traceability: evaluation of simulated foot-and-mouth disease outbreak metrics with implementation of improved contact tracing of cattle
Source: Front Vet Sci. 2026 May 5;13:1804982. doi: 10.3389/fvets.2026.1804982 (PMC13196379; doi:10.3389/fvets.2026.1804982)
Supplement: Supplementary file 3 [file Data_Sheet_3.pdf]

Animal Disease Traceability: FMD Improved Tracing

Supplement 3: Movement Parameters

Regions as defined in ISP parameter set from USDA-CEAH

Great Lakes	Mountain West	Northeast	Pacific Coast	Southeast
Minnesota	Montana	Maine	California	Arkansas
Iowa	North Dakota	New Hampshire	Oregon	Louisiana
Missouri	South Dakota	Vermont	Washington	Mississippi
Illinois	Idaho	Massachusetts		Tennessee
Indiana	Utah	Rhode Island		Alabama
Ohio	Nevada	Connecticut		Georgia
Wisconsin	Colorado	New Jersey		Florida
Michigan	Arizona	Delaware		North Carolina
Kentucky	New Mexico	Maryland		South Carolina
	Texas	West Virginia		
	Nebraska	Virginia		
	Oklahoma	New York		
	Kansas	Pennsylvania		
	Wyoming			

Direct Farm to Farm Movements:

Origin Farm Type	Number of Farms in Simulation	Poisson Rate/Day	Expected/day	Observed/day
Large Cow-Calf: Mtn West, Pacific Coast	34,247	0.06	2054	2067
Large Cow-Calf all other regions	13,897	0.05	695	692
Small Cow-Calf Mtn West, Pacific Coast	299,979	0.04	1200	1203
Small Cow-Calf All other regions	378,512	0.02	7570	7593
Large Dairy Heifer-Calf	474	0.3885	184	185
Med Dairy Heifer-Calf	1,373	0.0843	116	116
Small Dairy Heifer-Calf	1,498	0.0313	47	47
Large Dairy	3,480	0.1	348	343

Farm to Market Movements:

Origin Farm Type	Number of farms in simulation	Poisson Rate/Day	Expected/day	Observed/Day
Large Cow-Calf	48144	0.012	578	537
Small Cow-Calf	678491	0.0078	5292	5075
Large Dairy Heifer-Calf	474	0.013	6	5
Med Dairy Heifer-Calf	1373	0.0067	9	8
Small Dairy Heifer-Calf	1498	0.0095	14	14
Large Dairy	3480	0.1216	423	302
Small Dairy	60577	0.0374	2266	1994
Large Feedlot	43	0.0206	1	1
Medium Feedlot	416	0.0206	9	8
Small Feedlot	26126	0.0298	779	717
Large Stocker	1871	0.0169	32	28
Small Stocker	90623	0.0096	870	841
