# Supplementary material for: Animal disease traceability: evaluation of simulated foot-and-mouth disease outbreak metrics with implementation of improved contact tracing of cattle
Source: Front Vet Sci. 2026 May 5;13:1804982. doi: 10.3389/fvets.2026.1804982 (PMC13196379; doi:10.3389/fvets.2026.1804982)
Supplement: Supplementary file 4 [file Data_Sheet_4.PDF]

Animal Disease Traceability: FMD with Improved Tracing

Supplement 4: Tracing Parameters

Table 1: Current Tracing Speed Parameters

Beef and Cattle Dealers	**Traces Completed**	0.01	0.03	0.1	0.23	0.41	0.62	0.8	0.93	0.98	0.99	1
	**Day**	0	1	2	3	4	5	6	7	10	14	21
Dairy	**Traces Completed**	0.01	0.11	0.29	0.54	0.77	0.9	0.94	0.96	0.98	0.99	1
	**Day**	0	1	2	3	4	5	6	7	10	14	21

Table 2: Current Tracing Accuracy

Animal Type	Probability movement was forgotten
Beef	0.15
Dairies	0.125
Dairy Heifer/Calf	0.15

Table 3: Partial Tracing Speed Parameters

All Beef and Dairy	**Traces Completed**	0.01	0.23	0.52	0.76	0.91	0.97	0.98	0.99	0.995	0.999	1
	**Day**	0	1	2	3	4	5	6	7	10	14	21

Table 4: Partial Tracing Accuracy Parameter

Animal Type	Probability movement was forgotten
All Beef and Dairy	0.1

Table 5: Ideal Tracing Parameters for all beef and dairy

Speed	Probability movement was forgotten
Beta 0 1 1	0.01
