# Supplementary material for: Animal disease traceability: evaluation of simulated foot-and-mouth disease outbreak metrics with implementation of improved contact tracing of cattle
Source: Front Vet Sci. 2026 May 5;13:1804982. doi: 10.3389/fvets.2026.1804982 (PMC13196379; doi:10.3389/fvets.2026.1804982)
Supplement: Supplementary file 5 [file Data_Sheet_5.PDF]

Animal Disease Traceability: FMD with Improved Tracing

TABLE 1: INFECTED PREMISES CURRENT AND PARTIAL TRACING OUTBREAKS DETECTED ON DAY 21

INTRODUCTION SITE	Percentile	Partial	Current	Current vs Partial Difference	Current vs Partial Confidence Int.
**NEBRASKA FEEDLOT**	10th	46	47	-2	-22, 20
	50th	206	628	-422*	-669, -52
	90th	1,067	2,137	-1,070*	-1,854, -473
**TEXAS FEEDLOT**	10th	61	108	-46*	-93, -4
	50th	279	682	-403*	-715, -157
	90th	1,041	2,248	-1,206*	-1,770, -475
**TENNESSEE DAIRY**	10th	85	203	-118*	-206, -43
	50th	467	1,432	-965*	-1,368, -592
	90th	1,251	2,858	-1,607*	-2,006, -1,106
**CALIFORNIA DAIRY**	10th	164	212	-48	-88, 1
	50th	332	435	-103*	-211, -9
	90th	808	2,096	-1,288*	-1,961, -438
**NEW YORK DAIRY**	10th	131	231	-100*	-212, -14
	50th	454	1,019	-564*	-945, -307
	90th	949	2,683	-1,735*	-2,349, -1,288
**NEBRASKA COW-CALF**	10th	2	2	0	-1, 1
	50th	23	35	-12	-84, 30
	90th	779	1,679	-901*	-1,871, -112
**TENNESSEE COW-CALF**	10th	3	3	0	-2, 2
	50th	19	16	2	-17, 29
	90th	550	1,901	-1,351*	-1,875, -141
**NEW MEXICO STOCKER**	10th	2	3	0	-2, 1
	50th	42	58	-16	-155, 53
	90th	642	1,779	-1,137*	-2,525, -113

TABLE 2: INFECTED PREMISES CURRENT AND IDEAL TRACING OUTBREAKS DETECTED ON DAY 21

INTRODUCTION SITE	Percentile	Ideal	Current	Current vs Ideal Difference	Current vs Ideal Confidence Int.
**NEBRASKA FEEDLOT**	10th	40	47	-7	-29, 7
	50th	128	628	-500*	-751, -156
	90th	414	2,137	-1,723*	-2,461, -1,205
**TEXAS FEEDLOT**	10th	57	108	-50*	-95, -14
	50th	153	682	-529*	-850, -305
	90th	367	2,248	-1,881*	-2,316, -1,423
**TENNESSEE DAIRY**	10th	67	203	-136*	-231, -70
	50th	217	1,432	-1,216*	-1,611, -824
	90th	592	2,858	-2,266*	-2,610, -1,870
**CALIFORNIA DAIRY**	10th	134	212	-79*	-123, -26
	50th	292	435	-144*	-255, -66
	90th	610	2,096	-1,486*	-2,183, -671
**NEW YORK DAIRY**	10th	81	231	-150*	-265, -65
	50th	264	1,019	-755*	-1,112, -485
	90th	533	2,683	-2,151*	-2,803, -1,769
**NEBRASKA COW-CALF**	10th	2	2	0	-1, 1
	50th	16	35	-19	-98, 9
	90th	346	1,679	-1,334*	-2,358, -686
**TENNESSEE COW-CALF**	10th	3	3	0	-2, 2
	50th	14	16	-2	-22, 6
	90th	135	1,901	-1,765*	-2,214, -641
**NEW MEXICO STOCKER**	10th	2	3	0	-2, 1
	50th	29	58	-29	-175, 21
	90th	323	1,779	-1,456*	-2,802, -529

TABLE 3: DURATION OF PARTIAL AND CURRENT TRACING OUTBREAKS DETECTED ON DAY 21

INTRODUCTION SITE	Percentile	Partial	Current	Current vs Partial Difference	Current vs Partial Confidence Int.
**NEBRASKA FEEDLOT**	10th	56	56	0	-12, 11
	50th	108	170	-63*	-109, -13
	90th	233	311	-78*	-184, -24
**TEXAS FEEDLOT**	10th	64	86	-22*	-37, -5
	50th	115	210	-95*	-129, -51
	90th	238	347	-109*	-228, -32
**TENNESSEE DAIRY**	10th	65	95	-30*	-53, -11
	50th	132	234	-102*	-136, -60
	90th	246	380	-134*	-205, -70
**CALIFORNIA DAIRY**	10th	71	86	-15*	-26, -1
	50th	113	139	-26*	-50, -1
	90th	247	376	-130*	-235, -14
**NEW YORK DAIRY**	10th	75	98	-23*	-37, -9
	50th	116	199	-83*	-118, -37
	90th	212	328	-116*	-207, -76
**NEBRASKA COW-CALF**	10th	25	25	0	-2, 2
	50th	57	65	-8	-42, 20
	90th	206	270	-64	-193, 11
**TENNESSEE COW-CALF**	10th	30	30	0	-5, 5
	50th	53	47	6	-9, 22
	90th	183	272	-89	-146, 7
**NEW MEXICO STOCKER**	10th	26	26	0	-5, 5
	50th	68	76	-8	-56, 31
	90th	234	322	-87	-224, 43

TABLE 4: DURATION OF IDEAL AND CURRENT TRACING OUTBREAKS DETECTED ON DAY 21

INTRODUCTION SITE	Percentile	Ideal	Current	Current vs Ideal Difference	Current vs Ideal Confidence Int.
**NEBRASKA FEEDLOT**	10th	52	56	-4	-15, 4
	50th	85	170	-86	-134, -38
	90th	163	311	-148	-261, -98
**TEXAS FEEDLOT**	10th	64	86	-22	-35, -9
	50th	85	210	-125	-158, -86
	90th	146	347	-201	-327, -133
**TENNESSEE DAIRY**	10th	60	95	-35	-56, -19
	50th	95	234	-139	-169, -106
	90th	167	380	-213	-274, -147
**CALIFORNIA DAIRY**	10th	68	86	-18	-28, -8
	50th	97	139	-41	-69, -16
	90th	223	376	-154	-275, -30
**NEW YORK DAIRY**	10th	60	98	-37	-53, -24
	50th	89	199	-109	-145, -65
	90th	147	328	-181	-256, -137
**NEBRASKA COW-CALF**	10th	25	25	-1	-3, 2
	50th	48	65	-17	-50, 5
	90th	135	270	-135	-243, -58
**TENNESSEE COW-CALF**	10th	29	30	0	-5, 5
	50th	44	47	-3	-15, 8
	90th	97	272	-175	-208, -84
**NEW MEXICO STOCKER**	10th	25	26	-1	-6, 4
	50th	56	76	-20	-64, 10
	90th	170	322	-152	-277, -8

TABLE 5: NUMBER OF FARMS IN SURVEILLANCE ZONE CURRENT AND PARTIAL TRACING OUBTREAKS DAY 21 DETECTION

INTRODUCTION SITE	Percentile	Partial	Current	Current vs Partial Difference	Curr vs Part CI
NEBRASKA FEEDLOT	10th	5,158	5,576	-418	-3,332, 2,020
	50th	26,160	72,222	-46,062*	-76,221, -5,679
	90th	127,333	236,822	-109,489*	-195,437, -22,754
TEXAS FEEDLOT	10th	3,942	6,690	-2,748	-7,936, 539
	50th	26,057	74,696	-48,638*	-84,904, -16,078
	90th	127,276	231,935	-104,658*	-184,027, -27,132
TENNESSEE DAIRY	10th	19,381	42,038	-22,657*	-34,957, -8,459
	50th	79,117	183,964	-104,847*	-151,822, -66,925
	90th	179,522	342,900	-163,378*	-209,725, -115,540
CALIFORNIA DAIRY	10th	8,143	10,349	-2,206*	-4,311, -146
	50th	14,594	18,912	-4,318*	-9,845, -947
	90th	39,869	155,004	-115,135*	-206,041, -38,757
NEW YORK DAIRY	10th	16,903	25,678	-8,775*	-19,376, -729
	50th	50,353	115,682	-65,329*	-108,990, -28,529
	90th	113,641	329,053	-215,412*	-268,953, -135,672
NEBRASKA COW-CALF	10th	350	349	1	-92, 94
	50th	2,351	3,347	-996	-10,932, 3,320
	90th	82,615	187,236	-104,621*	-176,604, -7,878
TENNESSEE COW-CALF	10th	738	717	22	-374, 444
	50th	4,508	4,115	393	-4,247, 6,136
	90th	81,339	235,278	-153,939*	-217,411, -32,178
NEW MEXICO STOCKER	10th	95	95	0	-40, 40
	50th	3,556	4,419	-863	-12,554, 4,762
	90th	50,844	179,688	-128,844*	-265,764, -30,870

TABLE 6: NUMBER OF FARMS IN SURVEILLANCE ZONES CURRENT AND IDEAL OUTBREAKS DAY 21 DETECTION

INTRODUCTION SITE	Percentile	Ideal	Current	Current vs Ideal Difference	Curr vs Ideal CI
NEBRASKA FEEDLOT	10th	4,539	5,576	-1,037	-3,882, 783
	50th	14,651	72,222	-57,571*	-85,467, -18,903
	90th	46,116	236,822	-190,706*	-276,242, -127,384
TEXAS FEEDLOT	10th	2,905	6,690	-3,785*	-8,496, -1,127
	50th	12,183	74,696	-62,513*	-94,029, -33,413
	90th	34,998	231,935	-196,937*	-252,546, -145,844
TENNESSEE DAIRY	10th	15,333	42,038	-26,705*	-39,667, -14,559
	50th	42,173	183,964	-141,792*	-185,568, -104,216
	90th	93,715	342,900	-249,185*	-280,374, -206,514
CALIFORNIA DAIRY	10th	7,940	10,349	-2,409*	-4,633, -538
	50th	12,826	18,912	-6,085*	-10,903, -2,988
	90th	25,858	155,004	-129,146*	-215,010, -48,211
NEW YORK DAIRY	10th	10,605	25,678	-15,073*	-26,737, -6,141
	50th	29,775	115,682	-85,907*	-135,247, -50,100
	90th	57,177	329,053	-271,876*	-319,250, -204,562
NEBRASKA COW-CALF	10th	350	349	1	-92, 96
	50th	1,545	3,347	-1,802	-10,985, 1,125
	90th	37,833	187,236	-149,403*	-206,696, -64,473
TENNESSEE COW-CALF	10th	738	717	22	-421, 466
	50th	3,520	4,115	-595	-4,994, 2,089
	90th	27,304	235,278	-207,974*	-271,032, -98,471
NEW MEXICO STOCKER	10th	95	95	0	-40, 35
	50th	1,841	4,419	-2,578	-14,614, 1,687
	90th	29,808	179,688	-149,880*	-289,433, -64,343

TABLE 7: NUMBER OF FARMS IN CONTROL AREAS CURRENT AND PARTIAL TRACING OUTBREAKS DAY 21 DETECTION

INTRODUCTION SITE	Percentile	Partial	Current	Current vs Partial Difference	Current vs Partial Confidence Int.
**NEBRASKA FEEDLOT**	10th	2,573	2,660	-86	-1,307, 995
	50th	13,055	40,562	-27,507*	-44,093, -3,343
	90th	71,241	142,602	-71,362*	-132,102, -22,086
**TEXAS FEEDLOT**	10th	2,045	3,530	-1,486	-4,236, 242
	50th	13,540	41,943	-28,403*	-47,059, -10,904
	90th	72,831	138,231	-65,400*	-112,302, -27,254
**TENNESSEE DAIRY**	10th	10,035	22,088	-12,053*	-19,742, -4,664
	50th	44,854	113,182	-68,327*	-96,796, -42,100
	90th	105,640	209,969	-104,329*	-132,264, -71,182
**CALIFORNIA DAIRY**	10th	6,646	9,213	-2,566*	-4,532, -229
	50th	13,204	16,639	-3,435*	-7,434, -542
	90th	32,987	101,302	-68,314*	-128,649, -23,487
**NEW YORK DAIRY**	10th	9,678	15,884	-6,206*	-13,555, -938
	50th	31,079	72,173	-41,094*	-68,977, -20,063
	90th	70,152	200,493	-130,340*	-167,435, -90,498
**NEBRASKA COW-CALF**	10th	134	134	0	-45, 43
	50th	1,101	1,597	-496	-4,751, 1,455
	90th	45,567	110,879	-65,312*	-112,382, -11,588
**TENNESSEE COW-CALF**	10th	311	307	4	-151, 153
	50th	2,069	1,867	202	-2,056, 3,432
	90th	46,805	144,764	-97,959*	-138,537, -26,154
**NEW MEXICO STOCKER**	10th	77	77	-1	-41, 31
	50th	2,219	2,622	-403	-9,457, 2,880
	90th	30,684	106,828	-76,144*	-155,902, -21,041

TABLE 8: NUMBER OF FARMS IN CONTROL AREAS CURRENT AND IDEAL TRACING OUTBREAKS DAY 21 DETECTION

INTRODUCTION SITE	Percentile	Ideal	Current	Current vs Ideal Difference	Current vs Ideal Confidence Int.
**NEBRASKA FEEDLOT**	10th	2,269	2,660	-391	-1,546, 500
	50th	7,434	40,562	-33,128*	-49,632, -9,225
	90th	24,215	142,602	-118,387*	-164,827, -82,462
**TEXAS FEEDLOT**	10th	1,559	3,530	-1,972*	-4,398, -642
	50th	6,417	41,943	-35,526*	-54,561, -17,537
	90th	18,227	138,231	-120,003*	-151,853, -90,488
**TENNESSEE DAIRY**	10th	7,728	22,088	-14,360*	-22,041, -7,957
	50th	22,657	113,182	-90,524*	-116,063, -65,376
	90th	55,272	209,969	-154,697*	-179,603, -124,748
**CALIFORNIA DAIRY**	10th	6,044	9,213	-3,168*	-5,262, -1,137
	50th	11,388	16,639	-5,251*	-9,208, -2,542
	90th	21,589	101,302	-79,712*	-137,449, -36,501
**NEW YORK DAIRY**	10th	5,796	15,884	-10,088*	-16,851, -4,502
	50th	17,835	72,173	-54,338*	-80,922, -34,624
	90th	36,038	200,493	-164,455*	-199,737, -127,479
**NEBRASKA COW-CALF**	10th	134	134	0	-45, 45
	50th	732	1,597	-865	-5,082, 428
	90th	19,927	110,879	-90,952*	-128,694, -42,066
**TENNESSEE COW-CALF**	10th	311	307	4	-145, 156
	50th	1,611	1,867	-257	-2,616, 964
	90th	14,460	144,764	-130,304*	-164,753, -53,447
**NEW MEXICO STOCKER**	10th	77	77	-1	-41, 31
	50th	1,166	2,622	-1,456	-8,643, 1,124
	90th	17,752	106,828	-89,077*	-166,170, -40,117

Tables for Objective 2: Reduction of Control Areas and Surveillance Zones

TABLE 9: NUMBER OF IPS IDEAL TRACING WITH STANDARD AND REDUCED SURVEILLANCE AREAS

INTRODUCTION SITE	Percentile	Reduced	Standard	Reduced vs Standard Difference	Reduced vs Standard Confidence Int.
**NEBRASKA FEEDLOT**	10th	43	40	3	-9, 14
	50th	161	128	33	-32, 92
	90th	513	414	100	-80, 274
**TEXAS FEEDLOT**	10th	62	57	5	-15, 30
	50th	180	153	27	-10, 82
	90th	656	367	289*	2, 536
**TENNESSEE DAIRY**	10th	71	67	4	-16, 35
	50th	238	217	22	-57, 97
	90th	774	592	182	-40, 363
**CALIFORNIA DAIRY**	10th	143	134	10	-34, 70
	50th	347	292	56	-12, 129
	90th	887	610	277	-114, 769
**NEW YORK DAIRY**	10th	107	81	25	-42, 93
	50th	314	264	51	-23, 122
	90th	683	533	150	-2, 362
**NEBRASKA COW-CALF**	10th	2	2	0	-1, 1
	50th	16	16	0	-15, 19
	90th	350	346	5	-213, 234
**TENNESSEE COW-CALF**	10th	4	3	0	-2, 3
	50th	15	14	1	-8, 9
	90th	297	135	161	-33, 347
**NEW MEXICO STOCKER**	10th	3	2	0	-1, 1
	50th	32	29	3	-38, 46
	90th	300	323	-23	-375, 408

TABLE 10: DURATION OF OUTBREAKS IDEAL TRACING WITH STANDARD AND REDUCED SURVEILLANCE AREAS

INTRODUCTION SITE	Percentile	Reduced	Standard	Reduced vs Standard Difference	Reduced vs Standard CI
**NEBRASKA FEEDLOT**	10th	54	52	3	-5, 11
	50th	90	85	5	-11, 20
	90th	181	163	18	-33, 72
**TEXAS FEEDLOT**	10th	64	64	0	-9, 11
	50th	100	85	15*	1, 28
	90th	217	146	71*	19, 122
**TENNESSEE DAIRY**	10th	61	60	1	-8, 14
	50th	101	95	5	-7, 21
	90th	183	167	16	-25, 47
**CALIFORNIA DAIRY**	10th	77	68	9*	1, 18
	50th	123	97	26*	6, 49
	90th	271	223	48	-28, 202
**NEW YORK DAIRY**	10th	69	60	9	-2, 23
	50th	107	89	17*	5, 31
	90th	178	147	31	-5, 59
**NEBRASKA COW-CALF**	10th	25	25	0	-2, 2
	50th	46	48	-1	-13, 12
	90th	170	135	36	-55, 104
**TENNESSEE COW-CALF**	10th	29	29	-1	-6, 5
	50th	46	44	2	-9, 11
	90th	128	97	31	-11, 66
**NEW MEXICO STOCKER**	10th	25	25	0	-4, 5
	50th	59	56	2	-20, 24
	90th	184	170	14	-117, 91

TABLE 11: FARMS IN SURVEILLANCE ZONES IDEAL TRACING WITH STANDARD AND REDUCED SURVEILLANCE AREAS

INTRODUCTION SITE	Percentile	Reduced	Standard	Reduced vs Standard Difference	Reduced vs Standard CI
**NEBRASKA FEEDLOT**	10th	2,955	4,539	-1,584*	-3741, -166
	50th	11,226	14,651	-3,425	-8,061, 982
	90th	36,962	46,116	-9,154	-22,756, 3,958
**TEXAS FEEDLOT**	10th	2,375	2,905	-530	-2,229, 800
	50th	9,938	12,183	-2,245	-7,505, 2,790
	90th	43,628	34,998	8,630	-17,084, 24,821
**TENNESSEE DAIRY**	10th	10,353	15,333	-4,980*	-9,010, -139
	50th	28,761	42,173	-13,412*	-25,481, -3,907
	90th	75,571	93,715	-18,144*	-40,417, -2,324
**CALIFORNIA DAIRY**	10th	6,031	7,940	-1,909	-3,710, 893
	50th	12,148	12,826	-678	-3,018, 1,782
	90th	27,467	25,858	1,609	-8,658, 11,552
**NEW YORK DAIRY**	10th	8,125	10,605	-2,480	-8,603, 3,310
	50th	23,649	29,775	-6,126	-14,040, 257
	90th	51,950	57,177	-5,227	-20,848, 7,001
**NEBRASKA COW-CALF**	10th	191	350	-158*	-256, -60
	50th	927	1,545	-619	-1,990, 607
	90th	21,794	37,833	-16,039	-41,555, 5,151
**TENNESSEE COW-CALF**	10th	339	738	-399*	-895, -65
	50th	2,077	3,520	-1,443*	-3,701, -28
	90th	33,125	27,304	5,821	-12,598, 19,678
**NEW MEXICO STOCKER**	10th	72	95	-23	-71, 19
	50th	1,460	1,841	-381	-3,123, 1,354
	90th	19,836	29,808	-9,972	-28,286, 15,017

TABLE 12: FARMS IN CONTROL AREAS IDEAL TRACING WITH STANDARD AND REDUCED SURVEILLANCE AREAS

INTRODUCTION SITE	Percentile	Reduced	Standard	Reduced vs Standard Difference	Reduced vs Standard CI
**NEBRASKA FEEDLOT**	10th	1,363	2,269	-906*	-1,978, -335
	50th	5,238	7,434	-2,197	-4,736, 175
	90th	18,040	24,215	-6,174	-12,400, 8
**TEXAS FEEDLOT**	10th	1,094	1,559	-465	-1,255, 272
	50th	4,761	6,417	-1,657	-4,048, 928
	90th	21,049	18,227	2,821	-7,992, 12,005
**TENNESSEE DAIRY**	10th	4,723	7,728	-3,005*	-5,074, -399
	50th	14,170	22,657	-8,487*	-14,634, -2,162
	90th	40,104	55,272	-15,167*	-27,919, -4,877
**CALIFORNIA DAIRY**	10th	4,314	6,044	-1,730	-3,451, 570
	50th	9,755	11,388	-1,633	-3,792, 180
	90th	21,001	21,589	-588	-8,180, 5,055
**NEW YORK DAIRY**	10th	4,001	5,796	-1,796	-5,297, 1,389
	50th	12,881	17,835	-4,954*	-9,950, -697
	90th	29,747	36,038	-6,291	-15,502, 1,242
**NEBRASKA COW-CALF**	10th	63	134	-71*	-118, -32
	50th	395	732	-337	-1,000, 220
	90th	11,361	19,927	-8,566	-22,510, 3,288
**TENNESSEE COW-CALF**	10th	176	311	-135*	-329, -10
	50th	954	1,611	-657	-1,822, 37
	90th	16,069	14,460	1,609	-82,88, 9,071
**NEW MEXICO STOCKER**	10th	53	77	-24	-62, 11
	50th	899	1,166	-268	-1,832, 713
	90th	10,902	17,752	-6,850	-18,737, 5,323

TABLE 11: FARMS IN SURVEILLANCE ZONES IDEAL TRACING WITH STANDARD AND REDUCED SURVEILLANCE AREAS

INTRODUCTION SITE	Percentile	Reduced	Standard	Reduced vs Standard Difference	Reduced vs Standard CI
NEBRASKA FEEDLOT	10th	2,955	4,539	-1,584*	-3741, -166
	50th	11,226	14,651	-3,425	-8,061, 982
	90th	36,962	46,116	-9,154	-22,756, 3,958
TEXAS FEEDLOT	10th	2,375	2,905	-530	-2,229, 800
	50th	9,938	12,183	-2,245	-7,505, 2,790
	90th	43,628	34,998	8,630	-17,084, 24,821
TENNESSEE DAIRY	10th	10,353	15,333	-4,980*	-9,010, -139
	50th	28,761	42,173	-13,412*	-25,481, -3,907
	90th	75,571	93,715	-18,144*	-40,417, -2,324
CALIFORNIA DAIRY	10th	6,031	7,940	-1,909	-3,710, 893
	50th	12,148	12,826	-678	-3,018, 1,782
	90th	27,467	25,858	1,609	-8,658, 11,552
NEW YORK DAIRY	10th	8,125	10,605	-2,480	-8,603, 3,310
	50th	23,649	29,775	-6,126	-14,040, 257
	90th	51,950	57,177	-5,227	-20,848, 7,001
NEBRASKA COW-CALF	10th	191	350	-158*	-256, -60
	50th	927	1,545	-619	-1,990, 607
	90th	21,794	37,833	-16,039	-41,555, 5,151
TENNESSEE COW-CALF	10th	339	738	-399*	-895, -65
	50th	2,077	3,520	-1,443*	-3,701, -28
	90th	33,125	27,304	5,821	-12,598, 19,678
NEW MEXICO STOCKER	10th	72	95	-23	-71, 19
	50th	1,460	1,841	-381	-3,123, 1,354
	90th	19,836	29,808	-9,972	-28,286, 15,017
