# Supplementary material for: Animal disease traceability: evaluation of simulated foot-and-mouth disease outbreak metrics with implementation of improved contact tracing of cattle
Source: Front Vet Sci. 2026 May 5;13:1804982. doi: 10.3389/fvets.2026.1804982 (PMC13196379; doi:10.3389/fvets.2026.1804982)
Supplement: Supplementary file 6 [file Data_Sheet_6.PDF]

Animal Disease Traceability: FMD with Improved Tracing

Supplement 6: Outbreak Metrics for Day 8 and 14

Number of Infected Premises from outbreaks detected on Day 8

Nebraska Feedlot Detected Day 8: Partial					
Percentile	Partial Tracing	Current Tracing	Estimated Difference	ci_lower	ci_upper
10	1	1	0	0	0
25	2	2	0	-1	1
50	3	3	0	-2	2
75	13	12	1	-29	18
90	86	147	-61	-648	396

Nebraska Feedlot Detected Day 8: Ideal					
Percentile	Ideal Tracing	Current Tracing	Estimated Difference	ci_lower	ci_upper
10	1	1	0	0	0
25	2	2	0	-1	1
50	3	3	0	-2	2
75	9	12	-2	-31	8
90	28	147	-120	-718	10

Texas Feedlot Detected Day 8: Partial					
Percentile	Partial Tracing	Current Tracing	Estimated Difference	ci_lower	ci_upper
10	1	1	0	-1	1
25	2	2	0	0	0
50	3	3	0	-1	1
75	10	11	-1	-32	17
90	68	234	-165	-887	140

Texas Feedlot Detected Day 8: Ideal					
Percentile	Ideal Tracing	Current Tracing	Estimated Difference	ci_lower	ci_upper
10	1	1	0	-1	0
25	2	2	0	0	1
50	3	3	0	-1	1
75	9	11	-2	-29	8
90	49	234	-185	-1054	57

Tennessee Dairy Detected Day 8: Partial					
Percentile	Partial Tracing	Current Tracing	Estimated Difference	ci_lower	ci_upper

10	1	1	0	-1	1
25	3	2	0	-1	1
50	7	8	0	-8	8
75	44	97	-52	-371	52
90	288	1306	-1019	-2250	130

Tennessee Dairy Detected Day 8: Ideal					
Percentile	Ideal Tracing	Current Tracing	Estimated Difference	ci_lower	ci_upper
10	1	1	0	-1	1
25	2	2	0	-1	1
50	6	8	-2	-9	2
75	18	97	-79	-406	3
90	72	1306	-1234	-2383	-168

California Dairy Detected Day 8: Partial					
Percentile	Partial Tracing	Current Tracing	Estimated Difference	ci_lower	ci_upper
10	2	2	0	-1	1
25	4	4	-1	-4	2
50	15	23	-9	-38	12
75	84	115	-31	-125	39
90	211	475	-264	-1656	88

California Dairy Detected Day 8: Ideal					
Percentile	Ideal Tracing	Current Tracing	Estimated Difference	ci_lower	ci_upper
10	2	2	0	-1	1
25	4	4	0	-4	2
50	12	23	-12	-41	4
75	38	115	-76	-177	-15
90	101	475	-374	-1668	-35

Number of Infected Premises from outbreaks detected on Day 8

New York Dairy Detected Day 8: Partial

Percentile	Partial Tracing	Current Tracing	Estimated Difference	ci_lower	ci_upper
10	2	2	0	-1	1
25	3	4	0	-3	2
50	9	12	-3	-20	6
75	41	145	-104	-242	17
90	255	616	-360	-999	98

New York Dairy Detected Day 8: Ideal					
Percentile	Ideal Tracing	Current Tracing	Estimated Difference	ci_lower	ci_upper
10	2	2	0	-1	1
25	4	4	0	-3	2
50	8	12	-4	-23	3
75	24	145	-121	-280	-21
90	78	616	-537	-1186	-146

Nebraska Cow-Calf Detected Day 8: Partial					
Percentile	Partial Tracing	Current Tracing	Estimated Difference	ci_lower	ci_upper
10	1	1	0	0	0
25	1	1	0	0	0
50	1	1	0	-1	1
75	3	3	0	-5	3
90	18	26	-8	-145	51

Nebraska Cow-Calf Detected Day 8: Ideal					
Percentile	Ideal Tracing	Current Tracing	Estimated Difference	ci_lower	ci_upper
10	1	1	0	0	0
25	1	1	0	0	0
50	1	1	0	-1	1
75	3	3	0	-6	2
90	10	26	-16	-164	7

Number of Infected Premises from outbreaks detected on Day 8

Tennessee Cow-Calf Detected Day 8: Partial

Percentile	Partial Tracing	Current Tracing	Estimated Difference	ci_lower	ci_upper
10	1	1	0	0	0
25	1	1	0	0	0
50	2	2	0	-1	1
75	4	4	0	-2	2
90	9	28	-19	-624	73

Tennessee Cow-Calf Detected Day 8: Ideal					
Percentile	Ideal Tracing	Current Tracing	Estimated Difference	ci_lower	ci_upper
10	1	1	0	0	0
25	1	1	0	0	0
50	2	2	0	-1	1
75	4	4	0	-2	2
90	10	28	-18	-528	31

New Mexico Stocker Detected Day 8: Partial					
Percentile	Partial Tracing	Current Tracing	Estimated Difference	ci_lower	ci_upper
10	1	1	0	0	0
25	1	1	0	0	0
50	1	1	0	0	1
75	3	3	0	-4	3
90	11	21	-10	-602	15

New Mexico Stocker Detected Day 8: Ideal					
Percentile	Ideal Tracing	Current Tracing	Estimated Difference	ci_lower	ci_upper
10	1	1	0	0	0
25	1	1	0	0	0
50	1	1	0	-1	1
75	3	3	0	-4	2
90	10	21	-11	-641	11

Duration (days) of outbreaks detected on Day 8

Nebraska Feedlot Duration Day 8: Partial

Percentile	Partial Tracing	Current Tracing	Estimated Difference	ci_lower	ci_upper
10	8	8	0	0	0
25	13	12	1	-7	8
50	24	23	0	-7	7
75	39	39	0	-28	16
90	93	111	-18	-109	90

Nebraska Feedlot Duration Day 8: Ideal					
Percentile	Ideal Tracing	Current Tracing	Estimated Difference	ci_lower	ci_upper
10	8	8	0	0	0
25	12	12	0	-7	7
50	24	23	0	-6	7
75	35	39	-4	-37	8
90	52	111	-58	-126	6

Texas Feedlot Duration Day 8: Partial					
Percentile	Partial Tracing	Current Tracing	Estimated Difference	ci_lower	ci_upper
10	8	8	0	-3	2
25	14	15	0	-4	4
50	18	18	0	-6	4
75	30	36	-5	-32	10
90	69	114	-45	-166	45

Texas Feedlot Duration Day 8: Ideal					
Percentile	Ideal Tracing	Current Tracing	Estimated Difference	ci_lower	ci_upper
10	8	8	0	-3	2
25	14	15	0	-4	4
50	18	18	0	-6	4
75	30	36	-5	-32	10
90	69	114	-45	-166	45

Duration (days) of outbreaks detected on Day 8

Tennessee Dairy Duration Day 8: Partial

Percentile	Partial Tracing	Current Tracing	Estimated Difference	ci_lower	ci_upper
10	8	8	0	-3	3
25	13	14	0	-7	5
50	31	30	0	-11	12
75	63	85	-22	-92	27
90	122	231	-109	-239	16

Tennessee Dairy Duration Day 8: Ideal					
Percentile	Ideal Tracing	Current Tracing	Estimated Difference	ci_lower	ci_upper
10	8	8	0	-3	2
25	13	14	-1	-6	3
50	23	30	-8	-19	4
75	45	85	-40	-114	3
90	76	231	-155	-281	-46

California Dairy Duration Day 8: Partial					
Percentile	Partial Tracing	Current Tracing	Estimated Difference	ci_lower	ci_upper
10	11	11	0	-4	4
25	18	19	-1	-10	6
50	40	48	-9	-29	16
75	87	104	-18	-61	22
90	133	187	-54	-290	29

California Dairy Duration Day 8: Ideal					
Percentile	Ideal Tracing	Current Tracing	Estimated Difference	ci_lower	ci_upper
10	11	11	-1	-4	3
25	18	19	-1	-9	4
50	33	48	-16	-35	4
75	59	104	-45	-88	-2
90	99	187	-88	-321	14

Duration (days) of outbreaks detected on Day 8

New York Dairy Duration Day 8: Partial

Percentile	Partial Tracing	Current Tracing	Estimated Difference	ci_lower	ci_upper
10	10	10	0	-4	4
25	16	19	-2	-8	4
50	30	36	-7	-33	8
75	60	105	-45	-81	-3
90	129	194	-65	-160	24

New York Dairy Duration Day 8: Ideal					
Percentile	Ideal Tracing	Current Tracing	Estimated Difference	ci_lower	ci_upper
10	10	10	0	-4	3
25	16	19	-2	-9	5
50	28	36	-8	-35	4
75	50	105	-56	-92	-19
90	81	194	-113	-186	-44

Nebraska Cow Calf Duration Day 8: Partial					
Percentile	Partial Tracing	Current Tracing	Estimated Difference	ci_lower	ci_upper
10	8	8	0	0	0
25	8	8	0	0	0
50	9	10	0	-6	5
75	19	20	-1	-14	8
90	44	47	-3	-52	32

Nebraska Cow Calf Duration Day 8: Ideal					
Percentile	Ideal Tracing	Current Tracing	Estimated Difference	ci_lower	ci_upper
10	8	8	0	0	0
25	8	8	0	0	0
50	9	10	-1	-5	4
75	17	20	-3	-14	6
90	35	47	-11	-60	15

Duration (days) of outbreaks detected on Day 8

Tennessee Cow Calf Duration Day 8: Partial

Percentile	Partial Tracing	Current Tracing	Estimated Difference	ci_lower	ci_upper
10	8	8	0	0	0
25	8	8	0	-1	0
50	14	14	0	-5	5
75	22	23	-1	-8	6
90	37	47	-10	-124	42

Tennessee Cow Calf Duration Day 8: Ideal					
Percentile	Ideal Tracing	Current Tracing	Estimated Difference	ci_lower	ci_upper
10	8	8	0	0	0
25	8	8	0	-1	0
50	14	14	0	-6	5
75	23	23	0	-6	7
90	37	47	-11	-128	26

New Mexico Stocker Duration Day 8: Partial					
Percentile	Partial Tracing	Current Tracing	Estimated Difference	ci_lower	ci_upper
10	8	8	0	0	0
25	8	8	0	0	0
50	8	8	0	-2	2
75	17	19	-2	-15	10
90	37	48	-11	-132	21

New Mexico Stocker Duration Day 8: Ideal					
Percentile	Ideal Tracing	Current Tracing	Estimated Difference	ci_lower	ci_upper
10	8	8	0	0	0
25	8	8	0	0	0
50	8	8	0	-3	3
75	17	19	-2	-15	7
90	33	48	-15	-118	9

Number of Farms in Control Areas from Outbreaks Detected on Day 8

Nebraska Feedlot Control Area Day 8: Partial

Percentile	Partial Tracing	Current Tracing	Estimated Difference	ci_lower	ci_upper
10	134	134	0	0	0
25	142	142	0	-49	51
50	290	276	14	-146	159
75	822	767	55	-1,774	1,241
90	5,585	10,842	-5,257	-46,624	27,888

Nebraska Feedlot Control Area Day 8: Ideal					
Percentile	Ideal Tracing	Current Tracing	Estimated Difference	ci_lower	ci_upper
10	134	134	0	0	0
25	142	142	0	-40	43
50	277	276	0	-126	127
75	656	767	-110	-2,586	487
90	1,918	10,842	-8,924	-48,188	746

Texas Feedlot Control Area Day 8: Partial					
Percentile	Partial Tracing	Current Tracing	Estimated Difference	ci_lower	ci_upper
10	7	7	0	-5	5
25	18	18	0	-8	8
50	46	47	-2	-49	44
75	275	250	25	-935	585
90	3,082	13,223	-10,141	-65,992	7,101

Texas Feedlot Control Area Day 8: Ideal					
Percentile	Ideal Tracing	Current Tracing	Estimated Difference	ci_lower	ci_upper
10	7	7	0	-5	5
25	18	18	0	-8	8
50	45	47	-2	-43	36
75	200	250	-50	-1,599	324
90	1,960	13,223	-11,263	-67,700	3,908

Number of Farms in Control Areas from Outbreaks Detected on Day 8

Tennessee Dairy Control Area Day 8: Partial

Percentile	Partial Tracing	Current Tracing	Estimated Difference	ci_lower	ci_upper
10	87	87	0	-77	79
25	282	279	3	-198	206
50	974	988	-14	-1,051	937
75	5,400	11,440	-6,040	-31,346	5,473
90	27,174	99,066	-71,892	-150,895	10,519

Tennessee Dairy Control Area Day 8: Ideal					
Percentile	Ideal Tracing	Current Tracing	Estimated Difference	ci_lower	ci_upper
10	87	87	0	-85	81
25	272	279	-7	-183	155
50	720	988	-268	-1,378	271
75	2,291	11,440	-9,149	-35,843	-103
90	8,323	99,066	-90,743	-169,969	-16,497

California Dairy Control Area Day 8: Partial					
Percentile	Partial Tracing	Current Tracing	Estimated Difference	ci_lower	ci_upper
10	92	93	0	-66	63
25	229	243	-15	-246	186
50	851	1,408	-557	-2,155	640
75	4,547	6,019	-1,472	-6,118	2,276
90	10,640	20,343	-9,703	-71,507	4,403

California Dairy Control Area Day 8: Ideal					
Percentile	Ideal Tracing	Current Tracing	Estimated Difference	ci_lower	ci_upper
10	92	93	0	-68	64
25	221	243	-22	-271	149
50	635	1,408	-773	-2,159	115
75	2,267	6,019	-3,752	-7,976	-393
90	5,224	20,343	-15,118	-75,020	-2,979

Number of Farms in Control Areas from Outbreaks Detected on Day 8

New York Dairy Control Area Day 8: Partial

Percentile	Partial Tracing	Current Tracing	Estimated Difference	ci_lower	ci_upper
10	113	113	0	-70	70
25	252	272	-20	-227	169
50	729	1,027	-298	-1,855	370
75	3,550	11,397	-7,847	-18,833	1,350
90	19,955	46,967	-27,013	-76,720	6,031

New York Dairy Control Area Day 8: Ideal					
Percentile	Ideal Tracing	Current Tracing	Estimated Difference	ci_lower	ci_upper
10	113	113	-1	-70	67
25	275	272	3	-197	164
50	659	1,027	-368	-1,701	201
75	1,841	11,397	-9,556	-21,045	-1,447
90	6,969	46,967	-39,998	-89,733	-9,257

Nebraska Cow Calf Control Area Day 8: Partial					
Percentile	Partial Tracing	Current Tracing	Estimated Difference	ci_lower	ci_upper
10	91	91	0	0	0
25	91	91	0	0	0
50	94	95	-1	-32	28
75	203	212	-9	-316	162
90	933	1,452	-519	-8,596	3,303

Nebraska Cow Calf Control Area Day 8: Ideal					
Percentile	Ideal Tracing	Current Tracing	Estimated Difference	ci_lower	ci_upper
10	91	91	0	0	0
25	91	91	0	0	0
50	94	95	-1	-33	30
75	186	212	-25	-325	115
90	554	1,452	-897	-9,071	446

Number of Farms in Control Areas from Outbreaks Detected on Day 8

Tennessee Cow Calf Control Area Day 8: Partial

Percentile	Partial Tracing	Current Tracing	Estimated Difference	ci_lower	ci_upper
10	248	248	0	0	0
25	248	248	0	-1	1
50	266	266	0	-54	46
75	525	532	-7	-382	318
90	1,375	3,297	-1,922	-42,992	5,264

Tennessee Cow Calf Control Area Day 8: Ideal					
Percentile	Ideal Tracing	Current Tracing	Estimated Difference	ci_lower	ci_upper
10	248	248	0	0	0
25	248	248	0	-1	1
50	266	266	0	-29	27
75	520	532	-13	-411	318
90	1,436	3,297	-1,862	-47,569	3,678

New Mexico Stocker Control Area Day 8: Partial					
Percentile	Partial Tracing	Current Tracing	Estimated Difference	ci_lower	ci_upper
10	68	68	0	0	0
25	68	68	0	0	0
50	68	68	0	-1	1
75	94	97	-2	-209	84
90	392	1,453	-1,062	-31,234	1,299

New Mexico Stocker Control Area Day 8: Ideal					
Percentile	Ideal Tracing	Current Tracing	Estimated Difference	ci_lower	ci_upper
10	68	68	0	0	0
25	68	68	0	0	0
50	68	68	0	-2	1
75	89	97	-7	-183	66
90	392	1,453	-1,062	-28,028	557

Number of Farms in Surveillance Zones from Outbreaks Detected on Day 8

Nebraska Feedlot Surveillance Zone Day 8: Partial

Percentile	Partial Tracing	Current Tracing	Estimated Difference	ci_lower	ci_upper
10	380	380	0	0	0
25	392	392	0	-95	96
50	703	680	23	-195	242
75	1,791	1,708	83	-3,863	2,759
90	11,815	22,397	-10,582	-84,380	42,252

Nebraska Feedlot Surveillance Zone Day 8: Ideal					
Percentile	Ideal Tracing	Current Tracing	Estimated Difference	ci_lower	ci_upper
10	380	380	0	0	0
25	392	392	0	-94	87
50	680	680	0	-205	195
75	1,471	1,708	-237	-5,755	1,109
90	4,244	22,397	-18,153	-83,935	1,628

Texas Feedlot Surveillance Zone Day 8: Partial					
Percentile	Partial Tracing	Current Tracing	Estimated Difference	ci_lower	ci_upper
10	33	33	0	-9	9
25	63	63	0	-28	26
50	117	117	0	-94	94
75	552	569	-17	-1,607	942
90	5,952	25,021	-19,069	-105,546	13,808

Texas Feedlot Surveillance Zone Day 8: Ideal					
Percentile	Ideal Tracing	Current Tracing	Estimated Difference	ci_lower	ci_upper
10	33	33	0	-12	14
25	63	63	0	-22	20
50	116	117	0	-98	83
75	440	569	-129	-2,303	615
90	3,247	25,021	-21,775	-118,206	5,039

Number of Farms in Surveillance Zones from Outbreaks Detected on Day 8

Tennessee Dairy Surveillance Zone Day 8: Partial

Percentile	Partial Tracing	Current Tracing	Estimated Difference	ci_lower	ci_upper
10	564	564	0	-260	255
25	1,042	1,050	-8	-340	350
50	2,510	2,624	-113	-2,284	1,959
75	11,620	22,057	-10,437	-54,196	11,023
90	50,190	165,677	-115,487	-251,877	11,617

Tennessee Dairy Surveillance Zone Day 8: Ideal					
Percentile	Ideal Tracing	Current Tracing	Estimated Difference	ci_lower	ci_upper
10	564	564	0	-219	226
25	1,025	1,050	-25	-432	326
50	1,951	2,624	-673	-2,928	664
75	5,159	22,057	-16,898	-70,746	-230
90	16,542	165,677	-149,135	-274,297	-31,523

California Dairy Surveillance Zone Day 8: Partial					
Percentile	Partial Tracing	Current Tracing	Estimated Difference	ci_lower	ci_upper
10	283	288	-5	-199	150
25	643	701	-58	-398	283
50	1,661	2,333	-672	-2,871	1,040
75	6,310	7,912	-1,602	-7,059	2,018
90	13,094	24,132	-11,038	-102,246	7,040

California Dairy Surveillance Zone Day 8: Ideal					
Percentile	Ideal Tracing	Current Tracing	Estimated Difference	ci_lower	ci_upper
10	283	288	-5	-211	174
25	633	701	-68	-443	203
50	1,349	2,333	-983	-2,866	336
75	3,482	7,912	-4,430	-9,632	-872
90	7,212	24,132	-16,920	-103,779	-2,732

Number of Farms in Surveillance Zones from Outbreaks Detected on Day 8

New York Dairy Surveillance Zone Day 8: Partial

Percentile	Partial Tracing	Current Tracing	Estimated Difference	ci_lower	ci_upper
10	325	325	0	-174	154
25	631	676	-44	-491	298
50	1,731	2,378	-647	-3,162	997
75	7,260	19,892	-12,631	-31,924	3,663
90	32,563	78,601	-46,038	-132,618	9,092

New York Dairy Surveillance Zone Day 8: Ideal					
Percentile	Ideal Tracing	Current Tracing	Estimated Difference	ci_lower	ci_upper
10	325	325	0	-165	179
25	652	676	-24	-488	275
50	1,599	2,378	-779	-3,050	355
75	4,088	19,892	-15,803	-33,607	-1,773
90	13,444	78,601	-65,157	-143,386	-18,659

Nebraska Cow Calf Surveillance Zone Day 8: Partial					
Percentile	Partial Tracing	Current Tracing	Estimated Difference	ci_lower	ci_upper
10	241	241	0	0	0
25	241	241	0	0	0
50	248	249	-1	-84	76
75	504	527	-23	-567	349
90	1,890	3,041	-1,151	-19,668	7,572

Nebraska Cow Calf Surveillance Zone Day 8: Ideal					
Percentile	Ideal Tracing	Current Tracing	Estimated Difference	ci_lower	ci_upper
10	241	241	0	0	0
25	241	241	0	0	0
50	248	249	-1	-90	83
75	460	527	-67	-587	284
90	1,219	3,041	-1,822	-18,749	1,402

Number of Farms in Surveillance Zones from Outbreaks Detected on Day 8

Tennessee Cow Calf Surveillance Zone Day 8: Partial

Percentile	Partial Tracing	Current Tracing	Estimated Difference	ci_lower	ci_upper
10	562	562	0	0	0
25	562	562	0	-3	4
50	605	605	0	-61	57
75	1,165	1,192	-27	-927	673
90	3,073	6,925	-3,852	-81,960	12,508

Tennessee Cow Calf Surveillance Zone Day 8: Ideal					
Percentile	Ideal Tracing	Current Tracing	Estimated Difference	ci_lower	ci_upper
10	562	562	0	0	0
25	562	562	0	-1	1
50	605	605	0	-108	82
75	1,160	1,192	-32	-985	735
90	3,290	6,925	-3,635	-78,586	6,326

New Mexico Stocker Surveillance Zone Day 8: Partial					
Percentile	Partial Tracing	Current Tracing	Estimated Difference	ci_lower	ci_upper
10	82	82	0	0	0
25	82	82	0	0	0
50	82	82	0	-2	3
75	136	139	-3	-264	135
90	631	2,275	-1,644	-35,915	3,282

New Mexico Stocker Surveillance Zone Day 8: Ideal					
Percentile	Ideal Tracing	Current Tracing	Estimated Difference	ci_lower	ci_upper
10	82	82	0	0	0
25	82	82	0	0	0
50	82	82	0	-3	2
75	131	139	-7	-237	110
90	500	2,275	-1,775	-41,751	1,072

Outbreaks Detected on Day 14

Nebraska Feedlot Detected Day 14: Partial

Percentile	Partial Tracing	Current Tracing	Estimated Difference	ci_lower	ci_upper
10	5	5	0	-2	2
25	10	10	-1	-5	5
50	27	33	-6	-32	24
75	134	400	-266	-893	117
90	541	1558	-1017	-1670	-148

Nebraska Feedlot Detected Day 14: Ideal					
Percentile	Ideal Tracing	Current Tracing	Estimated Difference	ci_lower	ci_upper
10	5	5	0	-2	2
25	10	10	-1	-5	4
50	23	33	-10	-34	10
75	56	400	-344	-905	-13
90	110	1558	-1447	-1990	-670

Texas Feedlot Detected Day 14: Partial					
Percentile	Partial Tracing	Current Tracing	Estimated Difference	ci_lower	ci_upper
10	8	8	0	-5	4
25	17	20	-3	-14	6
50	43	65	-21	-75	16
75	138	310	-172	-589	15
90	385	1635	-1250	-2301	-48

Texas Feedlot Detected Day 14: Ideal					
Percentile	Ideal Tracing	Current Tracing	Estimated Difference	ci_lower	ci_upper
10	8	8	0	-6	3
25	15	20	-5	-15	5
50	35	65	-30	-84	2
75	86	310	-224	-628	-51
90	161	1635	-1474	-2519	-402

Tennessee Dairy Detected Day 14: Partial					
Percentile	Partial Tracing	Current Tracing	Estimated Difference	ci_lower	ci_upper

10	12	12	0	-8	7
25	25	29	-4	-23	10
50	70	142	-73	-407	20
75	221	1202	-981	-1653	-298
90	596	2374	-1778	-2429	-918

Tennessee Dairy Detected Day 14: Ideal					
Percentile	Ideal Tracing	Current Tracing	Estimated Difference	ci_lower	ci_upper
10	12	12	0	-8	7
25	24	29	-5	-24	8
50	52	142	-91	-398	-6
75	122	1202	-1080	-1684	-446
90	277	2374	-2098	-2792	-1323

California Dairy Detected Day 14: Partial					
Percentile	Partial Tracing	Current Tracing	Estimated Difference	ci_lower	ci_upper
10	35	44	-9	-33	10
25	68	89	-20	-58	11
50	123	183	-60	-119	3
75	224	433	-209	-607	-20
90	582	1124	-542	-1198	75

California Dairy Detected Day 14: Ideal					
Percentile	Ideal Tracing	Current Tracing	Estimated Difference	ci_lower	ci_upper
10	31	44	-13	-35	2
25	52	89	-36	-71	-10
50	86	183	-97	-150	-44
75	153	433	-280	-632	-94
90	243	1124	-881	-1509	-462

New York Dairy Detected Day 14: Partial					
Percentile	Partial Tracing	Current Tracing	Estimated Difference	ci_lower	ci_upper
10	11	14	-3	-14	7

25	42	57	-15	-109	36
50	138	366	-228	-571	-40
75	422	1220	-798	-1307	-384
90	839	2162	-1323	-2092	-554

New York Dairy Detected Day 14: Ideal					
Percentile	Ideal Tracing	Current Tracing	Estimated Difference	ci_lower	ci_upper
10	11	14	-3	-14	6
25	28	57	-30	-111	6
50	62	366	-304	-659	-125
75	128	1220	-1092	-1568	-695
90	210	2162	-1952	-2663	-1249

Nebraska Cow-Calf Detected Day 14: Partial					
Percentile	Partial Tracing	Current Tracing	Estimated Difference	ci_lower	ci_upper
10	1	1	0	0	0
25	2	2	0	-1	1
50	3	4	0	-4	2
75	13	20	-6	-143	24
90	115	711	-596	-1203	138

Nebraska Cow-Calf Detected Day 14: Ideal					
Percentile	Ideal Tracing	Current Tracing	Estimated Difference	ci_lower	ci_upper
10	1	1	0	0	0
25	2	2	0	-1	1
50	3	4	0	-4	2
75	13	20	-7	-138	12
90	80	711	-630	-1354	46

Tennessee Cow-Calf Detected Day 14: Partial					
Percentile	Partial Tracing	Current Tracing	Estimated Difference	ci_lower	ci_upper
10	1	1	0	0	0
25	2	2	0	-1	1

50	5	5	0	-2	3
75	19	20	-1	-40	38
90	161	190	-29	-584	397

Tennessee Cow-Calf Detected Day 14: Ideal					
Percentile	Ideal Tracing	Current Tracing	Estimated Difference	ci_lower	ci_upper
10	1	1	0	0	0
25	2	2	0	-1	1
50	5	5	0	-2	2
75	14	20	-5	-45	15
90	56	190	-134	-715	20

New Mexico Stocker Detected Day 14: Partial					
Percentile	Partial Tracing	Current Tracing	Estimated Difference	ci_lower	ci_upper
10	1	1	0	-1	1
25	2	2	0	0	0
50	5	5	0	-5	3
75	27	39	-12	-191	60
90	253	901	-648	-2001	323

New Mexico Stocker Detected Day 14: Ideal					
Percentile	Ideal Tracing	Current Tracing	Estimated Difference	ci_lower	ci_upper
10	1	1	0	-1	1
25	2	2	0	0	0
50	5	5	0	-6	3
75	16	39	-23	-211	10
90	77	901	-823	-2246	-32

Nebraska Feedlot Duration Day 14: Partial					
Percentile	Partial Tracing	Current Tracing	Estimated Difference	ci_lower	ci_upper
10	26	26	0	-5	5
25	32	35	-3	-11	6
50	54	54	0	-18	17

75	99	152	-53	-149	27
90	180	285	-105	-190	-33

Nebraska Feedlot Duration Day 14: Ideal					
Percentile	Ideal Tracing	Current Tracing	Estimated Difference	ci_lower	ci_upper
10	26	26	0	-5	4
25	31	35	-4	-11	4
50	47	54	-7	-25	6
75	69	152	-83	-173	-9
90	96	285	-189	-247	-122

Texas Feedlot Duration Day 14: Partial					
Percentile	Partial Tracing	Current Tracing	Estimated Difference	ci_lower	ci_upper
10	28	28	-1	-5	4
25	36	42	-6	-18	6
50	64	80	-17	-42	10
75	105	157	-51	-123	5
90	167	261	-95	-179	-5

Texas Feedlot Duration Day 14: Ideal					
Percentile	Ideal Tracing	Current Tracing	Estimated Difference	ci_lower	ci_upper
10	27	28	-2	-6	2
25	36	42	-7	-19	6
50	56	80	-24	-47	-1
75	83	157	-74	-142	-21
90	121	261	-141	-206	-84

Tennessee Dairy Duration Day 14: Partial					
Percentile	Partial Tracing	Current Tracing	Estimated Difference	ci_lower	ci_upper
10	33	35	-1	-9	6
25	44	50	-6	-22	8
50	74	98	-25	-66	4

75	109	217	-107	-144	-38
90	195	262	-67	-137	-20

Tennessee Dairy Duration Day 14: Ideal					
Percentile	Ideal Tracing	Current Tracing	Estimated Difference	ci_lower	ci_upper
10	33	35	-2	-9	5
25	43	50	-7	-22	4
50	65	98	-34	-77	-8
75	84	217	-133	-163	-70
90	117	262	-145	-175	-109

California Dairy Duration Day 14: Partial					
Percentile	Partial Tracing	Current Tracing	Estimated Difference	ci_lower	ci_upper
10	48	54	-6	-19	6
25	64	74	-11	-25	2
50	88	101	-14	-37	4
75	126	183	-57	-123	17
90	214	294	-80	-218	35

California Dairy Duration Day 14: Ideal					
Percentile	Ideal Tracing	Current Tracing	Estimated Difference	ci_lower	ci_upper
10	44	54	-10	-23	2
25	58	74	-16	-29	-6
50	74	101	-27	-47	-12
75	104	183	-79	-145	-15
90	156	294	-138	-291	-59

New York Dairy Duration Day 14: Partial					
Percentile	Partial Tracing	Current Tracing	Estimated Difference	ci_lower	ci_upper
10	32	35	-3	-15	7
25	51	62	-10	-37	11
50	91	130	-39	-102	-7

75	132	251	-119	-155	-52
90	226	309	-83	-187	-37

New York Dairy Duration Day 14: Ideal					
Percentile	Ideal Tracing	Current Tracing	Estimated Difference	ci_lower	ci_upper
10	31	35	-4	-17	6
25	48	62	-14	-40	4
50	66	130	-64	-121	-32
75	90	251	-161	-191	-112
90	120	309	-190	-283	-146

Nebraska Cow Calf Duration Day 14: Partial					
Percentile	Partial Tracing	Current Tracing	Estimated Difference	ci_lower	ci_upper
10	14	14	0	0	0
25	16	16	0	-3	3
50	22	23	-2	-9	4
75	44	49	-5	-49	21
90	87	177	-90	-174	38

Nebraska Cow Calf Duration Day 14: Ideal					
Percentile	Ideal Tracing	Current Tracing	Estimated Difference	ci_lower	ci_upper
10	14	14	0	0	0
25	16	16	0	-4	3
50	21	23	-2	-9	4
75	40	49	-9	-48	16
90	76	177	-101	-190	18

Tennessee Cow Calf Duration Day 14: Partial					
Percentile	Partial Tracing	Current Tracing	Estimated Difference	ci_lower	ci_upper
10	14	14	0	-1	1
25	18	18	0	-5	5
50	27	27	0	-7	6
75	44	47	-2	-32	23
90	111	114	-3	-112	75

Tennessee Cow Calf Duration Day 14: Ideal					
Percentile	Ideal Tracing	Current Tracing	Estimated Difference	ci_lower	ci_upper
10	14	14	0	-1	1
25	18	18	0	-4	4
50	27	27	0	-8	6
75	39	47	-7	-34	9
90	64	114	-50	-132	13

New Mexico Stocker Duration Day 14: Partial					
Percentile	Partial Tracing	Current Tracing	Estimated Difference	ci_lower	ci_upper
10	14	14	0	-2	2
25	18	18	0	-3	3
50	27	28	-1	-12	9
75	60	78	-19	-81	23
90	137	244	-108	-303	71

New Mexico Stocker Duration Day 14: Ideal					
Percentile	Ideal Tracing	Current Tracing	Estimated Difference	ci_lower	ci_upper
10	14	14	0	-2	2
25	18	18	0	-3	3
50	28	28	0	-11	9
75	49	78	-30	-90	6
90	82	244	-163	-315	-19

Number of Farms in Control Areas from Outbreaks Detected on Day 14

Nebraska Feedlot Control Area Day 14: Partial					
Percentile	Partial Tracing	Current Tracing	Estimated Difference	ci_lower	ci_upper
10	420	435	-15	-172	113
25	675	716	-41	-302	250
50	1,723	1,982	-258	-1,878	1,436
75	8,293	24,213	-15,920	-53,330	8,333
90	35,492	100,486	-64,994	-123,629	-3,923

Nebraska Feedlot Control Area Day 14: Ideal					
Percentile	Ideal Tracing	Current Tracing	Estimated Difference	ci_lower	ci_upper
10	420	435	-15	-166	118
25	681	716	-35	-304	256
50	1,535	1,982	-446	-1,931	665
75	3,618	24,213	-20,595	-61,794	-467
90	7,395	100,486	-93,091	-142,692	-43,042

Texas Feedlot Control Area Day 14: Partial					
Percentile	Partial Tracing	Current Tracing	Estimated Difference	ci_lower	ci_upper
10	146	157	-11	-129	97
25	378	509	-130	-565	224
50	1,374	2,410	-1,036	-4,050	827
75	7,300	20,405	-13,105	-39,454	1,238
90	23,802	108,406	-84,604	-152,685	-8,740

Texas Feedlot Control Area Day 14: Ideal					
Percentile	Ideal Tracing	Current Tracing	Estimated Difference	ci_lower	ci_upper
10	130	157	-27	-138	78
25	339	509	-170	-604	134
50	1,037	2,410	-1,374	-4,206	63
75	3,681	20,405	-16,724	-42,621	-2,883
90	6,777	108,406	-101,628	-162,044	-27,584

Number of Farms in Control Areas from Outbreaks Detected on Day 14

Tennessee Dairy Control Area Day 14: Partial					
Percentile	Partial Tracing	Current Tracing	Estimated Difference	ci_lower	ci_upper
10	1,433	1,524	-91	-1,138	920
25	3,274	3,952	-678	-2,936	1,337
50	7,792	16,381	-8,589	-34,459	1,820
75	23,915	93,846	-69,931	-121,088	-25,592
90	52,792	178,086	-125,294	-174,552	-64,905

Tennessee Dairy Control Area Day 14: Ideal					
Percentile	Ideal Tracing	Current Tracing	Estimated Difference	ci_lower	ci_upper
10	1,391	1,524	-133	-1,199	1,391
25	2,554	3,952	-1,398	-3,587	2,554
50	6,678	16,381	-9,703	-38,418	6,678
75	14,644	93,846	-79,202	-127,888	14,644
90	28,986	178,086	-149,100	-193,530	28,986

California Dairy Control Area Day 14: Partial					
Percentile	Partial Tracing	Current Tracing	Estimated Difference	ci_lower	ci_upper
10	1,673	2,368	-695	-1,854	591
25	3,758	4,642	-884	-2,943	777
50	6,388	8,414	-2,026	-4,524	-3
75	10,694	21,180	-10,486	-25,202	-843
90	20,174	49,309	-29,135	-50,500	2,776

California Dairy Control Area Day 14: Ideal					
Percentile	Ideal Tracing	Current Tracing	Estimated Difference	ci_lower	ci_upper
10	1,605	2,368	-763	-1,994	405
25	3,029	4,642	-1,613	-3,380	-252
50	4,869	8,414	-3,545	-6,498	-1,696
75	7,302	21,180	-13,878	-28,760	-4,434
90	11,581	49,309	-37,728	-58,098	-19,774

Number of Farms in Control Areas from Outbreaks Detected on Day 14

New York Dairy Control Area Day 14: Partial					
Percentile	Partial Tracing	Current Tracing	Estimated Difference	ci_lower	ci_upper
10	809	1,064	-255	-1,017	617
25	3,304	4,459	-1,155	-7,454	2,399
50	10,648	26,225	-15,578	-41,149	-1,444
75	31,794	84,197	-52,404	-85,559	-24,496
90	61,938	149,558	-87,621	-151,934	-31,302

New York Dairy Control Area Day 14: Ideal					
Percentile	Ideal Tracing	Current Tracing	Estimated Difference	ci_lower	ci_upper
10	826	1,064	-238	-1,041	424
25	2,174	4,459	-2,285	-7,951	456
50	5,107	26,225	-21,119	-47,109	-7,920
75	10,019	84,197	-74,179	-104,579	-50,258
90	16,091	149,558	-133,467	-192,398	-86,613

Nebraska Cow Calf Control Area Day 14: Partial					
Percentile	Partial Tracing	Current Tracing	Estimated Difference	ci_lower	ci_upper
10	91	91	0	0	0
25	99	99	0	-35	34
50	201	216	-16	-174	105
75	647	1,008	-361	-10,569	1,266
90	6,908	47,138	-40,230	-83,993	9,766

Nebraska Cow Calf Control Area Day 14: Ideal					
Percentile	Ideal Tracing	Current Tracing	Estimated Difference	ci_lower	ci_upper
10	91	91	0	0	0
25	99	99	0	-33	31
50	196	216	-21	-166	85
75	588	1,008	-420	-11,867	992
90	4,979	47,138	-42,159	-85,535	2,054

Number of Farms in Control Areas from Outbreaks Detected on Day 14

Tennessee Cow Calf Control Area Day 14: Partial					
Percentile	Partial Tracing	Current Tracing	Estimated Difference	ci_lower	ci_upper
10	248	248	0	-2	2
25	259	259	0	-19	21
50	570	581	-11	-466	419
75	2,610	2,537	73	-5,940	5,122
90	15,535	19,482	-3,947	-47,230	30,848

Tennessee Cow Calf Control Area Day 14: Ideal					
Percentile	Ideal Tracing	Current Tracing	Estimated Difference	ci_lower	ci_upper
10	248	248	0	-2	2
25	259	259	0	-30	29
50	550	581	-31	-429	343
75	1,879	2,537	-658	-5,731	2,041
90	6,673	19,482	-12,809	-54,510	2,802

New Mexico Stocker Control Area Day 14: Partial					
Percentile	Partial Tracing	Current Tracing	Estimated Difference	ci_lower	ci_upper
10	68	68	0	-1	1
25	75	75	0	-25	30
50	172	182	-9	-229	131
75	1,199	1,913	-714	-12,644	3,325
90	12,029	54,634	-42,606	-102,854	12,556

New Mexico Stocker Control Area Day 14: Ideal					
Percentile	Ideal Tracing	Current Tracing	Estimated Difference	ci_lower	ci_upper
10	68	68	0	-2	2
25	75	75	0	-22	26
50	174	182	-8	-228	128
75	780	1,913	-1,133	-13,612	582
90	3,798	54,634	-50,836	-115,229	-3,218

Number of Farms in Surveillance Zones from Outbreaks Detected on Day 14

Nebraska Feedlot Surveillance Zone Day 14: Partial					
Percentile	Partial Tracing	Current Tracing	Estimated Difference	ci_lower	ci_upper
10	853	866	-13	-277	269
25	1,357	1,388	-31	-509	486
50	3,493	4,012	-519	-4,338	2,904
75	17,361	44,968	-27,607	-93,807	16,075
90	65,638	168,095	-102,457	-199,131	-20,023

Nebraska Feedlot Surveillance Zone Day 14: Ideal					
Percentile	Ideal Tracing	Current Tracing	Estimated Difference	ci_lower	ci_upper
10	853	866	-13	-266	213
25	1,315	1,388	-73	-619	516
50	3,200	4,012	-812	-4,558	1,338
75	7,846	44,968	-37,122	-103,040	-903
90	15,897	168,095	-152,198	-244,634	-82,113

Texas Feedlot Surveillance Zone Day 14: Partial					
Percentile	Partial Tracing	Current Tracing	Estimated Difference	ci_lower	ci_upper
10	295	327	-31	-279	175
25	749	1,060	-311	-1,215	458
50	2,772	4,556	-1,784	-6,719	1,676
75	14,508	39,488	-24,979	-75,224	2,623
90	46,829	183,917	-137,088	-244,204	-4,716

Texas Feedlot Surveillance Zone Day 14: Ideal					
Percentile	Ideal Tracing	Current Tracing	Estimated Difference	ci_lower	ci_upper
10	253	327	-74	-298	128
25	700	1,060	-360	-1,248	349
50	2,097	4,556	-2,459	-8,010	174
75	6,840	39,488	-32,647	-83,136	-6,989
90	14,400	183,917	-169,517	-268,642	-51,091

Number of Farms in Surveillance Zones from Outbreaks Detected on Day 14

Tennessee Dairy Surveillance Zone Day 14: Partial					
Percentile	Partial Tracing	Current Tracing	Estimated Difference	ci_lower	ci_upper
10	3,290	3,335	-45	-2,279	2,039
25	7,235	8,737	-1,503	-6,097	3,061
50	15,701	32,107	-16,406	-59,668	3,234
75	44,943	156,527	-111,584	-201,817	-44,070
90	91,733	297,061	-205,328	-270,601	-110,890

Tennessee Dairy Surveillance Zone Day 14: Ideal					
Percentile	Ideal Tracing	Current Tracing	Estimated Difference	ci_lower	ci_upper
10	3,157	3,335	-178	-2,264	1,236
25	5,721	8,737	-3,016	-7,823	1,067
50	13,983	32,107	-18,123	-60,660	-1,184
75	28,115	156,527	-128,412	-214,067	-63,534
90	54,265	297,061	-242,795	-305,381	-157,455

California Dairy Surveillance Zone Day 14: Partial					
Percentile	Partial Tracing	Current Tracing	Estimated Difference	ci_lower	ci_upper
10	2,750	3,643	-893	-2,582	872
25	5,476	6,386	-910	-2,802	663
50	7,923	9,889	-1,966	-5,143	202
75	12,599	25,657	-13,058	-34,321	-211
90	26,129	70,543	-44,414	-80,641	3,455

California Dairy Surveillance Zone Day 14: Ideal					
Percentile	Ideal Tracing	Current Tracing	Estimated Difference	ci_lower	ci_upper
10	2,583	3,643	-1,060	-2,943	763
25	4,544	6,386	-1,842	-3,598	-570
50	6,574	9,889	-3,315	-6,372	-1,541
75	9,204	25,657	-16,453	-37,105	-4,968
90	15,071	70,543	-55,472	-91,606	-24,087

Number of Farms in Surveillance Zones from Outbreaks Detected on Day 14

New York Dairy Surveillance Zone Day 14: Partial					
Percentile	Partial Tracing	Current Tracing	Estimated Difference	ci_lower	ci_upper
10	1,939	2,294	-356	-2,030	1,241
25	6,771	8,963	-2,192	-11,573	4,503
50	18,971	42,926	-23,955	-66,924	-1,653
75	51,696	138,680	-86,983	-144,293	-40,057
90	105,912	240,582	-134,669	-240,663	-48,372

New York Dairy Surveillance Zone Day 14: Ideal					
Percentile	Ideal Tracing	Current Tracing	Estimated Difference	ci_lower	ci_upper
10	1,918	2,294	-376	-1,867	1,134
25	4,693	8,963	-4,270	-13,057	1,064
50	9,975	42,926	-32,951	-75,413	-11,882
75	18,199	138,680	-120,481	-171,959	-81,439
90	28,027	240,582	-212,555	-313,284	-140,274

Nebraska Cow Calf Surveillance Zone Day 14: Partial					
Percentile	Partial Tracing	Current Tracing	Estimated Difference	ci_lower	ci_upper
10	241	241	0	0	0
25	259	259	0	-75	76
50	489	543	-55	-416	289
75	1,508	2,350	-843	-19,314	2,767
90	13,068	83,865	-70,797	-149,365	16,328

Nebraska Cow Calf Surveillance Zone Day 14: Ideal					
Percentile	Ideal Tracing	Current Tracing	Estimated Difference	ci_lower	ci_upper
10	241	241	0	-1	1
25	259	259	0	-69	66
50	467	543	-76	-439	237
75	1,356	2,350	-994	-17,153	1,551
90	10,042	83,865	-73,824	-151,284	3,075

Number of Farms in Surveillance Zones from Outbreaks Detected on Day 14

Tennessee Cow Calf Surveillance Zone Day 14: Partial					
Percentile	Partial Tracing	Current Tracing	Estimated Difference	ci_lower	ci_upper
10	562	562	0	-3	3
25	583	583	0	-64	59
50	1,243	1,265	-23	-968	945
75	5,574	5,267	307	-10,175	11,552
90	31,294	36,932	-5,638	-79,929	48,317

Tennessee Cow Calf Surveillance Zone Day 14: Ideal					
Percentile	Ideal Tracing	Current Tracing	Estimated Difference	ci_lower	ci_upper
10	562	562	0	-3	3
25	583	583	0	-70	58
50	1,181	1,265	-85	-984	888
75	3,987	5,267	-1,280	-11,198	4,095
90	13,932	36,932	-23,000	-89,067	6,862

New Mexico Stocker Surveillance Zone Day 14: Partial					
Percentile	Partial Tracing	Current Tracing	Estimated Difference	ci_lower	ci_upper
10	82	82	0	-1	1
25	91	91	0	-28	31
50	211	223	-12	-356	222
75	1,915	2,828	-913	-17,751	5,694
90	20,674	93,007	-72,333	-168,033	17,165

New Mexico Stocker Surveillance Zone Day 14: Ideal					
Percentile	Ideal Tracing	Current Tracing	Estimated Difference	ci_lower	ci_upper
10	82	82	0	-2	1
25	91	91	0	-31	31
50	217	223	-7	-359	200
75	1,236	2,828	-1,592	-20,595	1,228
90	5,972	93,007	-87,035	-187,614	-3,758
